# A taxonomic revision of the *Cymindis (Pinacodera) limbata* species group (Coleoptera, Carabidae, Lebiini), including description of a new species from Florida, U.S.A.

**DOI:** 10.3897/zookeys.259.2970

**Published:** 2013-01-18

**Authors:** Wesley M. Hunting

**Affiliations:** 1 National Chung Hsing University, Department of Entomology, 250, Kuo Kuang Rd. Taichung City 402 Taiwan, R.O.C.

**Keywords:** Carabidae, *Cymindis*, *Pinacodera*, taxonomy, identification keys, Nearctic, Region, Neotropical Region

## Abstract

The *Cymindis (Pinacodera) limbata* species group (Coleoptera, Carabidae, Lebiini) is a precinctive New World taxon with ranges extended from portions of temperate southeastern Canada and the U.S.A. through the montane regions of Mexico, south to the Isthmus of Tehuantepec. The group is distinguishable from all other members of the subgenus *Pinacodera* by males possessing a distinctive sclerite (endophallic plate) at the apex of the endophallus. In the past, a lack of material and misunderstandings of range of variation within species have contributed to confusion about how many species there really are.

This revision of the *limbata* species group includes a classification, a key to groups within the subgenus *Pinacodera* and species within the *limbata* group, descriptions of species, re-rankings and new synonymies. In total 10 taxa are treated, with 6 new synonyms proposed, 1 new combination introduced and 1 new species described: *Cymindis (Pinacodera) rufostigma* (type locality: Archbold Biological Station, Highlands County, Florida, U.S.A.). Each taxon is characterized in terms of structural features of adults, habitat, geographical distribution, and chorological affinities. Available ecological information and treatments of variation are included.

## Introduction

**Figure 1.**
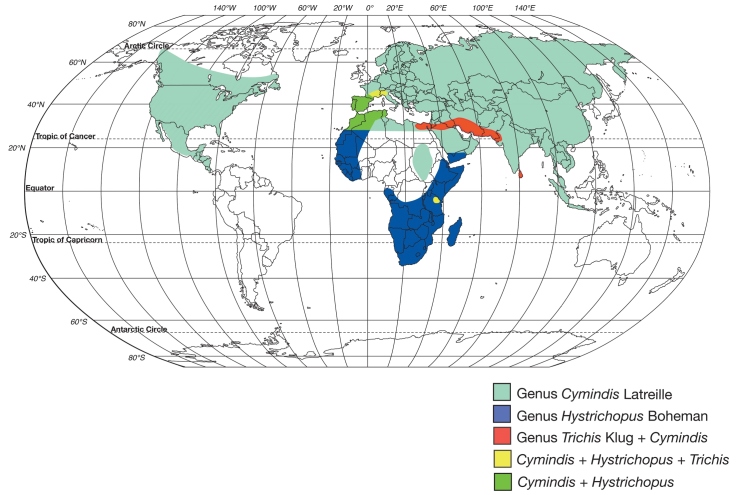
World map showing geographic ranges of the genera in the subtribe Cymindidina (Coleoptera: Carabidae).

**Figure 2.**
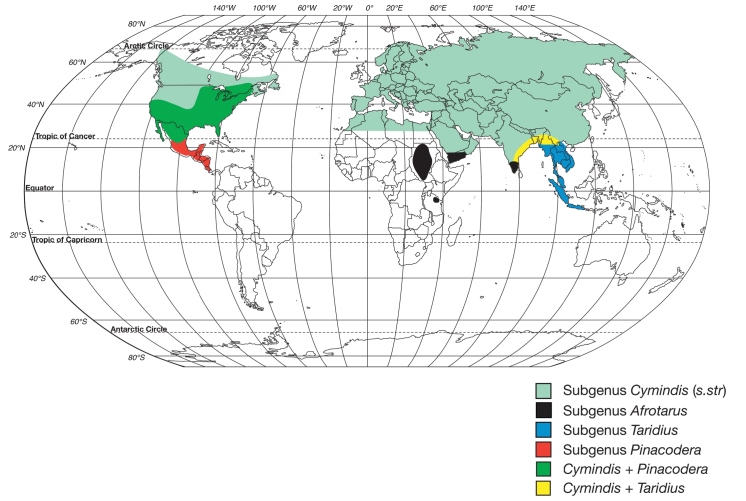
World map showing geographic ranges of the subgenera within the genus *Cymindis* (*s.l.*).

Genera of the subtribe Cymindidina (Lebiini) (Table 1) include the southern Palaearctic-Oriental *Trichis* Klug, the southern Palaearctic-Afrotropical *Hystrichopus* Boheman, and *Cymindis* (*s.l*.) Latreille (Fig. 1). *Cymindis* is by far the most speciose and widespread, with a range that extends to all zoogeographic regions with the exception of Australia (Ball and Hilchie 1983). Species of *Cymindis* are arranged in four subgenera (Fig. 2): *Cymindis* (*s. str.*) Latreille, *Afrotarus* Jeannel, *Taridius* Chaudoir, and *Pinacodera* Schaum. Both *Afrotarus* and *Taridius* are restricted to the Old World, *Cymindis* (*s. str.*) has a primarily Holarctic distribution with a few additional members in portions of the Oriental and northern Neotropical regions, and *Pinacodera* is a Western Hemisphere-restricted taxon with members ranging from southeastern Canada to Costa Rica in Middle America (Fig. 2).

**Table 1.** Classification of supraspecific taxa of the subtribe Cymindidina, tribe Lebiini (Ball and Hilchie 1983).

Family Carabidae
Subfamily Lebiinae
Tribe Lebiini (s. str)
Subtribe Cymindidina
Genus *Trichis* Klug (Ceylonitarus Ball & Hilchie)
Genus *Hystrichopus* Boheman
Subgenus *Assadecma* Basilewsky
Subgenus *Pseudomasoreus* Desbrochers des Loges
Subgenus *Hystrichopus* (s. str) Boheman
Subgenus *Plagiopyga* Boheman
Genus *Cymindis* Latreille
Subgenus *Cymindis* (s. str.) Latreille
Subgenus *Afrotarus* Jeannel
Subgenus *Taridius* Chaudoir
Subgenus *Pinacodera* Schaum
*Cymindis (Pinacodera) limbata* group
*Cymindis (Pinacodera) limbata* complex
*Cymindis complanata* Dejean
*Cymindis limbata* Dejean
*Cymindis rufostigma* Hunting
*Cymindis platicollis platicollis* (Say)
*Cymindis platicollis atripennis* (Casey)
*Cymindis (Pinacodera) punctigera* complex
*Cymindis punctigera punctigera* LeConte
*Cymindis punctigera sulcipennis* (Horn)
*Cymindis (Pinacodera) chevrolati* complex
*Cymindis chevrolati* Dejean
*Cymindis laevior* (Bates)
*Cymindis ruficornis* (Bates)
*Cymindis (Pinacodera) latiuscula* group
*Cymindis (Pinacodera) latiuscula* subgroup
*Cymindis (Pinacodera) chalcea* subgroup
*Cymindis (Pinacodera) basipunctata* subgroup
*Cymindis (Pinacodera) tacanamera* subgroup

*Pinacodera* includes more than 25 taxa arranged in two species groups, the *limbata* and *latiuscula* groups (Ball 2008, personal communication) that are not easily distinguished from one another. The less diverse *limbata* species group with 10 taxa, treated here, has members that are more northern geographically and males that are distinguishable from those of the *latiuscula* group by a sclerite (endophallic plate) located at the apex of the endophallus.

The taxonomic position of *Pinacodera* remains contested to some degree. Initially, *Pinacodera* was introduced and ranked as a genus by Schaum (1857). Schaum divided the genus *Cymindis* Latreille, 1806, after recognizing that specimens of species including *Cymindis limbata* Dejean (the type species), *Cymindis fuscata* Dejean, *Cymindis platicollis* Say and *Cymindis complanata* Dejean differed in having no setae (other than one pair of typical fixed setae located at the apex of each tarsomere) on the dorsal surface of the tarsi. As well, males of these species differed in having expanded meso-tarsi. The name *Pinacodera* (Greek, “flat+neck.”) (Blatchley 1910), also indicates that Schaum recognized species of this group differed in having a pronotum that is flatter than those of *Cymindis* species. More than a century later, *Pinacodera* was ranked as a subgenus (Ball and Hilchie 1983) in the genus *Cymindis* (*s. lat.*) along with *Afrotarus*, *Cymindis* (*s. str.*), and *Taridius*. I accept here the ranking by Ball and Hilchie.

At the time Schaum recognized *Pinacodera*, four other species of the *limbata* group (Table 1) had been described, including *Cymindis platicollis*, *Cymindis complanata*, *Cymindis chevrolati* Dejean, and *Cymindis punctigera* LeConte. In 1878, H. W. Bates described two additional species, *Cymindis amblygona* and *Cymindis angulifera*, but later realized (Bates 1883) that characters used to distinguish the species were variable and accordingly ranked them as varieties of “*Cymindis atrata*” (a junior synonym of *Cymindis chevrolati*). Two more varieties of “*Cymindis atrata*”, *Cymindis ruficornis*, and *Cymindis laevior* were also described by Bates (1891). During the same time period, Horn (1881) described two species of *Pinacodera*, and over a 7-year period in the early twentieth century, Casey (1913, 1920) described 7 more.

I became interested in this group after learning that due to both a lack of specimens available for description and misconceptions of variability in body size, proportions, and coloration, (Casey 1913, 1920) it was probable that several *Pinacodera* taxa were over ranked or incorrectly recognized as valid (Ball 2008, personal communication). A large amount of study material would provide the means to better partition variation within the group. Fortunately, a series of studies of *Pinacodera* are currently being undertaken at the University of Alberta and material had been borrowed from institutional collections from across the United States and Canada. Because of this, thousands of specimens have been available and a thorough examination of the taxa has been possible, using morphological features.

A cursory examination of the literature also revealed a distinct shortage of natural history information for the group despite the abundance of several of the species (Mahar 1978, Liebherr and Mahar 1979). Because of the large numbers of individuals in certain areas, it can be assumed that *Pinacodera* taxa impact the ecosystems they inhabit (Mahar, 1978). This is another good reason why closer examination was warranted and fieldwork was desirable.

The following revision includes taxonomic treatments (descriptions and illustrations of structural features, notes about habitat, geographical variation and distribution, and postulated evolutionary and chorological affinities), and keys for identification of adults. In total 10 taxa are treated, with 6 new synonymies proposed, 1 new combination introduced and 1 new species described. Variation in wing length, body length, mental tooth form, and male genital characters receive detailed treatment.

## Material and methods

### Material

This revision is based on the study of more than 4000 adult specimens representing ten taxa belonging to the subgenus *Pinacodera*. Specimens of a few taxa were collected over the past two years or were already housed at the Strickland Museum, University of Alberta (UASM). Additional adult specimens were borrowed from the collections of various individuals and institutions listed below, along with a four-letter coden (Arnett et al. 1993) used to identify sources of specimens. Names in parentheses below, indicate curator or owner of collection.

ABSC Archbold Biological Station Collection, P. O. Box 2057, Lake Placid, Florida 32852-2057. (M. Deyrup)

AMNH Department of Entomology, American Museum of Natural History, Central Park West, 79th Street, New York, New York, U.S.A., 10024-5192. (L. H. Herman)

ANSP Department of Entomology, The Academy of Natural Sciences, 1900 Benjamin Franklin Parkway, Philadelphia, Pennsylvania, U.S.A. 19103. (D. Otte)

CASC Department of Entomology, California Academy of Sciences, Golden Gate Park, San Francisco, California, U.S.A., 94118. (D. H. Kavanaugh)

CDAE California State Collection of Arthropods, Analysis and Identification Unit, California Department of Food and Agriculture, 1220 N St., Rm. 340, Sacramento, California, 95814. (M. S. Wasbauer)

CIDA Albertson College of Idaho Collection, Orma J. Smith Museum of Natural History, Albertson College of Idaho, Caldwell, Idaho, 83605. (W. H. Clark)

CMNC Entomology Division, Canada Museum of Nature, P.O. 3443 Station D, Ottawa, Ontario, Canada, K1P 6P4. (R. S. Anderson)

CMNH Section of Invertebrate Zoology, Carnegie Museum of Natural History, 4400 Forbes Avenue, Pittsburgh, Pennsylvania, U.S.A. 15213-4080. (R. L. Davidson)

CNCI Canadian National Collection of Insects, Invertebrate Biodiversity Centre, Agriculture and Agri-food Canada, Ottawa, Ontario, Canada, K1A 0C6. (Y. Bousquet)

CUIC Department of Entomology, Comstock Hall, Cornell University, Ithaca, New York, U.S.A., 14853-2601. (J. K. Liebherr)

DAHC Drew A. Hildebrandt Collection, 710 Laney Road, Clinton, Mississippi, U.S.A., 3905. (D. Hildebrandt)

EMEC Essig Museum of Entomology, University of California, Berkeley, California, U.S.A., 94720. (J. A. Chemsak - deceased)

FMNH Field Museum of Natural History, 1400 S. Lake Shore Dr., Chicago, IL, U.S.A. 60605-2496. (M. Thayer, A. Newton, Jr.)

FSCA Florida State Collection of Arthropods, 191 SW 34th Street, P.O. Box 147100, Gainesville, Florida, U.S.A., 32601. (R. E. Woodruff)

INHS Illinois Natural History Survey, 1816 South Oak Street, MC 652, Champaign, Illinois, U.S.A., 61820. (L. M. Page)

IRCW Department of Entomology, University of Wisconsin-Madison, 237 Russell Laboratories, 1630 Linden Dr, Wisconsin, U.S.A., 53706-1598. (D. K. Young)

ISUI Department of Entomology, Iowa State University, Insectary Building, Ames, Iowa, U.S.A., 50011-3140. (R. E. Lewis)

JEWC James E. Wappes Collection, Rt. 2, Box 16BB, Atwater Road, Chadds Ford, Pennsylvania, 19317. (J. E. Wappes)

KSUC Department of Entomology, Kansas State University, 123 W. Waters Hall, Manhattan, Kansas, U.S.A. 66502. (H. D. Blocker)

LACM Los Angeles County Museum of Natural History, 900 Exposition Blvd., Los Angeles, California, U.S.A., 90007. (J. P. Donahue)

MCZC Department of Entomology, Museum of Comparative Zoology, Harvard University, 26 Oxford Street, Cambridge, Massachusetts U.S.A., 02138. (P. D. Perkins, B. D. Farrell)

MNHP Department of Entomology, Natural History Museum, Paris 75005, France. (T. Deuve)

MSUC Department of Entomology, Michigan State University, East Lansing, Michigan 48824-1115. (F. W. Stehr)

MTEC Montana State University Entomology Collection, Entomology Research Laboratory, Montana State University, Bozeman, Montana, 59717. (M. A. Ivie)

NDSU North Dakota State Insect Reference Collection, Entomology Department Collection, North Dakota State University, Fargo, North Dakota 58102. (D. A. Rider)

NHMW Naturhistorisches Museum Wien, Postfach 417, Burgring 7, 1040 Wien. (M. Fischer)

OSEC Oklahoma State University, Entomology and Plant Pathology, 127 NRC, Stillwater, Oklahoma, U.S.A., 74078. (D. C. Arnold)

OSUC Department of Entomology, Ohio State University, 318 West 12th Avenue, Aronoff Laboratories, Columbus, Ohio, U.S.A., 43210. (N. Johnson)

OSAC Department of Entomology, Oregon State University, Corvallis, Oregon, 97331. (D. Maddison)

OXUM Hope Entomological Collections, University Museum, Parks Road. Oxford, Oxfordshire OX1 3PW, England, UK. (G. C. McGavin)

PURC Department of Entomology, Purdue University, 901 W. State Street, West Lafayette, Indiana, U.S.A., 47907-2089. (W. P. McCafferty)

RTBC Marsh Life Science Building 120a, University of Vermont, Burlington, Vermont, 05405. (R. T. Bell)

TAMU Department of Entomology, Texas A&M University, 412 Heep Center, College Station, Texas, U.S.A., 77843-2475. (H. R. Burke)

TTRS Tall Timbers Research Station and Land Conservancy, 13093 Henry Beadel Drive, Tallahassee, Florida, U.S.A., 32312. (J. Cox)

UCRC Department of Entomology, University of California Riverside, Riverside, California, U.S.A., 92521. (S. I. Frommer)

UMMZ Department of Entomology, University of Michigan Museum of Zoology, 1109 Geddes Ave, Ann Arbor, Michigan, U.S.A., 48109-1079. (M. F. O’Brien)

UMSP Department of Entomology, University of Minnesota Insect Collection, 219 Hodson Hall, 1980 Folwell Ave., St. Paul, Minnesota, 55108. (P. J. Clausen)

UNAM Coleccion Entomologica, Instituto de Biologia, Universidad Nacional Autonoma de Mexico, APDO. Postal 70133, 04510 Mexico, D. F. (H. Brailovsky Alperowitz)

USNM Department of Entomology, United States National Museum of Natural History, Smithsonian Institution, Washington, D.C., U.S.A., 20560. (T. L. Erwin)

VMNH Virginia Museum of Natural History, 1001 Douglas Avenue, Martinsville, Virginia 24112. (R. L. Hoffman)

WSUC Department of Entomology, Washington State University – Pullman, Washington, U.S.A., 99163. (R. S. Zack)

All specimens have been databased and that information incorporated into the University of Alberta, E. H. Strickland Virtual Entomology Museum Database, accessed at: http://www.entomology.museums.ualberta.ca

## Methods

### Field work

Despite the wealth of pinned material available for this revision, it became apparent that one species (*Cymindis complanata*) was underrepresented with only ~20 specimens available, many associated with no more label data than abbreviation of a state name. Considering the inadequate data associated with these specimens, new material was very desirable.

Our first attempt (late June, 2007) at collecting specimens of species in the *limbata* group (Table 1) took George E. Ball, Norman Omoth, and myself, to several U.S. State Parks (Table 2) within the known range and habitat of several species in the *limbata* group. We were not able to collect *Cymindis complanata* during this trip but we did collect other species in the *limbata* group including, *Cymindis limbata*, *Cymindis platicollis platicollis*, and *Cymindis platicollis atripennis*. At localities in Pennsylvania, Virginia, and North Carolina (Table  2), specimens of *Cymindis limbata* and *Cymindis platicollis platicollis* were found at night, in mixed forest, primarily on the trunks of aspen and oak trees more than 60 cm in diameter.

At the time, the localities visited in Florida were very dry and specimens of *Cymindis platicollis atripennis* were not active. We were fortunate to talk with P. E. Skelley (FSCA) who told us he had found *Cymindis platicollis atripennis* while surveying squirrel nest insect fauna (Skelley 2007, personal communication). Almost all (16 of 18) individuals of *Cymindis platicollis atripennis* collected during the trip were taken from squirrel nests.

A few months later Drew Hildebrandt (DAHC) caught two specimens at an ultraviolet (U.V.) light trap while on a collecting trip to George L. Smith State Park, Georgia, U.S.A. Based on this, in mid-February of 2008, Omoth and I collected at George L. Smith using sugar-bait on tree trunks (recipe in collecting methods). Less than an hour after our search began we encountered our first *Cymindis complanata* specimen. Over the next several days we collected a total of 13 specimens of *Cymindis complanata* in mixed forest of oak and pine, effectively increasing the known number of pinned adults by one third. All *Cymindis complanata* were collected from slash pine (*Pinus elliottii* Engelm.). Previous to this the only known habitat data for this species was from one specimen labelled “under loose bark of live loblolly pine”, (*Pinus taeda* L.) a tree species that is a close relative of slash pine (Petrides and Wehr 1998). With the ample number of hiding spots provided by the tree’s flaky bark, along with the ferruginous surface of both the tree bark and the dorsal surface of *Cymindis complanata* (Fig. 4), it seems possible that this species has come to rely on trees of these species for camouflage and perhaps uses them for other purposes as well.

**Table 2.** Localities and number of specimens of *Cymindis (Pinacodera) limbata* species group captured during 2007 and 2008 collecting trips. (See text for further details).

**Collecting Locality (U.S.A.)**	**Year**	**Taxon**			
		***Cymindis complanata***	***Cymindis limbata***	***Cymindis platicollis platicollis***	***Cymindis platicollis atripennis***
Archbold Biological Station, FL	2007, 2008	0	0	0	0
Big Bend State Park, TX	2007	0	0	0	0
Blackwater River State Park, FL	2007	0	0	0	0
Brazos Bend State Park, TX	2007	0	0	0	0
Fairy Stone State Park, VA	2007	0	5	1	0
Fort Cooper State Park, FL	2008	0	0	0	0
Gainesville, FL	2007	0	0	0	13
George L. Smith State Park, GA	2008	13	0	0	98
Gold Head Branch State Park, FL	2007, 2008	0	0	0	1
Guadalupe River State Park, TX	2007	0	0	0	0
Highlands Hammock State Park, FL	2008	0	0	0	0
Kerr Lake, NC	2007	0	10	1	0
Manatee Springs State Park, FL	2007, 2007	0	0	0	0
Myakka River State Park, FL	2007, 2008	0	0	0	44
Neuse harbor, NC	2007	0	25	0	0
O'Leno State Park	2007	0	0	0	0
Ochlockonee State Park, FL	2007	0	0	0	3
Ohoopee Dunes State Natural Area, GA	2008	0	0	0	0
Payne's Prarie State Park, FL	2007	0	0	0	0
Powdermill Nature Reserve, PA	2007	0	27	14	0
Tall Timbers Research Station, FL	2008	0	0	0	29

### Collecting methods

Collecting methods for this revision included: u.v. light trapping, beating vegetation, hand collecting from trees, and sugaring tree trunks. The most effective of these methods by far was the use of sugaring. Lindroth (1969) indicated that *Cymindis limbata* had been recorded in numbers on sugar baits used for catching moths. I was able to confirm this after speaking with lepidopterist J. E. Rawlins (CMNH), who had observed similar activity while collecting with these baits. After some experimentation we found the following recipe to be effective. In a pot, mix two liters of red table wine, one can of beer, three packets of Fleischmanns instant-rise yeast, and four to six pounds of brown sugar. Stir well and bottle. Let stand with the cap off for several hours. Before use shake well to mix settled sugar and apply to trees at shoulder height with a wide paintbrush.

### Preparation and examination of adults

Standard methods were used for mounting, dissecting, preparing genitalia, and other technical methods (Ball and Hilchie 1983, Frania and Ball 2007). Genitalia and other small structures were preserved in glycerine and stored in microvials that were pinned beneath the specimen from which they had been removed. Larger structures, including hind wings, were glued to cards and pinned beneath the specimens from which they had been removed.

### Images and illustrations

Photographs of species habitus (Figs 4, 8, 12, 16, 18, 22, 23, 30, 32, 33) were taken at up to 50× using a Nikon Coolpix 8400 mounted on an Olympus SZX16 trinocular stereoscopic microscope and stacked images were combined using Helicon Focus 4.48 (Helicon Soft Ltd., Kharkov, Ukraine). Line drawings of selected body parts (Figs 5, 6, 9, 10, 11, 13, 14, 15, 20, 24, 26, 27, 28, 31, 34, 35, 36, 37, and 39) were prepared using a camera lucida mounted on a Wild M5 stereoscopic microscope. Pronota (Figs 5, 9, 13, 24, 31) were illustrated by Diane Hollingdale (Edmonton, Alberta). Plates were prepared using Adobe Illustrator 11.0 (Adobe Systems, Inc., Mountainview, CA). Geographic range maps (Fig. 7, 17, 29, 41) were prepared using ArcGIS (Environmental Systems Research Institute 2008); the projection used is NAD Lambert Conformal Conic, 1983.

### Measurements

Measurements were made at 12×, 25×, and 50× with a Wild M5 stereoscopic microscope fitted with an ocular micrometer. Various measurements are expressed in the text by abbreviations previously used by Ball and Shpeley (2005):

HL Length of head, measured on left side, from base of left mandible to posterior margin of compound eye.

HW Width of head, maximum transverse distance across head, including eyes.

PL Length of pronotum along midline.

PWM Maximum width of pronotum.

ML Metepisternum length.

MW Metepisternum width.

EL Length of elytra from basal ridge to apex.

EW Maximum width of elytra.

OBL Overall body length.

The shape of the head, pronotum, and metepisternum is shown by the ratio of the width over length (**HW/HL**; **PWM/PL**, **ML/MW**), and elytral shape is indicated by the ratio of the length to the width (**EL/EW**).

To indicate range in body size of each species, the overall body length (**OBL**) was measured (to the nearest 0.5 mm) from the apex of the extended mandibles to the apex of the elytra of both the largest and smallest individual of the species (Frania and Ball 2007).

Size of male genitalia was measured by drawing a straight line between the apical area and the basal lobe of the phallus.

### Notes about synonymy

I rely on Lindroth (1955) and Lindroth and Freitag (1969) for information about type material for Say and Dejean names. Information about material of other authors was taken from notes by G. E. Ball (2008 personal communication).

### Terms

**Figure 3.**
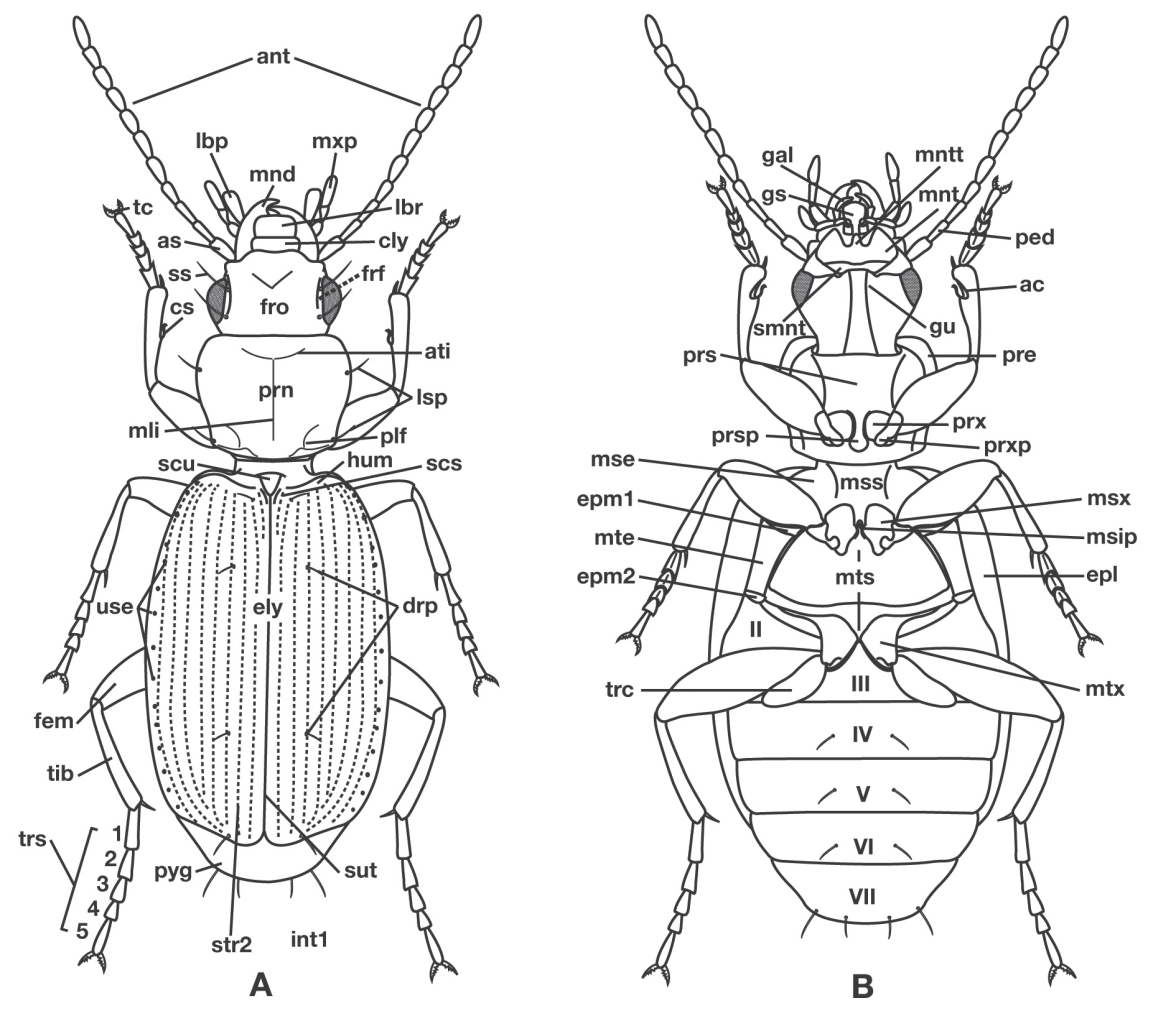
Structure of a generalized lebiine ground beetle (Carabidae) A, dorsal view. B, ventral view. Adapted from Lindroth,1969: XII.

Terms used for structural characters follow Ball and Hilchie (1983) and other authors (See also Figure 3 and Table 3). For some characters of the endophallus of males, no nomenclature has been developed, so in these instances I have used informal descriptive words or phrases.

**Table 3.** Legend for Figure 3.

**ac**, antennal cleaner
**ant**, antenna
**as**, antennal scape (antennomere 1)
**ati**, anterior transverse impression of pronotum
**cly**, clypeus
**cs**, clypsetae of antenna cleaner
**drp**, discal punctures of elytra
**ely**, alytra
**epl**, epipleuron of elytron
**epm1**, epimeron of mesosternum
**epm2**, epimeron of metasternum
**fem**, femur
**frf**, frontal furrow
**fro**, frons
**gal**, galea
**gu**, gula
**gs**, glossal sclerite
**hum**, humerus
**lbp**, labial palpus
**lbr**, labrum
**lsp**, lateral setae of pronotum
**mli**, median longitudinal impression
**mnd**, mandible
**mnt**, mentum
**mntt**, mental tooth
**mse**, mesepisternum
**msip**, mesosternal intercoxal process
**mss**, mesosternum
**msx**, mesocoxa
**mte**, metepisternum
**mts**, metasternum
**mtx**, hindcona
**mxp**, maxillary palpus
**ped**, pedicel (antennomere 2)
**plf**, posteriolateral fovea of pronotum
**pre**, proepipleuron
**prn**, pronotum
**prs**, prosternum
**prsp**, prosternal intercoxal process
**prx**, forecoxa
**prxp**, forecoxal process
**pyg**, pygidium (=tergum VII)
**scs**, scutellum
**smnt**, submentum
**ss**, supraorbital setae
**sut**, suture of elytra
**tc**, tarsal claw
**tib**, tibia
**trc**, trochanter
**trs**, tarsus, (labeled 1-5)
**ues**, umbilical estae of elytra
**int1**, elytral interval 1
**str2**, elytral stria 2
**II-VII**, pregenital sterna

### Label data

All material has been databased and incorporated into the University of Alberta’s Strickland Virtual Entomology Museum Database. This database includes UASM reference numbers, full locality data, date of collection, collectors, and codens.

For type material, information from each label is reproduced using ordinary type. Information on each label is contained in quotation marks, with a semicolon marking the end of each label. Information on color of paper (other than white), printing (other than black), form of paper (other than rectangular), and coden for the collection in which material is housed, is contained in square brackets.

## Taxonomic treatment of taxa of the *limbata* species group, subgenus *Pinacodera* Schaum

### Subgenus Cymindis (Pinacodera)

Schaum, 1857

#### Type species.

*Cymindis limbata* Dejean 1831: 32 (designated by Lindroth 1969: 1067). – LeConte 1861: 24. – Chaudoir 1875: 2. – Horn 1881: 156. – 1882: 146. – LeConte and Horn 1883: 45. – Bates 1883: 187–188. – 1884: 296. – Blatchley 1910: 142, 152. – Leng 1920: 67. – Casey 1920: 279. – Csiki 1932: 1487. – Blackwelder 1944: 62. – Jeannel 1949: 878. – Ball 1960: 161. – Lindroth 1969: 1067–1070. Erwin et al. 1977: 4, 58. – Bousquet and Larochelle 1993: 268. – Ciegler 2000: 119. – Ball and Bousquet 2001: 111. – Lorenz 2005: 465–466.

*Planesus* Motschulsky 1864: 240 (table). Type species: *Cymindis fuscata* Dejean 1831: 321 (= *Cymindis platicollis* Say, 1823) (original designation by Motschulsky 1864: 240 (table)).

#### Taxonomic notes.

The following key, based in part on the unpublished work of Hilchie and Ball (used with their permission), indicates a partial, rudimentary classification of the Western Hemisphere cymindidines, distinguishing adults of subgenus *Pinacodera* from those of subgenus *Cymindis*. Also proposed are the tentative species groups and subgroups of *Pinacodera* and the species complexes of the *limbata* species group, and their species.

##### Key to supraspecific taxa of subgenus *Pinacodera*, and to species of the *Cymindis (Pinacodera) limbata* group, based on characters of adults.

**Table:** 

1	Middle and hind tarsomeres dorsally with six or more setae. Hind femur, anterior surface (ventral in pinned specimens) ventrad with row of three or more long setae. Male middle tarsi without biseriate adhesive setae ventrally; fore tarsomeres 1–3 with biseriate adhesive setae ventrally	subgenus *Cymindis* (s. str.)
1’	Middle and hind tarsomeres dorsally with four or fewer setae. Hind femur, anterior surface ventrad, with two long setae. Male middle tarsomeres 1–3 with biseriate adhesive setae ventrally; fore tarsomeres 1–4 with biseriate adhesive setae ventrally	subgenus *Pinacodera* (2)
2(1’)	Elytra densely, uniformly punctate and setose, concolorous, rufopiceous	*Cymindis (Pinacodera) latiuscula* subgroup
2’	Elytra glabrous to densely setose, punctures sparse to dense, or not evenly distributed, color various, with or without metallic sheen	3
3(2’)	Elytra with shallow depression posteriad, extended from suture to interval 5. Brachypterous, metepisternum nearly quadrate	*Cymindis (Pinacodera) tacanamera* subgroup
3’	Elytra in posterior one third plane, without shallow depression. Macropterous or brachypterous, with metepisternum distinctly longer than wide at base	4
4(3’)	Legs with femora and tibiae rufo-piceous to black *Cymindis (Pinacodera) limbata* group (in part), *Cymindis chevrolati* complex	5
4’	Legs rufo-testaceous to rufous	7
5(4)	Elytra with erect pilose setae extended over entire dorsal surface. Geographic range restricted to Sierra de Atoyac (Sierra Madre del Sur), in eastern Guerrero, Mexico (Fig. 41)	*Cymindis ruficornis* (Bates)
5’	Elytra without erect pilose setae or with only very short setae, hardly visible at 50× magnification. Not found in the Sierra de Atoyac	6
6(5’)	Geographic range restricted to Mexico north of the Sierra Transvolcanica east and west (Fig. 41). Males with microtrichial patch located on dorsal surface of basal lobe of endophallus (Fig. 34A). Female gonocoxite 2 short and stout in form (Fig. 35A)	*Cymindis chevrolati* Dejean
6’	Geographic range restricted to Mexico south of the Sierra Transvolcanica east and west (Fig. 41). Males without microtrichial patch on endophallus (Fig. 34B). Female gonocoxite 2 long and narrow in form (Fig. 35B)	*Cymindis laevior* (Bates)
7(4’)	Elytra metallic green, intervals rather densely and evenly punctate throughout	*Cymindis (Pinacodera) chalcea* subgroup
7’	Elytra rufous to piceous in color, not metallic	8
8(7’)	Elytra basally with intervals moderately densely punctate, but apicad less dense, and impunctate on apical declivity	*Cymindis (Pinacodera) basipunctata* subgroup
8’	Elytra with intervals moderately densely punctate to sparsely punctate, but apical declivity punctate	*Cymindis (Pinacodera) limbata* group (in part) (9)
9(8’)	Elytra with all intervals with one row of irregularly spaced punctures	10
9’	Elytra with intervals having various combinations of irregularly spaced punctures but interval 8 with two or more rows of irregularly spaced punctures	11
10(9)	Most specimens with two to several rugulose transverse lines on dorsal surface of head between eyes (Fig. 25B), dorsal coloration rufo-piceous. Geographic range restricted to the Baja California Peninsula (Fig. 29)	*Cymindis punctigera sulcipennis* (Horn)
10’	Dorsal surface of head between eyes with one shallow transverse line or smooth (Fig 25A). Geographic range north and east of the Baja California Peninsula and south into mainland Mexico (Fig. 29)	*Cymindis punctigera punctigera* LeConte
11(9’)	Humeral macula of elytron with a testaceous patch extended from interval 6 (rarely 5) to lateral margin and extended posteriorly as far as one quarter (0.25) the length of the elytra (Fig. 8)	*Cymindis limbata* Dejean
11’	Elytra without testaceous macula or if present very indistinct and not extended to lateral margin	12
12(11’)	Elytral epipleuron setose (easily observed at 25×) from base of basal constriction and elytra dorsally with one to two irregular rows of punctures on intervals 3, 5 and 7 and two to four rows in intervals 2, 4, 6, and 8	*Cymindis complanata* Dejean
12’	Elytral epipleuron glabrous, or if setose not extended beyond base of basal constriction. Entire dorsal surface with erect pilose setae and punctures somewhat dense and evenly arranged (two to three rows per interval)	13
13(12’)	Dorsal surface uniformly colored with exception of translucently bordered pronotum and elytra	*Cymindis platicollis platicollis* (Say)
13’	Dorsal surface bicolored: head and pronotum contrasted with elytral coloration	14
14(13’)	Head and pronotum evenly rufous to rufo-brunneous; elytra evenly brunneo-piceous to rufo-piceous	*Cymindis platicollis atripennis* (Casey)
14’	Head and pronotum rufo-testaceous and elytra bicolored, piceous with rufo-testaceous macula medially (Fig. 18)	*Cymindis rufostigma* sp. n.

### Cymindis (Pinacodera) limbata species group

#### Diagnosis.

With character states of the subgenus *Pinacodera* restricted as follows. Males of this group are distinguishable by a sclerite (endophallic plate) located at the apex of the everted endophallus (Figs 6, 10, 14, 20, 26, 27, 34)

#### Description.

OBL 7.75 – 13.50 mm. Length (n= 90 males, 90 females): head 0.92 – 1.24, pronotum 1.40 – 2.60, elytra 4.41 – 7.17, metepisternum 0.86 – 1.70 mm; width: head 1.60 – 2.60, pronotum 1.80 – 2.60, elytra 3.16 – 5.42, metepisternum 0.52 – 1.02 mm.

*Body proportions*. HW/HL 1.88 – 2.31; PWM/PL 1.26 – 1.37; EL/EW 1.25- 1.43; ML/MW 1.66 – 2.00.

*Color*. Piceous to testaceous.

*Microsculpture*. Most specimens with microlines not visible on dorsum of head capsule; few with mesh pattern isodiametric to transverse between eyes. Elytra with mesh pattern isodiametric, microlines absent from apical half to moderately deep throughout.

*Macrosculpture and pilosity*. Head ventrally with evenly scattered setigerous punctures bearing setae or not. Pronotum with shallow to moderately deep scattered setigerous punctures, bearing pilose setae or not. Elytra with scattered setigerous punctures, pilose or not. Elytral epipleuron glabrous to moderately setose.

*Fixed setae*. Two pairs of supraorbital setae; clypeus with two lateral setae. Labrum with six setae along apical margin. Pronotum with two to four setae along each margin. Elytra with two setae in stria 3 and one posterior to end of stria 3; one seta at apex of interval 2; 14–18 lateral (umbilical) setae in the ninth interval**;** two setae on each of abdominal sterna III to VI (Fig. 3); four to six setae along apical margin of sternum VII (Fig. 3).

*Luster*. Head capsule and pronotum glossy, elytra glossy to moderately dull; ventral thoracic sterna and abdominal sterna glossy.

*Head*. Eyes, labrum, labium, and palpi, typical for Cymindidina.

*Pronotum*. Anterior and posterior transverse impression shallow to moderately deep; median longitudinal impression moderately shallow; posteriolateral angles right-angled to rounded.

*Elytra*. Humeri broadly to narrowly rounded; striae moderately to shallowly impressed; lateral margin smooth, rounded and widened preapically; elytral apices sinuately subtruncate.

*Hind wings*. Macropterous to brachypterous.

*Legs*. Tarsal claws pectinate, five to seven denticles per claw. Males with adhesive vestiture ventrally, two rows of squamo-setae on tarsomeres 1–4 of foreleg and 1–3 of middle leg.

*Male genitalia*. Anopic. Phallus cylindrical. Endophallus with distinct sclerite (endophallic plate) at apex.

*Female genitalia*. Gonocoxite 2 (**gc2**) short and stout to long and narrow.

#### Habitat.

Adults of this species group have been collected in temperate deciduous, coniferous and mixed forests, subtropical broadleaf forest, tropical montane forests, and acacia scrub environments. Adults are recorded from elevations ranging from sea level to ~3400 m.

#### Geographical distribution.

The range of this species group (Fig. 7, 17, 21, 29, 41 extends in eastern Canada from southern Quebec west to southern Ontario; in the United States from the Atlantic coast south to southern Florida and west to California. In Mexico, it is known throughout the montane regions, north of the Isthmus of Tehuantepec.

#### Chorological affinities.

Species of the *limbata* group are sympatric with members of the subgenus *Cymindis* (*s.str*) (Fig. 2) in portions of southeastern Canada, the U.S.A, and Mexico, and with the *latiuscula* group in portions of the southwestern United States and Mexico.

#### Taxonomic composition, and sequence of presentation.

This group includes ten taxa in three species complexes (treated below). The sequence of presentation is based on geographic distribution of these three complexes, beginning in Eastern North America with the *limbata* complex; second, the monospecific primarily southwestern *punctigera* complex; and third, the northern Mexican *chevrolati* complex. Within each complex, the included species (or subspecies) treatments are in a sequence reflecting my preliminary thoughts about relationships, beginning with the most primitive and ending with the structurally most derived.

### Cymindis (Pinacodera) limbata complex

Figs 4 [Fig F5] [Fig F6] [Fig F7] [Fig F8] [Fig F9] [Fig F10] [Fig F11] [Fig F12] [Fig F13] [Fig F14] [Fig F15] [Fig F16] [Fig F17] [Fig F18] [Fig F19] [Fig F20] –21

#### Diagnosis.

Species in the *limbata* complex are distinguished by pale pronotal and elytral margins; several rows (two to four) of setigerous punctures extended the length of elytral interval 8. Most specimens of species in the *limbata* complex have contrasting punctation in alternate elytral intervals; intervals 1, 3 and 5 typically with one to two rows, interval 2, 4 and 6 typically with two to three rows. All species are macropterous.

#### Description.

With character states of *limbata* species group, restricted as follows.

*Color*. Dorsum of head, pronotum, and elytra testaceous to rufo- piceous, antennae testaceous to rufo-testaceous, palpi testaceous, abdominal sterna and other thoracic sclerites testaceous to rufo-piceous.

*Microsculpture*. Head capsule and pronotum smooth, microlines not evident at 50×. Elytra with mesh pattern isodiametric, microlines moderately deep.

*Macrosculpture and pilosity*. Head capsule with shallow, evenly scattered setigerous punctures on dorsal surface from constriction of neck extended anteriorly toward clypeus. Elytra with striae shallowly impressed and punctulate throughout length; intervals almost flat to slightly convex (few with greater convexity in intervals 1, 3 and 5), one-two (mostly one) irregular rows of fine punctules extended the length of intervals 1, 3 and 5; two-three or three (mostly two) irregular rows of fine punctules extended the length of intervals 2, 4 and 6; interval 8 with two to four rows of fine punctules extended the length of the interval. Abdominal sterna with fine pilose punctures throughout.

*Fixed setae*. Elytra with two setae in stria 3 and one posterior to end of stria 3; one seta at apex of interval 2; 15–17 lateral umbilical setae**.**

*Pronotum*. Anterior and posterior transverse impression shallow; median longitudinal impression shallow; posteriolateral angles almost right- angled to rounded; posterior margin slightly lobate.

*Hind wings*. Macropterous.

*Male genitalia*. Phallus anopic, cylindrical. Endophallus with a flat to slightly curved sclerite (endophallic plate) (Lindroth 1969: 1080–1081) apically. Endophallus with or without microtrichial patch on basal lobe of everted sac.

*Female genitalia*. Gonocoxite 2 (**gc2**) short and stout to long and narrow. Internal genitalia with long cylindrical spermatheca (**sp**), associated spermathecal gland (**sg**), and spermathecal diverticulum (**sd**) located at base of spermathecal gland duct (**sgd**).

#### Geographical distribution.

The range of the *limbata* complex extends in eastern Canada from southern Quebec west to southern Ontario; in the eastern United States from Maine south to southern Florida, west to eastern Colorado and Nebraska, and south to southern Texas. In Mexico it is known from Nuevo Leon, Mexico, in the northern portion of the Sierra Madre Oriental.

#### Chorological affinities.

The geographical range of the *limbata* complex overlaps that of the *punctigera* complex and the *chevrolati* complex along the extreme southern and southwestern portion of its range.

#### Taxonomic composition.

Five taxa are included in this complex: *Cymindis complanata* Dejean; *Cymindis limbata* Dejean; *Cymindis platicollis platicollis* (Say); *Cymindis platicollis atripennis* (Casey); and *Cymindis rufostigma* sp. n.

### Cymindis (Pinacodera) complanata

Dejean

http://species-id.net/wiki/Cymindis_complanata

**Figure 4.**
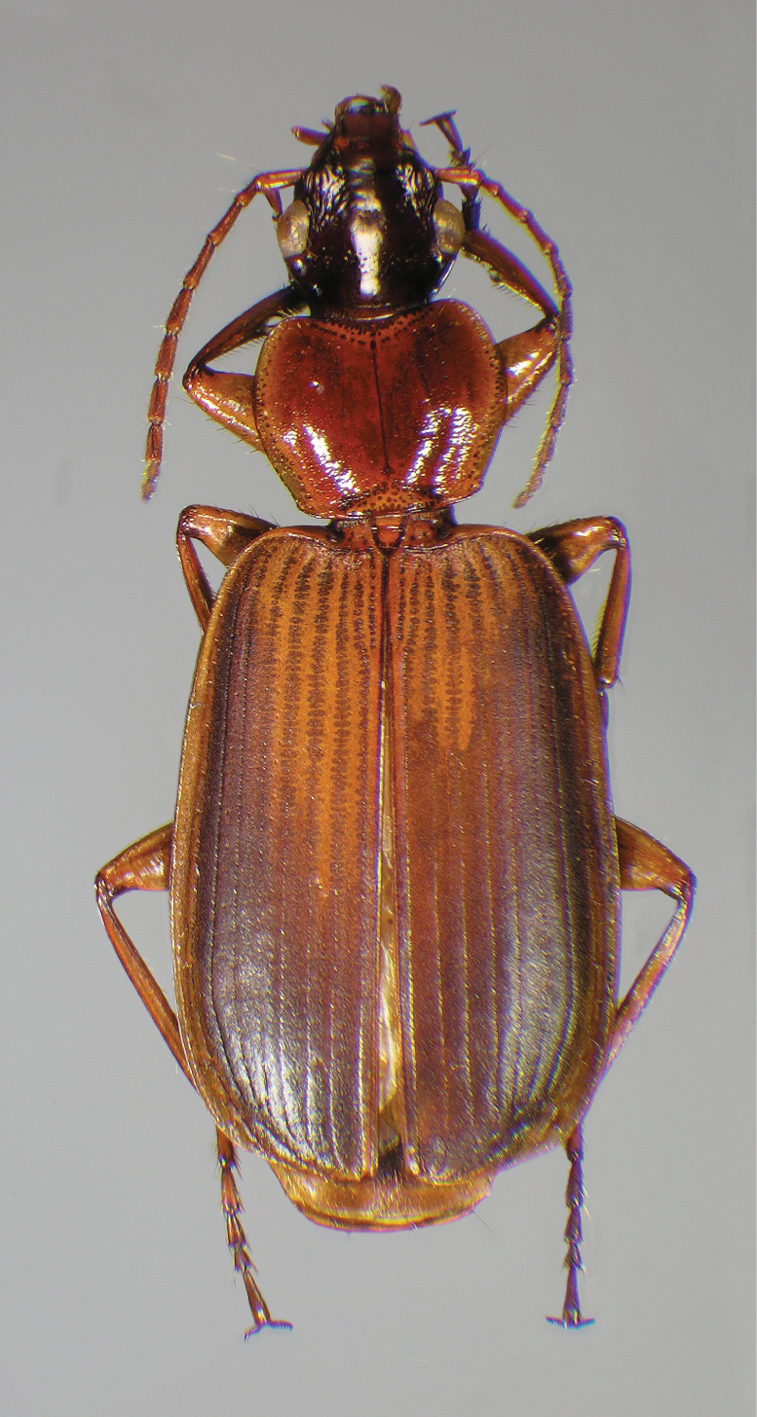
Dorsal habitus and color pattern of *Cymindis complanata* Dejean (OBL 12.50 mm).

Figs 4 [Fig F5] [Fig F6] –7

Cymindis complanata Dejean 1826: 448. Type material (probably holotype, but without a Dejean label) in Chaudoir/Oberthür Collection, [MNHP]. TYPE LOCALITY – St. John’s Bluff, Duval County Florida, U.S.A. ; restricted by Lindroth (1969: 1070) from the original type area, “L’ Amérique septentrionale”. – LeConte 1848:189.
Lebia russata Newman 1840: 31. TYPE female [BMNH]. TYPE LOCALITY. – St. John’s Bluff, Duval County, Florida, U.S.A (Lindroth, 1969: 1070).
Pinacodera complanata (Dejean, 1826); Schaum 1857: 294 [as a junior synonym of *Pinacodera platycollis* Say].
Pinacodera russata Chaudoir 1875: 2 [as a junior synonym of *Pinacodera platicollis* Say]. – Lindroth 1969: 1070.
Pinacodera complanata (Dejean 1826) Chaudoir 1875: 2 [as a junior synonym of *Pinacodera platicollis* Say]. – Casey 1920: 283.– Lindroth 1955: 24. – 1969: 1069–1070. – Ciegler 2000: 119.
Pinacodera platicollis Casey 1920: 283 (not Say). – Lindroth 1969: 1069.

#### Notes about synonymy.

The above synonymy was established by Lindroth (1969), though previously (1955) he treated the names *Pinacodera complanata* Dejean and *Pinacodera russata* Newman as junior synonyms of *Pinacodera limbata* Dejean.

#### Diagnosis.

Adults of *Cymindis complanata* are distinguished from those of other species by ferruginous coloration throughout (Fig. 4) (some slightly lighter in basal third of elytra), by the almost flat elytral intervals, setae extended almost to the constriction of the elytral epipleuron; a noticeable contrast in punctation extending the length of the intervals; scattered arrangements of one to two rows on intervals 3, 5 and 7 and scattered arrangements of two to four rows in intervals 2, 4, 6 and 8 (most specimens have one row of punctures on intervals 3, 5 and 7 and 3 three rows on 2, 4, 6 and 8). In males, the endophallic plate (**ep**) differs as it is almost flat, and apical endophallic lobe (**ael**) is enlarged (Fig. 6A). In females, the form of gonocoxite 2 (**gc2**) differs from all other species apices of ensiform setae extend to and often past gonocoxite 2 apex (Fig. 6B).

#### Description.

With character states of subgenus *Pinacodera* restricted as follows: OBL. 9.5 – 12.5 mm. Length (n= 24 males, 17 females): head 0.88–1.08, pronotum 1.72 – 2.28, elytra 5.33 – 7.17, metepisternum 1.26 – 1.64 mm; width: head 1.62 – 2.16, pronotum 2.16 – 2.92, elytra 3.58 – 5.08, metepisternum 0.62 – 0.80 mm.

*Body proportions*. HW/HL 1.74 – 2.08; PWM/PL 1.18 – 1.33; EL/ EW1.35 – 1.59; ML/MW 1.89 – 2.05.

*Color* (Fig. 4). Dorsum of head entirely ferruginous to rufo-piceous; pronotum ferruginous with lateral margins ranging from translucent to lighter and creamy in appearance; elytra ranging from ferruginous to rufo-piceous, few specimens with basal third of elytra lighter in color, lateral margins translucent to somewhat translucent; antennae and other appendages ferruginous to brown.

*Microsculpture*. Microlines absent from head capsule and pronotum; elytra with mesh pattern isodiametric, microlines distinct.

**Figure 5. F5:**
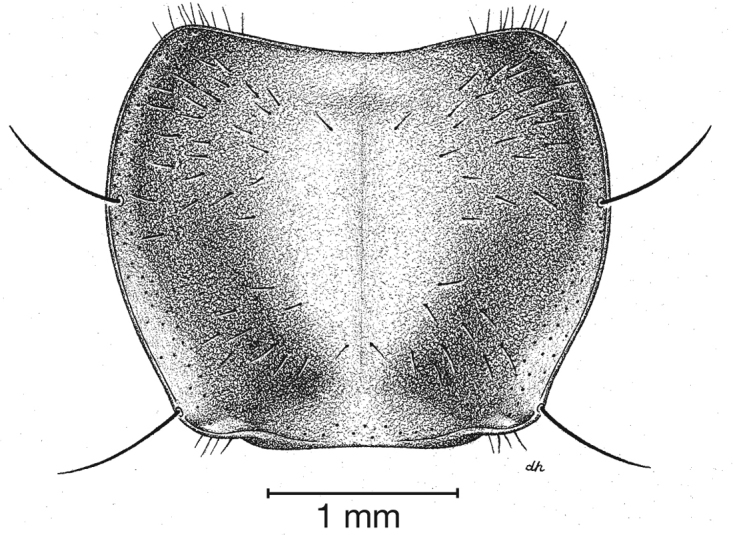
Pronotum, dorsal aspect, of *Cymindis complanata* Dejean.

*Macrosculpture and pilosity*. Dorsal punctures with short setae present, few to many visible on head, pronotum (Fig. 5), and at base of elytra (occasionally few setae extended toward apex of elytra). Elytral epipleuron with setae visible, extended to constriction. Elytral intervals with one to two rows of scattered punctures in intervals 1, 3 and 5 and two to three (rarely four) in intervals 2, 4, 6 and 8.

*Fixed setae*. Pronotum with two setae along each margin; 15–16 lateral (umbilical) setae. Two setae on each of abdominal sterna III to VI, four setae along apical margin of sternum VII (Fig. 3).

*Luster*. Elytra dull.

*Head*. Fine scattered setigerous punctures from hind portion of eye to constriction of neck; also additional fine punctulae from base of neck extended laterally toward clypeus, otherwise glabrous.

*Pronotum*. Disc with setigerous punctures scattered throughout, more densely and evenly so along margins, longest setae along anterior angles (Fig. 5); posterior angles obtuse; posterior margin slightly lobate; basolateral impressions shallow.

*Elytra*. Elytral apices truncate, striae shallowly impressed and punctulate throughout length; intervals flat; a noticeable contrast of punctures extended interval lengths; scattered arrangements of one to two rows on intervals 3, 5 and 7 and scattered arrangements of two to four rows in intervals 2, 4, 6 and 8. Most specimens with a single row of punctures on 3, 5 and 7 and three rows on 2, 4, 6 and 8; epipleuron with setigerous punctures from base to constriction, in a few specimens beyond to apex.

*Hind wings*. Macropterous.

*Legs*. Male tarsi with adhesive vestiture ventrally, two rows of squamo- setae on tarsomeres 1–4 of foreleg and 1–3 of middle leg.

**Figure 6. F6:**
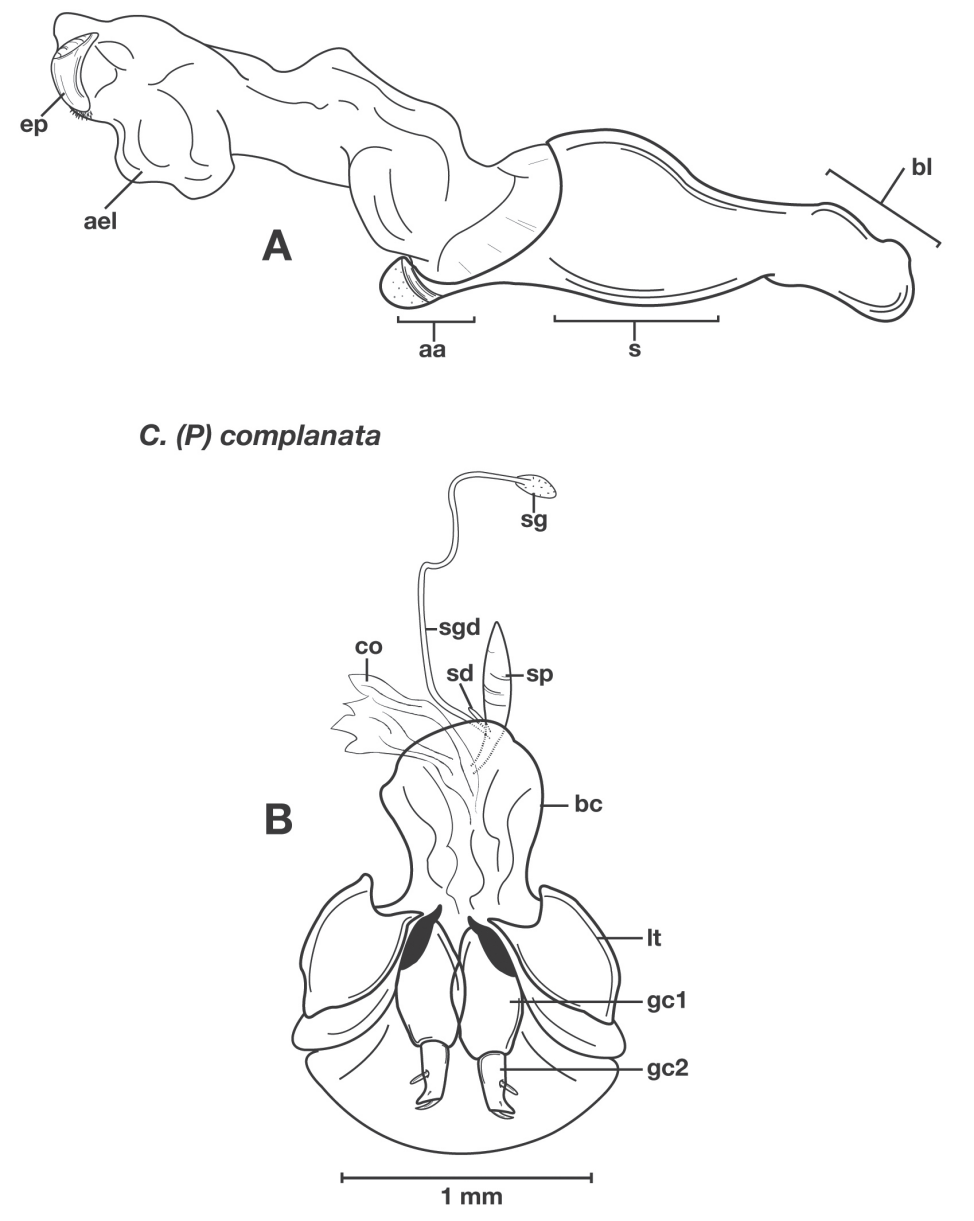
Structural features of *Cymindis complanata* Dejean: **A**, phallus, right lateral aspect, with endophallus everted; **B**, female reproductive tract and ovipositor, ventral aspect. Legend: **aa**, apical area; **ael**, apical endophallic lobe; **bc**, bursacopulatrix; **bl**, basal lobe; **co**, common oviduct; **ep**, endophallic plate; **gc1**, gonocoxite 1; **gc2**, gonocoxite 2; **lt**, lateral tergite; **s**, shaft; **sd**, spermathecal diverticulum; **sg**, spermathecal gland; **sgd**, spermathecal gland duct; **sp**, spermatheca.

*Male genitalia*. (Fig. 6A) Length 1.88 – 2.00 mm. Endophallus with endophallic plate (**ep**) almost flat and apical endophallic lobe (**ael**) enlarged.

*Female genitalia*. Gonocoxite 2 (**gc2**) (Fig. 6B) distinctly, obliquely truncate.

#### Habitat, habits and seasonal occurrence.

The known elevational range of *Cymindis complanata* extends from sea level to 135 m on the eastern slopes of the Appalachian Mountains. All specimens that I have collected (13 in total) were taken from slash pine (*Pinus elliotii* Engelm.) though it has also been recorded from under bark of live loblolly pine (*Pinus taeda* L.). These species of pine are very similar to each other, sharing both reddish-brown bark coloration and an abundance of flaky bark to rest under. As well, they are common within the known range of *Cymindis complanata*. Adults are crepuscular or nocturnal with most activity being observed on tree trunks. Most specimens have been collected in March and April. Methods of collecting include u.v. light, sugaring baits painted on tree trunks, and hand collecting at night.

#### Geographical distribution.

**Figure 7. F7:**
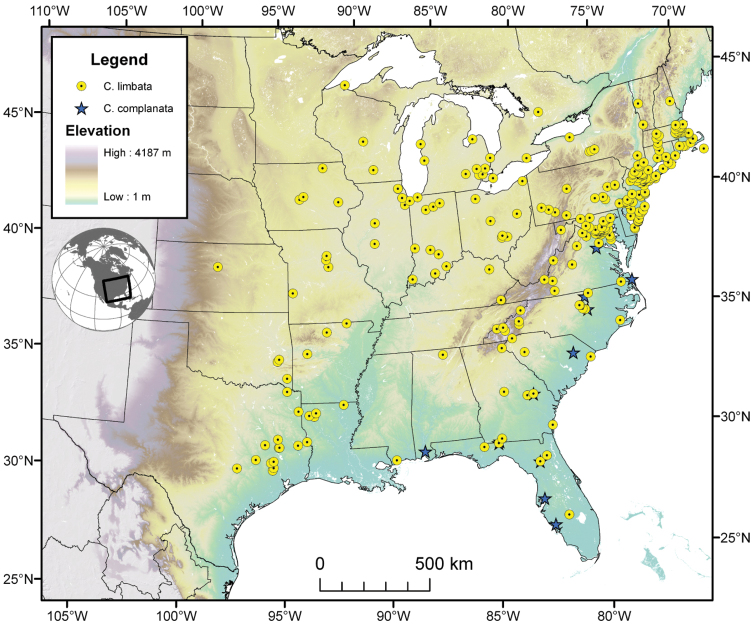
Map of southeastern Canada, U.S.A. and northeastern Mexico, showing position of localities for *Cymindis limbata* Dejean and *Cymindis complanata* Dejean.

The range of this species (Fig. 7) extends in the eastern United States east of the Appalachian Mountains from Maryland south to southern Florida, and westward on the Gulf Coast to western Alabama.

#### Chorological affinities.

*Cymindis complanata* is sympatric in its entire range with *Cymindis limbata* (Fig. 7), sympatric in the northern portion of its range with *Cymindis platicollis platicollis* and in the southern portion of its range with *Cymindis platicollis atripennis*.

#### Material examined.

I have examined 42 specimens of *Cymindis complanata*: 23 males and 19 females. For details see University of Alberta Strickland Virtual Entomology Museum Database (University of Alberta 2009).

### Cymindis (Pinacodera) limbata

Dejean

http://species-id.net/wiki/Cymindis_limbata

Figs 7 [Fig F8] [Fig F9] [Fig F10] –11

Cymindis limbata Dejean 1831: 32, HOLOTYPE in Chaudoir/Oberthür Collection, labeled “ limbata m” and “ Latreille” (Lindroth 1955: 24). TYPE LOCALITY. – Marion, Plymouth County, Massachusetts, U.S.A. (restricted by Lindroth 1969: 1067, from “ l’ Amérique septentrionale”, the original type area). – LeConte 1848: 189.
Pinacodera limbata (Dejean 1831*)*; Schaum 1857: 294. – LeConte 1861: 24. – Chaudoir 1875: 3. – Horn 1881: plate 8, figure 97. – 1882: 147 and 162. – Wickham 1909: 8. – Blatchley 1910: 152. – Leng 1915: 588. – Casey 1920: 279. – Notman 1928: 239. – Brimley 1938: 125. – Fattig 1949: 40. – Lindroth 1955: 24. – 1969: 1067–1068. – Kirk 1969: 16. – 1970: 17. – Ciegler 2000: 119.
Planesus laevigata Motschulsky 1864: 297. – TYPE LOCALITY – Mobile, Mobile County, Alabama, U.S.A.
Pinacodera laevigata (Motschulsky, 1864); Horn 1882: 162. – Casey 1920: 295. – Lindroth 1969; 1067-1068.
Planesus fuscicollis Motschulsky 1864: 298. TYPE AREA. – southern United States.
Pinacodera fuscicollis (Dejean 1831); Horn 1882: 148, 162. – Casey 1920: 285. – Lindroth 1969: 1067.

#### Notes about synonymy.

Lindroth (1969: 1067) stated that the types of *Cymindis laevigata* and *Cymindis fuscicollis* are probably in the Zoological Museum of Moscow University (these types were listed at ZMMU by S. I. Keleinikova 1976). He had not seen them, and questions the synonymy of these names and with *Cymindis limbata* Dejean. I note also that in 1829, some two years before the description of *Cymindis limbata* Dejean, T. W. Harris had correspondence with fellow entomologist N. M. Hentz in which he referred to a *Cymindis* specimen from his collection that had an “ochreous elytral margin, with a humeral lunule of the same color” (Harris 1869). He refers to the specimen as *Cymindis comma*. In 1835, the name *Cymindis comma* appeared again in a list of the insects of Massachusetts that Harris compiled (Hitchcock 1835). I believe it likely that Harris was referring to what is now known as *Cymindis limbata* but because the 1829 correspondence was not published until 1869, and was not a formal description, the name *Cymindis comma* is not valid.

#### Diagnosis.

**Figure 8. F8:**
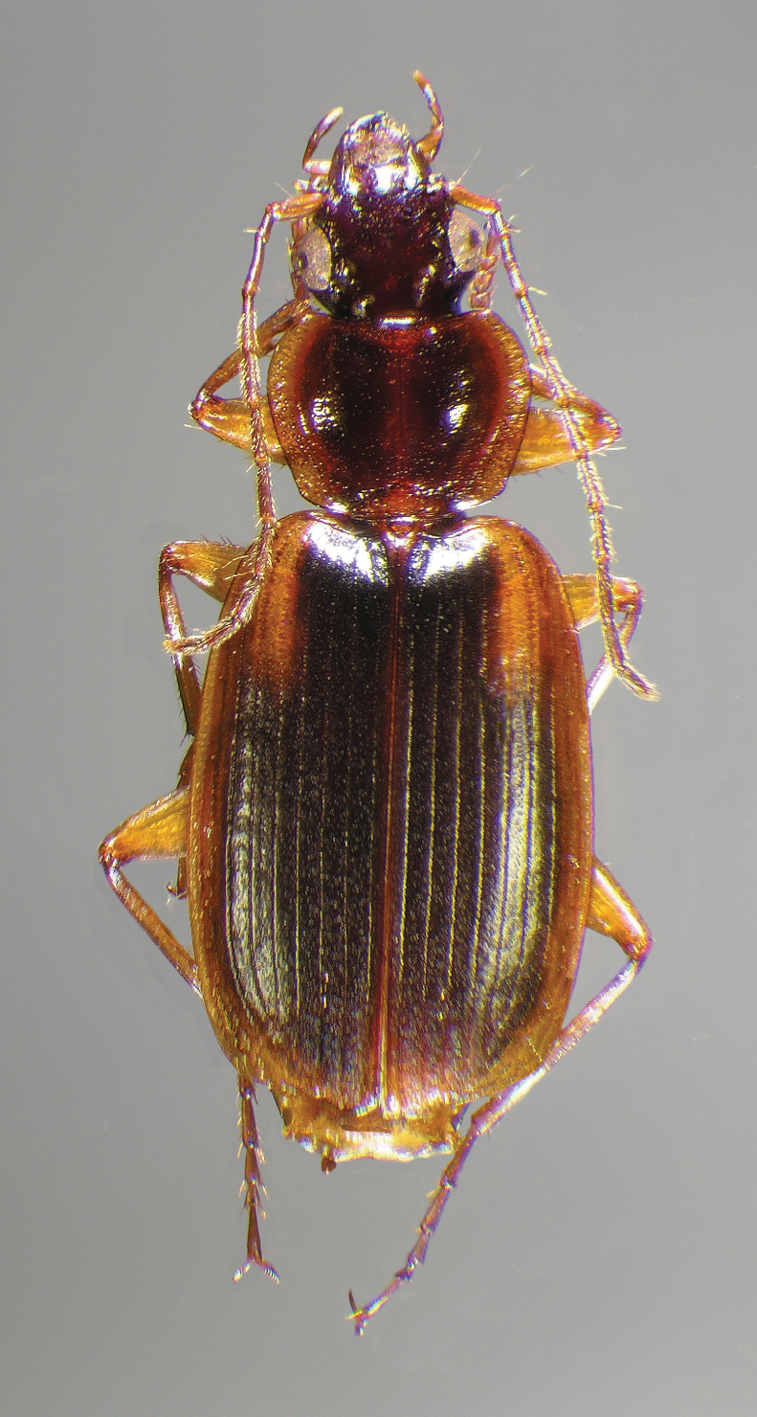
Dorsal habitus and color pattern of *Cymindis limbata* Dejean (OBL 9.83 mm).

Adults of *Cymindis limbata* are distinguished from those of other species by: a pale, testaceous humeral macula (Fig. 8) extended from interval 6 (rarely from interval 5) to the outer margin and posteriorly as far as one quarter (0.25) the length of the elytra; pronotum broadly rounded; antennomere 8, 3.0–3.9 × longer than wide. In males, genitalia with endophallus having a distinct sclerotized patch (Fig. 10A, 11A) medially, phallus apex with distinct curvature to the left when viewed from dorsal aspect (Fig. 11C); phallus apex broadly pointed and distinctly shaped (Fig. 10A, 11B). In females, gonocoxite 2 (**gc2**) long and narrow, sharply pointed at apex; apical ensiform setae curved outward and extended almost to gonocoxite apices (Fig. 10B).

#### Description.

With character states of subgenus *Pinacodera* restricted as follows: OBL. 8.33 – 10.92 mm. Length (n= 20 males, 20 females): head 0.72 – 0.96, pronotum 1.52 – 2.16, elytra 4.62 – 6.41 mm; width: head 1.48– 2.00, pronotum 2.00 – 2.88, elytra 3.21 – 4.75 mm.

*Body proportions*. HW/HL 1.78 – 2.18; PWM/PL 1.26 –1.46; EL/EW 1.35 –1.52.

*Metepisternum*. Individuals show proportions of a minimum of 1.73× as long as wide.

*Hind wings*. Macropterous.

*Color*. Dorsum of head brunneous to rufo-piceous; pronotum brunneous to rufo-piceous on disc, margins sometimes in some specimens lighter; dorsum of elytra brunneo-piceous, margins somewhat paler, testaceous humeral macula extended from interval 6 (rarely interval 5) to the outer margin, and as far as one quarter the length of the elytra; antennae brunneo-testaceous to brunneous; palpi brunneo-testaceous to brunneo-piceous; epipleura testaceous to brunneo-testaceous; thoracic sclerites and abdominal sterna testaceous to piceous (apical edge of abdominal sterna in many specimens darker than basal edge).

*Microsculpture*. Microlines not visible on dorsum of head capsule and pronotum at 50× magnification. Elytra with mesh pattern isodiametric, microlines clearly defined throughout dorsal surface.

*Macrosculpture and pilosity*. Head capsule with very fine, randomly scattered setigerous punctures on dorsal surface (setae not visible or only barely so at 50× magnification) from constriction of neck extended anteriorly toward clypeus. Elytra with striae moderately impressed and punctulate throughout length; intervals slightly convex (few with greater convexity in intervals 1, 3 and 5); one or two (most specimens one) irregular rows of fine punctures extended length of intervals 1, 3 and 5; two or three (most specimens two) irregular rows of fine punctures extended the length of intervals 2, 4 and 6; interval 8 with two to four rows of fine punctules extended interval length. Abdominal sterna with fine pilose punctures throughout.

*Fixed setae*. Pronotum with two setae along each margin. Elytra with 15 or 16 lateral (umbilical) setae; two setae on each of abdominal sterna III to VI; four setae along apical margin of sternum VII (Fig. 3).

*Luster*. Elytra glossy

*Head*. Eyes, labrum, labium, palpi, typical for Cymindidina.

**Figure 9. F9:**
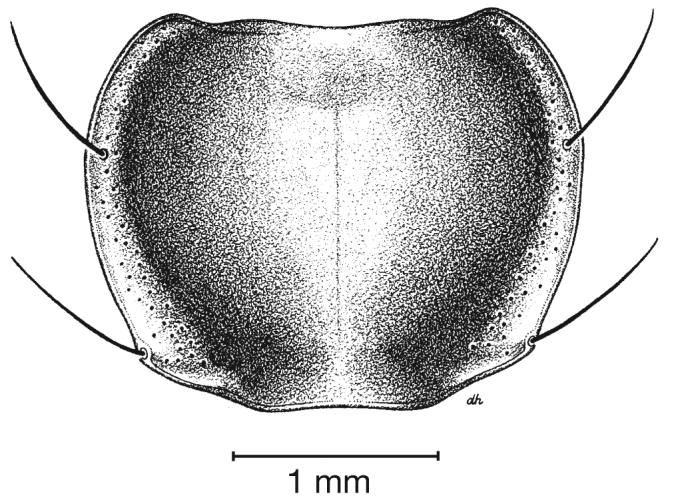
Pronotum, dorsal aspect, of *Cymindis limbata* Dejean.

*Pronotum*. Anterior transverse impression shallow (Fig. 9); posterior transverse impression moderately deep; median longitudinal impression moderately shallow, posteriolateral angles obtuse to almost rounded.

*Elytra*. Humeri broadly rounded; striae moderately impressed; lateral margin smooth, rounded and widened preapically; apex truncate (Fig. 8).

*Legs*. Males with adhesive vestiture ventrally, two rows of squamo- setae on tarsomeres 1–4 of foreleg and 1–3 of middle leg.

*Male genitalia*. Phallus apex curved to left when viewed from dorsal aspect (Fig. 11C), apex pointed in lateral aspect (Fig. 11B). Ventral and dorsal surface of apical area slightly dimpled (Fig. 11B) (most specimens) or not, few specimens with vertical striations (absent from most specimens) extended from mid length of apical area to apex of phallic shaft (**s**). Endophallus with microtrichial patch (**mp**) at midlength of the endophallus sac. Endophallus with a curved endophallic plate (**ep**) (Lindroth 1969: 1080–1081) apically when viewed ventrally in everted condition.

**Figure 10. F10:**
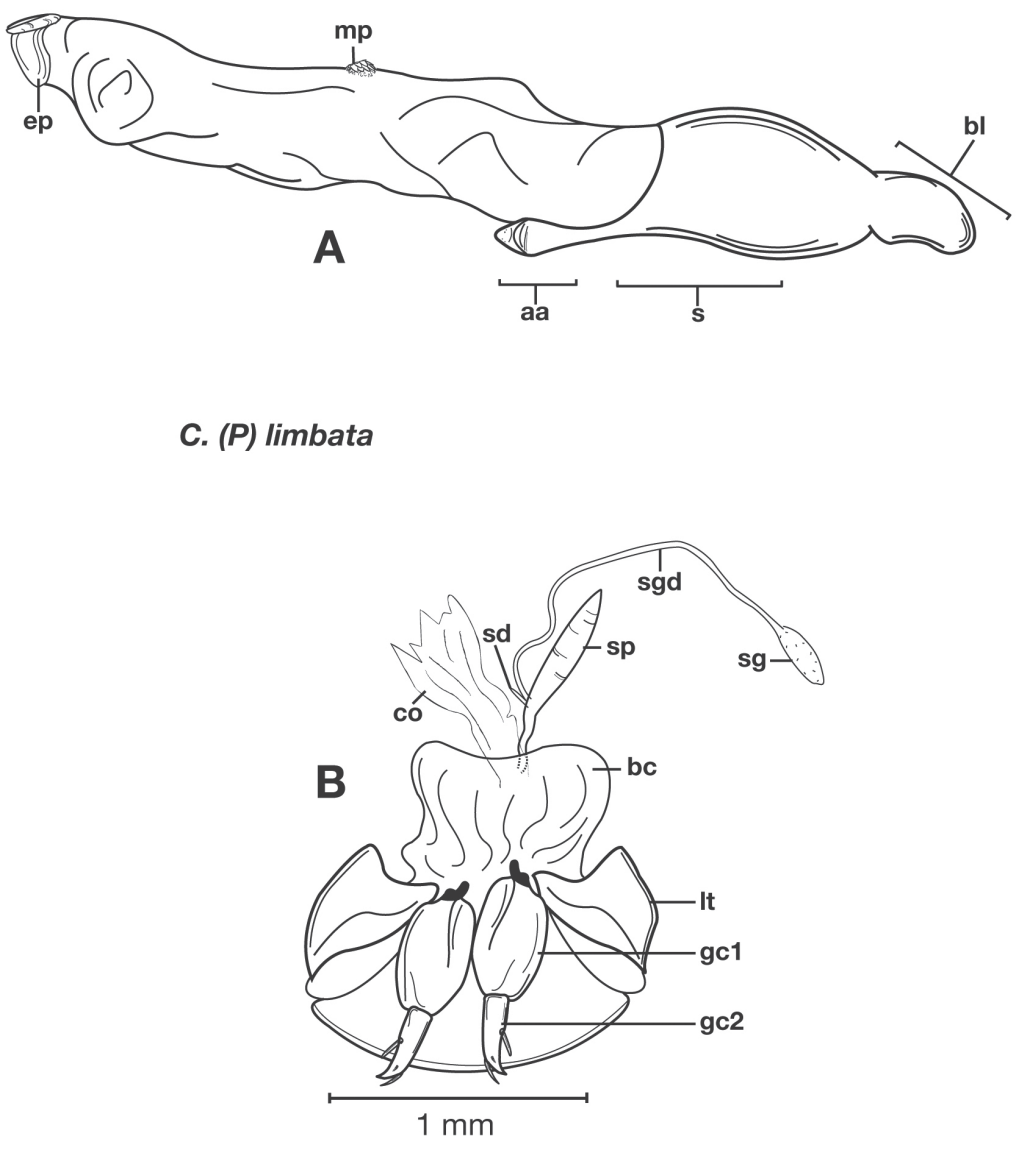
Structural features of *Cymindis limbata* Dejean: **A**, phallus and everted endophallus, right lateral aspect; **B**, female reproductive tract and ovipositor, ventral aspect. Legend: **aa**, apical area; **bc**, bursa copulatrix; **bl**, basal lobe; **co**, common oviduct; **ep**, endophallic plate; **gc1**, gonocoxite 1; **gc2**, gonocoxite 2; **lt**, lateral tergite; **mp**, microtrichial patch **s**, shaft; **sd**, spermathecal diverticulum; **sg**,spermathecal gland; **sgd,** spermathecal gland duct; **sp**, spermatheca.

*Female genitalia*. Gonocoxite 2 (**gc2**) long and narrow (Fig. 10B), sharply pointed at apex, curved outward; apical ensiform setae curved out slightly and extended almost to apex. Internal genitalia with long cylindrical spermatheca (**sp**), moderately long associated spermathecal gland (**sg**), and moderately long spermathecal diverticulum (**sd**) located at base of spermathecal gland duct (**sgd**).

**Figure 11. F11:**
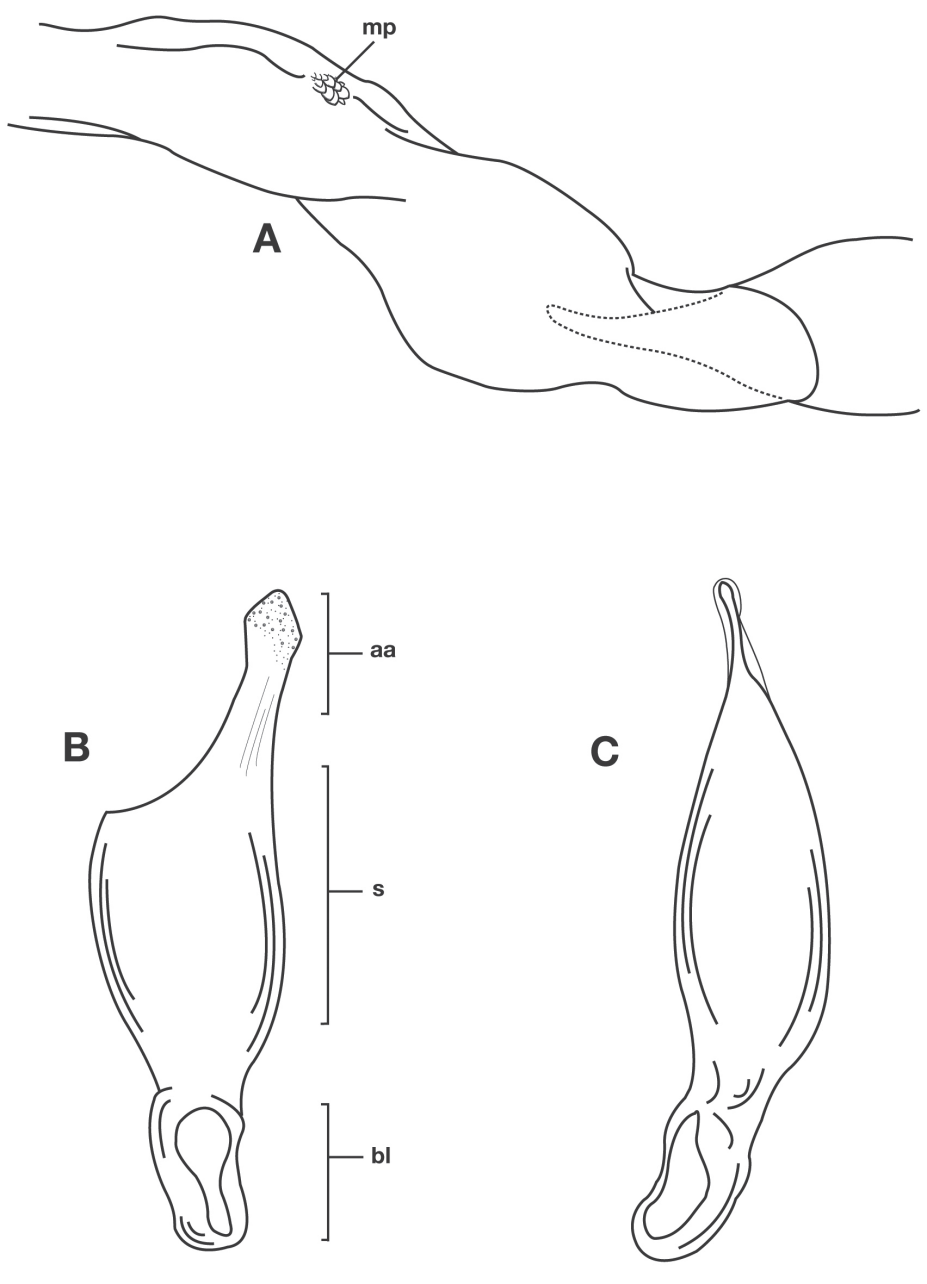
Male genitalia of *Cymindis limbata* Dejean: **A**, phallus, apical portion, and everted endophallus, ventral aspect; **B**, lateral aspect of phallus (endophallus inverted); **C**, dorsal aspect, showing curvature of phallic apex to left. Legend: **aa**, apical area; **bl**, basal lobe; **mp**, microtrichial patch; **s**, shaft.

#### Habitat, habits and seasonal occurrence.

The known elevational range of *Cymindis limbata* extends from sea level to 1935 m. Specimens have been collected on and under bark, in leaf litter and from under stones in forests of oak, pine, tamarack, aspen, beech-magnolia and maple. Specimens have been found near creek and pond margins, among beach wash-up and have also been collected from squirrel nests in trees. Adults are crepuscular or nocturnal with most activity being observed on tree trunks. Adults are most commonly collected from May to August. Most teneral adults were found from late June to early July, suggesting emergence from pupal case also occurs around this time.

Methods of collecting include asafetida and molasses bait traps, sugaring baits painted on tree trunks, beating and sweeping vegetation, at incandescent light and u.v. light, Malaise traps, Lindgren funnels, Berlese traps, flight intercept traps (FIT’s), pitfall traps, and hand collecting.

#### Geographical distribution.

The range of this species (Fig. 7) extends in eastern Canada from southern Quebec west to southern Ontario, and in the eastern United States east of the Appalachian Mountains from Maine south to southern Florida, west to eastern Colorado and Nebraska, and south to southern Texas.

#### Chorological affinities.

*Cymindis limbata* is sympatric in the northern and western portion of its range with *Cymindis platicollis platicollis*, in the southeastern portion of its range with *Cymindis platicollis atripennis* and *Cymindis rufostigma*, and in the central portion of its range with *Cymindis complanata*.

#### Fossil specimens.

Fossil packrat (*Neotoma* sp.) middens have been used to determine late Quaternary insect assemblages in the Chihuahuan desert areas of northern Mexico and southwestern Texas (Elias 1992, Elias and Van Devender 1992, Elias et al. 1995). Particularily relevant to this treatment are middens examined from the Trans Pecos and Bolson de Mapimi areas where both *Cymindis limbata* and *Cymindis platicollis* subfossils had been reported (Elias 1992). These species are primarily warm-temperate deciduous forest-adapted eastern species and currently both have southwestern range limits that do not extend to the Trans Pecos and Bolsom de Mapimi areas. Unfortunately, the specimens were unavailable for reexamination but in their place Scott Elias graciously sent me two S.E.M. images (a pronotum and a single elytron) of the putative *Cymindis limbata* fossils.

I was able to determine that neither fossil specimen was *Cymindis limbata*, based on examination of these images. First, The fossil image of the pronotum has narrowly bordered pronotal margins and hind angles that are almost right-angled. This contrasts with the pronotum of *Cymindis limbata*, whichtypically has a widely explanate margin and hind angles that are rounded, or at least widely obtuse. As well, I have not observed *Cymindis limbata* with deep punctures either side of the median longitudinal impression of the pronotum, a feature characteristic of the fossil specimen. The S.E.M. specimen appeared to be a lebiomorph carabid and I thought it to be a member of the genus *Calleida* so I sent the information to carabidologist Achille Casale (University of Sassari, Italy) who has detailed knowledge of the Mexican *Calleida* fauna. He confirmed that the pronotum was “*Calleida*-like” and appeared not to belong to *Pinacodera* (Casale 2008, personal communication).

The S.E.M. of the single fossil elytron also differed from that of *Cymindis limbata*. The width of the fossil elytron was more than 5 mm whereas *Cymindis limbata* elytra range from 3.2 to 4.75 mm. Additionally, *Cymindis limbata* typically have more than one row of shallow and fine punctures in the even elytral intervals and more than one row in interval 8. The fossil specimen has a single row in all intervals, which are obviously deeper and farther apart than observed in *Cymindis limbata*. After considering these discrepancies I believe it more likely that the elytron belongs to *Cymindis punctigera punctigera* LeConte as it is similar in size and morphological characteristics, and was found within the limits of the current geographical range of *Cymindis punctigera punctigera*. *Cymindis punctigera punctigera* has been collected in the recent past from nests of packrats of the genus *Neotoma* (Say and Ord 1825).

#### Material examined.

I have examined 1001 specimens of *Cymindis limbata*: 432 males and 569 females. For details see University of Alberta Strickland Virtual Entomology Museum Database (University of Alberta 2009).

### Cymindis (Pinacodera) platicollis

Say

http://species-id.net/wiki/Cymindis_platicollis

**Figure 12. F12:**
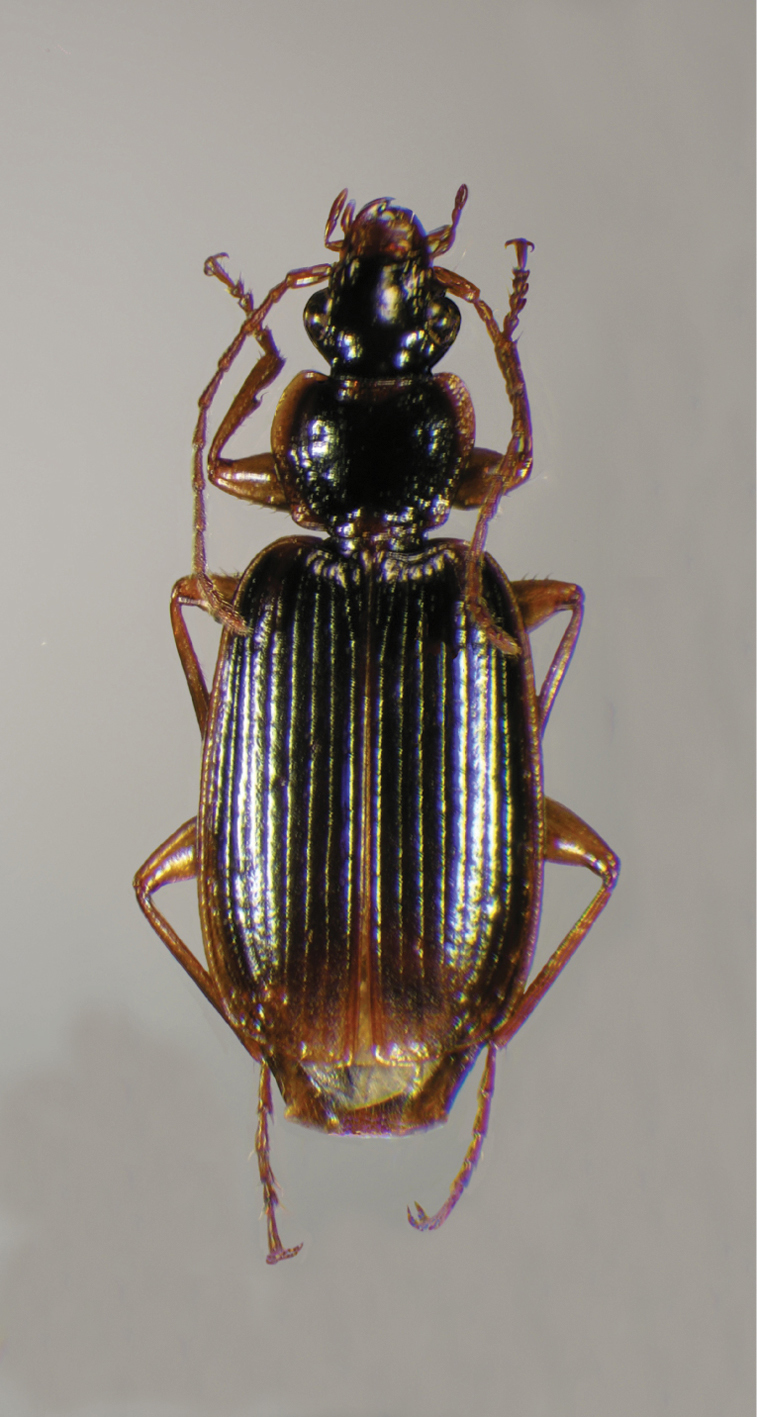
Dorsal habitus and color pattern of *Cymindis platicollis platicollis* (Say) (OBL 9.16 mm).

Figs 12 [Fig F13] [Fig F14] [Fig F15] [Fig F16] –17

#### Remarks.

This is a polytypic species that includes two subspecies (*Cymindis platicollis platicollis* (Say) and *Cymindis platicollis atripennis* (Casey)).

#### Diagnosis.

Adults of *Cymindis platicollis* are distinguished from those of other species in the *limbata* species group through the following unique combination of external and genitalic characters: translucently bordered pronotum and elytra (Fig. 12, 16, 19B); evenly brunneo-piceous to rufo- piceous elytral disc coloration and interval 8 with two to four rows of scattered setigerous punctures; apical area of phallus dimpled (at least at apex) on both dorsal and ventral surfaces (Fig. 14A, 15A-B); longitudinal striations absent.

#### Description.

OBL 8.17 – 11.67 mm.

*Color*. Dorsum of head brunneous or rufous to rufo-piceous; dorsum of pronotum and elytra brunneous or rufous to rufo-piceous with pale, somewhat translucent margins; antennae rufo-testaceous to brunneous; palpi rufo-testaceous to brunneous; epipleura testaceous to rufo-testaceous; lateral and ventral thoracic sclerites and abdominal sterna testaceous to piceous.

*Microsculpture*. Individuals with microlines not visible (or hardly so) on dorsum of head capsule and pronotum at 50× magnification. Elytra with mesh pattern isodiametric, microlines clearly defined.

*Macrosculpture and pilosity*. Head capsule dorsally with very fine to coarse scattered setigerous punctures on dorsal surface from constriction of neck extended anteriorly toward clypeus, ventrally with scattered pilose punctures laterally, extended from constriction of neck to gula. Pronotum with shallow to somewhat deep and randomly spaced setigerous punctures, setal length very short to moderate at 50×; ventrally with randomly spaced setigerous punctures extended from margin of proepipleuron to apex of intercoxal process; setae visible on prosternum but not proepisterna. Elytra with striae shallowly to moderately deeply impressed, punctulate throughout length; intervals flat to slightly convex; few specimens with odd intervals somewhat raised and even intervals flat. Other details in “Variation” section below.

*Fixed setae*. Pronotum with two setae along each lateral margin. Elytra with 14–16 lateral (umbilical) setae;two setae on each of abdominal sterna III to VI; four setae along apical margin of sternum VII (Fig. 3).

*Luster*. Elytra glossy.

*Pronotum*. (Fig. 19B) Anterior and posterior impression shallow; median longitudinal impression moderately shallow; posteriolateral angles widely obtuse (approaching round); posterior margin slightly lobate.

*Head*. Eyes, labrum, labium, and palpi, typical for Cymindidina.

*Elytra*. Humeri broadly rounded; striae shallowly to moderately impressed; lateral margin smooth, rounded and widened preapically; elytral apices subtruncate (Fig. 12, 16).

*Hind wings*. Macropterous.

*Legs*. Males with adhesive vestiture ventrally, two rows of squamo- setae on tarsomeres 1–4 of foreleg and 1–3 of middle leg.

*Male genitalia* (Fig. 14A, 20A). Endophallus with ventral surface slightly curved. Ventral and dorsal surface of apical area somewhat to markedly dimpled in appearance (Fig. 15A-B). Endophallus with a slightly curved endophallic plate (**ep**) apically (Lindroth 1969: 1080–1081), when viewed ventrally in everted condition (Fig. 14A).

*Female genitalia*. Gonocoxite 2 (**gc2**) (Fig. 14B) moderately long and narrow, slightly to moderately curved outwards. Internal genitalia with long cylindrical spermatheca (**sp**), moderately long associated spermathecal gland (**sg**), and moderately long spermathecal diverticulum (**sd**) located at base of spermathecal gland duct (**sgd**).

#### Geographical distribution.

The range of this species (Fig. 17) extends in eastern Canada from southern Quebec west to southern Ontario; in eastern United States from Maine south to southern Florida, west to eastern Colorado and Nebraska, and south to southern Texas. In Mexico it is known from Nuevo Leon in the northern portion of the Sierra Madre Oriental.

#### Chorological affinities.

*Cymindis platicollis* is sympatric in portions of its range with *Cymindis limbata*, *Cymindis complanata*, *Cymindis rufostigma* and marginally with *Cymindis punctigera punctigera*, and *Cymindis chevrolati*.

### Cymindis platicollis platicollis

(Say) stat. n.

http://species-id.net/wiki/Cymindis_platicollis_platicollis

Figs 12 [Fig F13] [Fig F14] –15 , 17

Lebia platicollis Say 1823: 14. NEOTYPE male; TYPE LOCALITY – Allegheny, Allegheny County, Pennsylvania, U.S.A. ; designated by Lindroth and Freitag 1969: 350.
Cymindis platicollis ; LeConte 1848: 189.
Cymindis fuscata Dejean 1831: 321. HOLOTYPE (probably), male in Chaudoir/Oberthür collection, labeled: “Latreille” [handwritten– green paper] [MNHP] – LeConte 1848: 189.
Pinacodera fuscata ; Schaum 1857: 294. – Chaudoir 1875: 3. – Horn 1882: 162. – Leng 1915: 588. – Casey 1920: 280. – Notman 1928: 240. – Lindroth 1955: 24. – 1969: 1068. – Kirk 1969: 16. – 1970: 17.
Pinacodera platycollis ; Schaum 1857: 294 (unjustified emendation) – Brimley 1938:125.
Pinacodera platicollis ; Chaudoir 1875: 2. – Horn 1882: 147. – Blatchley 1910: 152, 153. – Notman, 1928: 239. – Lindroth, 1969: 1068–1069. – Lindroth and Freitag 1969: 350. – Kirk 1969: 16. – 1970: 17. – Ciegler 2000: 119.
Pinacodera punctigera (Dejean 1831) Wickham 1897: 112 [not LeConte 1851: 178].
Cymindis planipennis ; Casey 1913: 189 [not LeConte 1863: 6].
Pinacodera abbreviata Casey 1920: 283. HOLOTYPE male labeled: “Col”.; “Casey bequest 1925”; “TYPE USNM 47614” [ red paper], “abbreviata Csy” [handwritten] [USNM]. TYPE AREA – Colorado, U.S.A. syn. n.
Pinacodera obscura Casey 1920: 284. Female, labeled: “Southern Pines, A H Manee”, N.C.; “Casey bequest, 1975”; “TYPE USNM 47612” [red paper]; “*obscura* Csy” [handwritten] [USNM]. – Brimley 1938: 125. – Fattig 1949: 40. syn. n.
Pinacodera ampliata Casey 1920: 282. [= *Pinacodera planipennis*Casey 1913].

#### Holotype

female, labeled: “Col”; “Casey bequest 1925”; “TYPE USNM 47611” [red paper]; “ampliata Csy” [handwritten] [USNM].

#### Diagnosis.

Specimens of this subspecies have uniformly colored head, pronotum, and elytra, with translucently bordered pronotum and elytra (Fig. 12).

#### Description.

With character states of subgenus *Pinacodera* and species *Cymindis platicollis* restricted as follows: OBL. 8.17 – 11.67 mm. Length (n= 20 males, 20 females): head 0.80 – 1.08, pronotum 1.56 – 2.36, elytra 4.92 – 6.83, metepisternum 1.10 – 1.70 mm; width: head 1.60 – 2.28, pronotum 2.00 – 3.32, elytra 3.33 – 5.17, metepisternum 0.60 – 0.84 mm.

*Body proportions*. HW/HL 1.83 – 2.26; PWM/PL 1.16 – 1.41; EL/EW 1.29- 1.49; ML/MW 1.71 – 2.07.

*Color*. Dorsal surface of head brown to rufo-piceous; pronotum and elytra brunneo-piceous to rufo-piceous, with pale, somewhat translucent margins. Antennae and palpi rufo-testaceous to brunneous palpi; elytral epipleura testaceous to rufo-testaceous; ventral thoracic sclerites and abdominal sterna testaceous to piceous.

*Microsculpture*. Microlines not visible on dorsum of head capsule and pronotum at 50× magnification. Elytra with mesh pattern isodiametric, microlines clearly defined throughout dorsal surface.

*Macrosculpture and pilosity*. Head capsule dorsally with fine, randomly scattered setigerous punctures (setae not visible or only barely so at 50× magnification) from constriction of neck extended anteriorly toward clypeus. Elytra with striae moderately impressed and punctulate throughout length; intervals slightly convex (few with greater convexity in intervals 1, 3 and 5); abdominal sterna with fine pilose punctures throughout.

*Fixed setae*. Elytra with 14–15 lateral (umbilical) setae;two setae on each of abdominal sterna III to VI; 4 setae along apical margin of sternum VII (Fig. 3).

*Luster*. Head capsule and pronotum glossyl elytra moderately glossy.

**Figure 13. F13:**
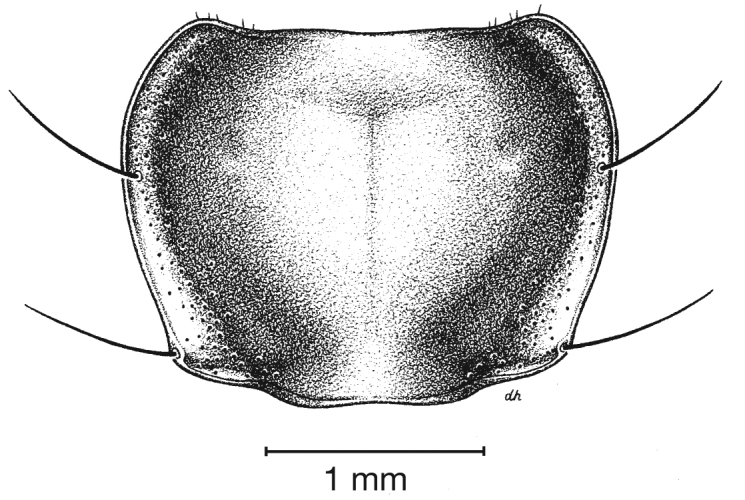
Pronotum, dorsal aspect, of *Cymindis platicollis platicollis* (Say).

*Pronotum*. Anterior transverse impression shallow (Fig. 13); posterior transverse impression moderately deep; median longitudinal impression moderately shallow; posteriolateral angles almost right angled to obtuse.

*Hind wings*. Macropterous.

**Figure 14. F14:**
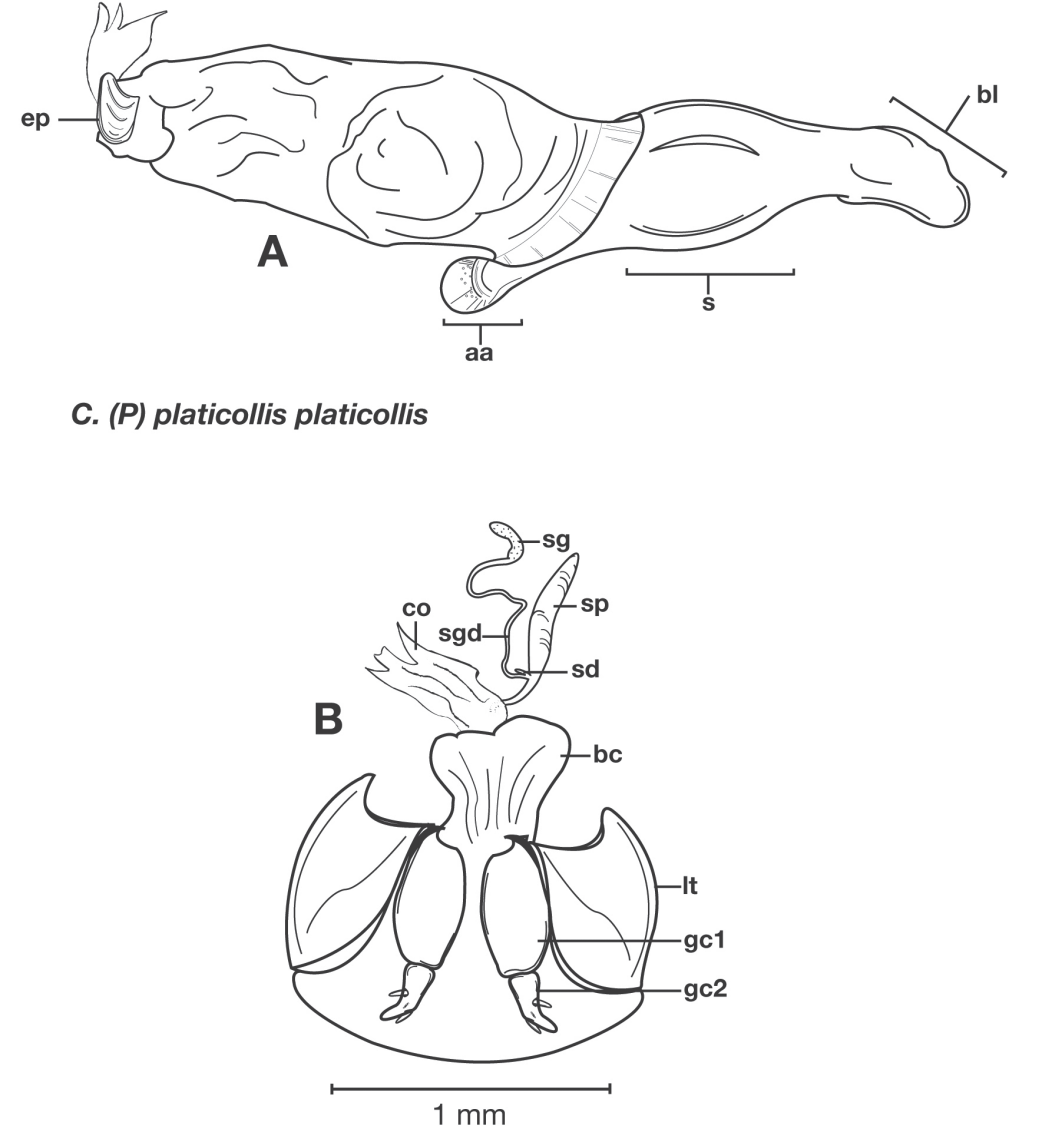
Structural features of *Cymindis platicollis platicollis* (Say): **A**, phallus and everted endophallus, right lateral aspect; **B**, female reproductive tract and ovipositor, ventral aspect. Legend: **aa**, apical area; **bc**, bursa copulatrix; **bl**, basal lobe; **co**, common oviduct; **ep**, endophallic plate; **gc1**, gonocoxite 1; **gc2**, gonocoxite 2; **lt**, lateral tergite; **s**, shaft; **sd**, spermathecal diverticulum; **sg**, spermathecal gland; **sgd**, spermathecal gland duct; **sp**, spermatheca.

*Male genitalia*. Phallus (Fig. 14A) length 1.70 – 2.42 mm.

#### Variation.

**Figure 15. F15:**
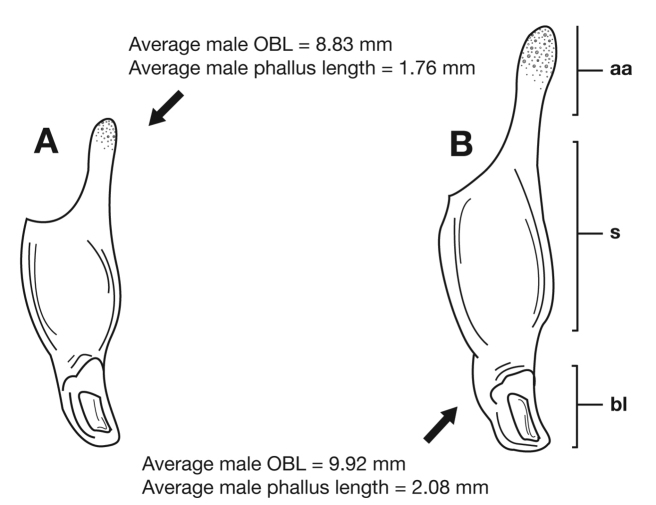
Lateral aspect of phallus (sac everted) of *Cymindis platicollis platicollis* (Say), showing interpopulation variation of phallic apex texturing and difference in typical phallus size between northeastern (**A**) and southwestern (**B**) populations. Legend: **aa**, apical area; **bl**, basal lobe; **s**, shaft; **obl**, overall body length.

Through the range of *Cymindis platicollis platicollis* a three-phased cline is observed (Fig. 17, Table 4). **Phase 1:** northeastern specimens have an average overall body length of 8.83 mm, average phallus length of 1.76 mm (Fig. 15A) and are dorsally glabrous; (~75%) with a single row of ~50–80 (interval 2 with more than 70) punctures in interval 1–7, some (~25%) with one to two rows of punctures in intervals 2, 4 and 6, all others with one row of punctures (except interval 8 with two to four rows). **Phase 2:** more southern specimens have an average overall body length of 9.22 mm, average phallus length 1.82 mm and have dorsal setation on humeral area of elytra; some specimens with few setae visible on dorsum of head and disc of elytra; (~86 %) with odd intervals bearing one row of scattered setigerous puncture and even intervals bearing two or three rows of scattered setigerous punctures, others (~14%) with interval 2 having two or three rows of setigerous punctures (rarely one row), interval 8 with two or three rows, and all other intervals bearing one row of setigerous punctures. **Phase 3:** southwestern specimens (extreme south west Oklahoma to mid-western Texas south west to Nuevo Leon) have an average overall body length of 9.92 mm, an average phallus length of 2.08 mm (Fig. 15B) and are dorsally setose, most individuals having one or two rows (mostly two) of moderately deep, randomly spaced, pilose punctures in all intervals, interval 8 with two to three rows. Few with various combinations of above.

**Table 4.** Geographical variation in the extent of elytral punctation in *Cymindis platicollis platicollis* (Say), by state (and regions of Texas) and number of individuals examined. Legend: **1**, dorsal surface of elytra glabrous, punctation fine; **2**, dorsal surface of elytra setose basally, punctation moderately fine; **3**, entire dorsal surface of elytra setose, punctation coarse.

**Locality**	**N**	**Punctation states / No. individuals**		
		**1**	**2**	**3**
Nebraska	6	6	0	0
Iowa	8	8	0	0
Kansas	2	1	1	0
Arkansas	2	2	0	0
Mississippi	31	31	0	0
Louisiana	16	13	3	0
Oklahoma	66	1	61	4
East Texas	45	10	35	0
Mid Texas	74	1	21	52
West texas	16	4	0	12
New Mexico	1	0	0	1
Nuevo Leon	3	0	0	3
Totals	270	77	121	72

#### Habitat, habits and seasonal occurrence.

The known elevational range of *Cymindis platicollis platicollis* extends from 3 to 1935 m. Specimens were collected under stones, under and on bark of trees and associated mosses in forests of buckeye, beech-magnolia, elm, hackberry, hickory, juniper, mesquite, oak, oak-pine, and tamarack. It has been collected from shrub species *Leucaena pulver* (Schltdl.), from bromeliads associated with oak and also from the nest of woodrats, *Neotoma micropus* Baird. Adults are crepuscular, and most commonly collected from late February through July.

I witnessed several pairs in copula over a three-week period of collecting in Georgia and Florida from late February to mid-March of 2008. Of these mated pairs I brought 9 females and 10 males back to the University of Alberta to attempt rearing larvae. I kept them all in a single plastic container with substrate from under the trees they were captured on. A wet ball of tissue provided moisture, and several 3rd to 5th instar larvae of cabbage looper moth species *Trichoplusia ni* (Hübner) were introduced each week for food. All individuals (with the exception of one female) survived for the first three months in captivity. By mid-June (three months after capture) males started to die, and within the next two weeks all had expired. All of the remaining eight females lived at least until mid-September (6 months after capture) with the last individual dying in early November (7.5 months after capture). Males lived an average of 82 days after capture and females lived more than twice as long with an average lifespan of 166.5 days after capture.

All beetles were removed from the container every week and the substrate was searched for eggs and larvae. No evidence of oviposition was found. Other attempts to rear larvae from mated *Pinacodera* adults (Mahar, 1978) were also unsuccessful. Many lebiines are known to have unusual ovipositional habits or needs; that may also be the case in *Pinacodera* and a reason why rearing is problematic.

Collecting methods include asafetida and molasses traps, sugar baits painted on tree trunks, beating and sweeping vegetation, at light and u.v. light, Lindgren funnel traps, Berlese traps, Malaise traps, flight intercept traps (FIT’s), pitfall traps, hand collecting, and sticky traps.

#### Geographical distribution.

The range of this subspecies (Fig. 17) extends in eastern Canada from southern Quebec west to southern Ontario; in the eastern United States from Maine south to mid-Georgia west to eastern Colorado and Nebraska south to southern Texas. In Mexico it is known from Nuevo Leon in the northern portion of the Sierra Madre Oriental.

#### Evolutionary affinities.

This subspecies is, by definition the closest relative of *Cymindis platicollis atripennis*.

#### Chorological affinities.

*Cymindis platicollis platicollis* is sympatric in portions of its range with *Cymindis limbata*, *Cymindis complanata*, *Cymindis punctigera punctigera*, and *Cymindis chevrolati*. It is allopatric with *Cymindis platicollis atripennis*, *Cymindis rufostigma*, and all other taxa in the *limbata* species group.

#### Material examined.

I have examined 897 specimens of *Cymindis platicollis platicollis*: 24 males and 17 females were dissected. For details see University of Alberta Strickland Virtual Entomology Museum Database (University of Alberta 2009).

### Cymindis platicollis atripennis

(Casey) stat. n.

http://species-id.net/wiki/Cymindis_platicollis_atripennis

**Figure 16. F16:**
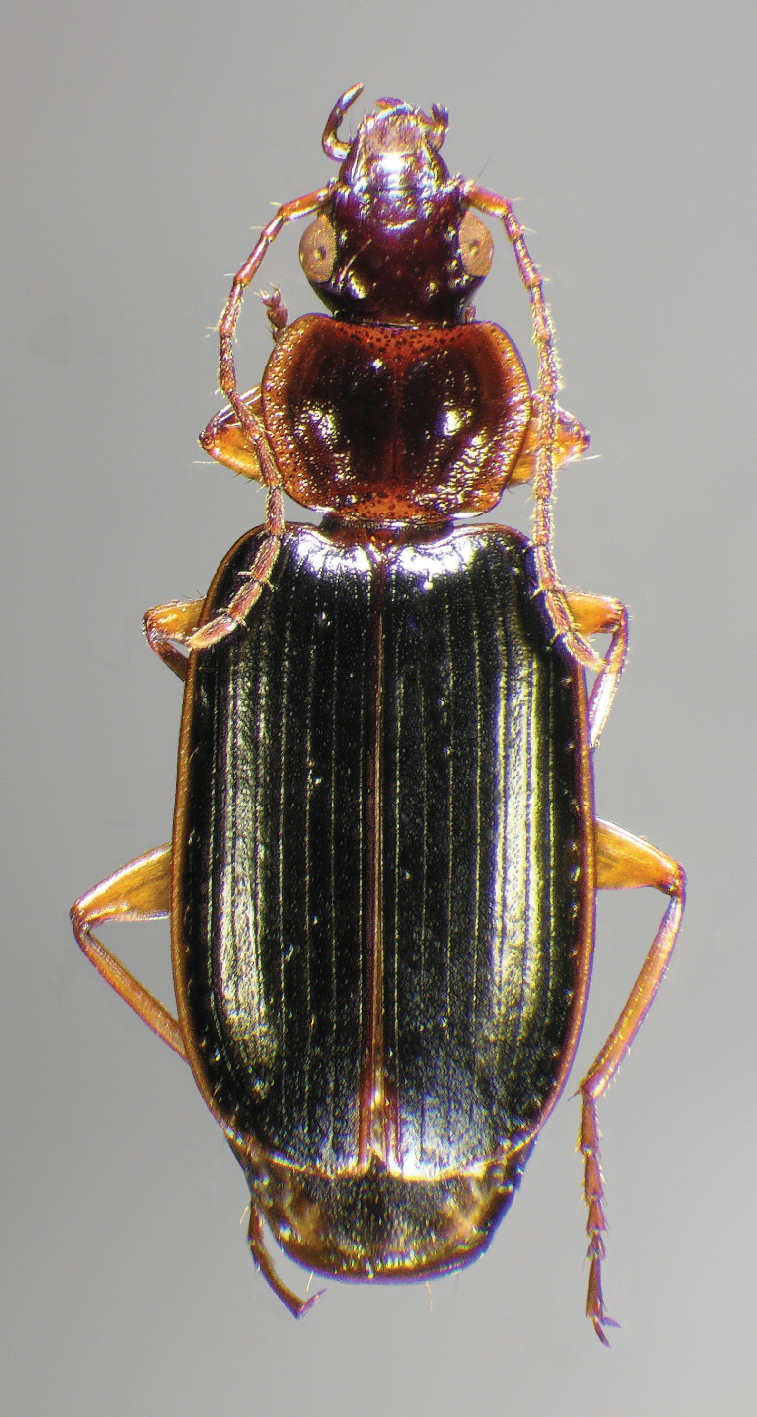
Dorsal habitus and color pattern of *Cymindis platicollis atripennis* (Casey) (OBL 10.33 mm).

**Figure 17. F17:**
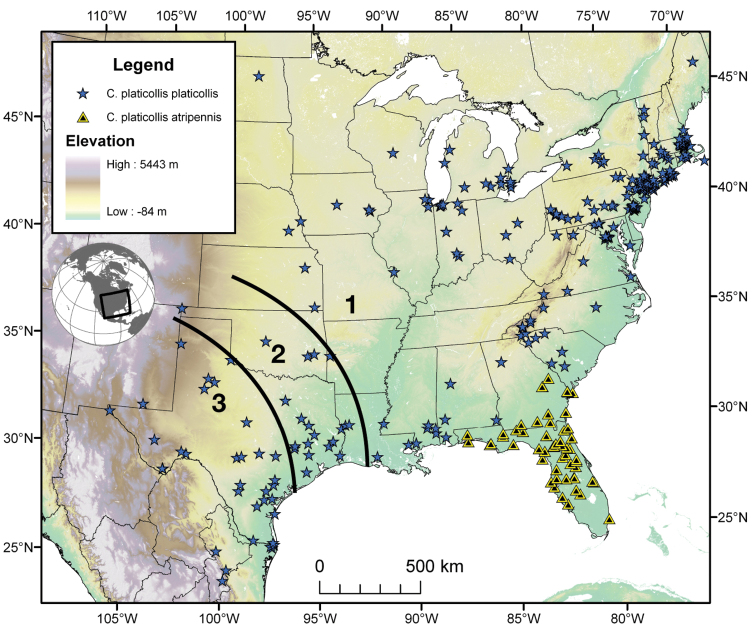
Map of southeastern Canada, U.S.A., and northern Mexico, showing position of localities of the subspecies of *Cymindis platicollis* Say, and the three-stage cline of elytral setation of *Cymindis platicollis platicollis* (Say). See text for explanation.

Figs 16 –17 , 19B , 20A

Pinacodera atripennis Casey 1920: 284. HOLOTYPE male labeled: “Fla; Casey bequest 1925”; “TYPE 47613” [red paper]; “atripennis Csy” [handwritten]. TYPE AREA – Florida, U.S.A. – Kirk 1970: 17. Ciegler 2000: 119. comb. n.
Cymindis atripennis ; Poole and Gentili 1996: 103. [from Ciegler 2000].

#### Diagnosis.

Specimens of this subspecies have a rufous to rufo-brunneous head and pronotum coloration that contrasts strikingly with the darker, brunneo-piceous to rufo-piceous elytral coloration (Fig. 16).

#### Description.

With character states of subgenus *Pinacodera* and species *Cymindis platicollis* restricted as follows: OBL 8.17 – 10.83 mm. Length (n= 10 males, 10 females): head 0.74 – 1.02, pronotum 1.52 – 2.08, elytra 4.92 – 6.42, metepisternum 1.08 – 1.58 mm; width: head 1.64 – 2.04, pronotum 2.08 – 2.72, elytra 3.33 – 4.33, metepisternum 0.58 – 0.84 mm.

*Body proportions*. HW/HL 1.86 – 2.24; PWM/PL 1.30 – 1.40; EL/EW 1.40- 1.52; ML/MW1.71 – 2.41.

*Color*. Dorsum of head and pronotum rufous to rufo-brunneous; antennae rufo-brunneous to brunneous; palpi rufo-brunneous to brunneous; elytra brunneo-piceous to rufo-piceous with pale, somewhat translucent margins, elytral epipleura testaceous to rufo-testaceous; thoracic sclerites and abdominal sterna testaceous to rufo-piceous.

*Microsculpture*. Microlines not visible on dorsum of head capsule and pronotum at 50× magnification. Elytra with mesh pattern isodiametric, microlines clearly defined throughout.

*Macrosculpture and pilosity*. Head capsule, dorsal surface with shallow, randomly scattered setigerous punctures on dorsal surface from constriction of neck extended anteriorly toward clypeus. Elytra with striae moderately impressed and punctulate throughout length; intervals 2, 4, 6 and 8 typically with two to three rows of scattered punctures; all other intervals with one row of punctures.

*Metepisternum*. Distinctly longer than wide.

*Hind wings*. Macropterous.

*Male genitalia*. Phallus (Fig. 20A) length 1.70 – 2.00 mm.

#### Collection notes and habitat.

The known elevational range of *Cymindis platicollis atripennis* extends from sea level to 90 m. Specimens were collected on forest floor, under and on bark of trees and associated mosses in forests of cypress, juniper, magnolia, several oak species and both slash and loblolly pine. This subspecies has been collected from sand dunes in close proximity to water as well as from squirrel and caracara nests in trees. Methods of collecting include: dusk-dawn suction traps, medfly traps, u.v. and light traps, FIT’s, sugaring baits painted on tree trunks, beating vegetation, hand collecting, Steiner traps, pitfall traps, and sticky traps.

#### Geographical distribution.

The range of this subspecies extends from southern Florida north to mid-Georgia and west to southern Mississippi (Fig. 17).

#### Morphological affinities.

This subspecies is, by definition, the closest relative of *Cymindis platicollis platicollis*.

#### Chorological affinities.

*Cymindis platicollis atripennis* is sympatric in portions of its range with *Cymindis limbata*, *Cymindis complanata* and *Cymindis rufostigma*. It is allopatric with *Cymindis platicollis platicollis* and all other species in the *limbata* group.

#### Material examined.

I examined 227 specimens; 13 males and 11 females were dissected. For details see University of Alberta Strickland Virtual Entomology Museum Database (University of Alberta 2009).

### Cymindis (Pinacodera) rufostigma

Hunting sp. n.

urn:lsid:zoobank.org:act:5C506914-55C4-449C-A653-96E29FE32237

http://species-id.net/wiki/Cymindis_rufostigma

**Figure 18. F18:**
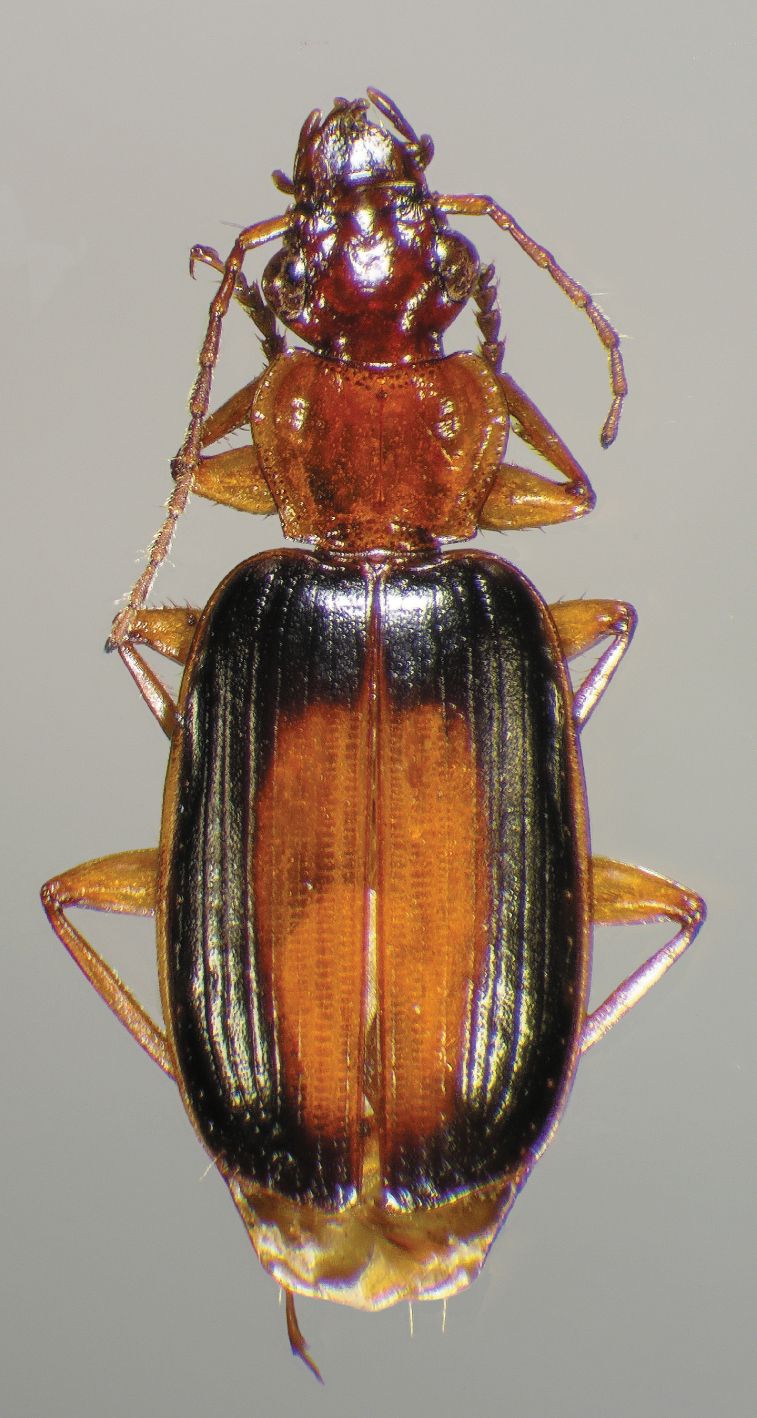
Dorsal habitus and color pattern of *Cymindis rufostigma*, new species (OBL 9.17 mm).

**Figure 19. F19:**
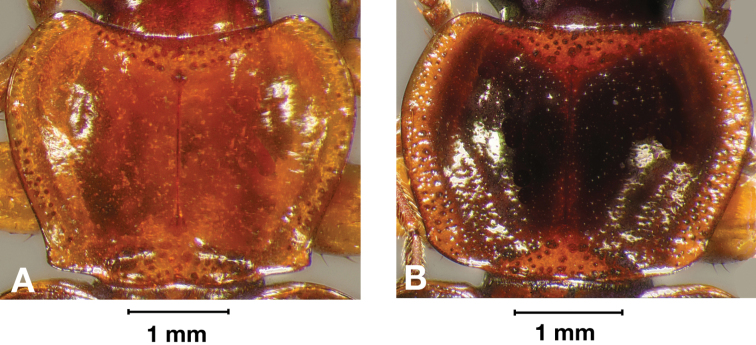
Photographs of pronota of (**A**) *Cymindis rufostigma*, new species, and (**B**) *Cymindis platicollis atripennis* (Casey).

**Figure 20. F20:**
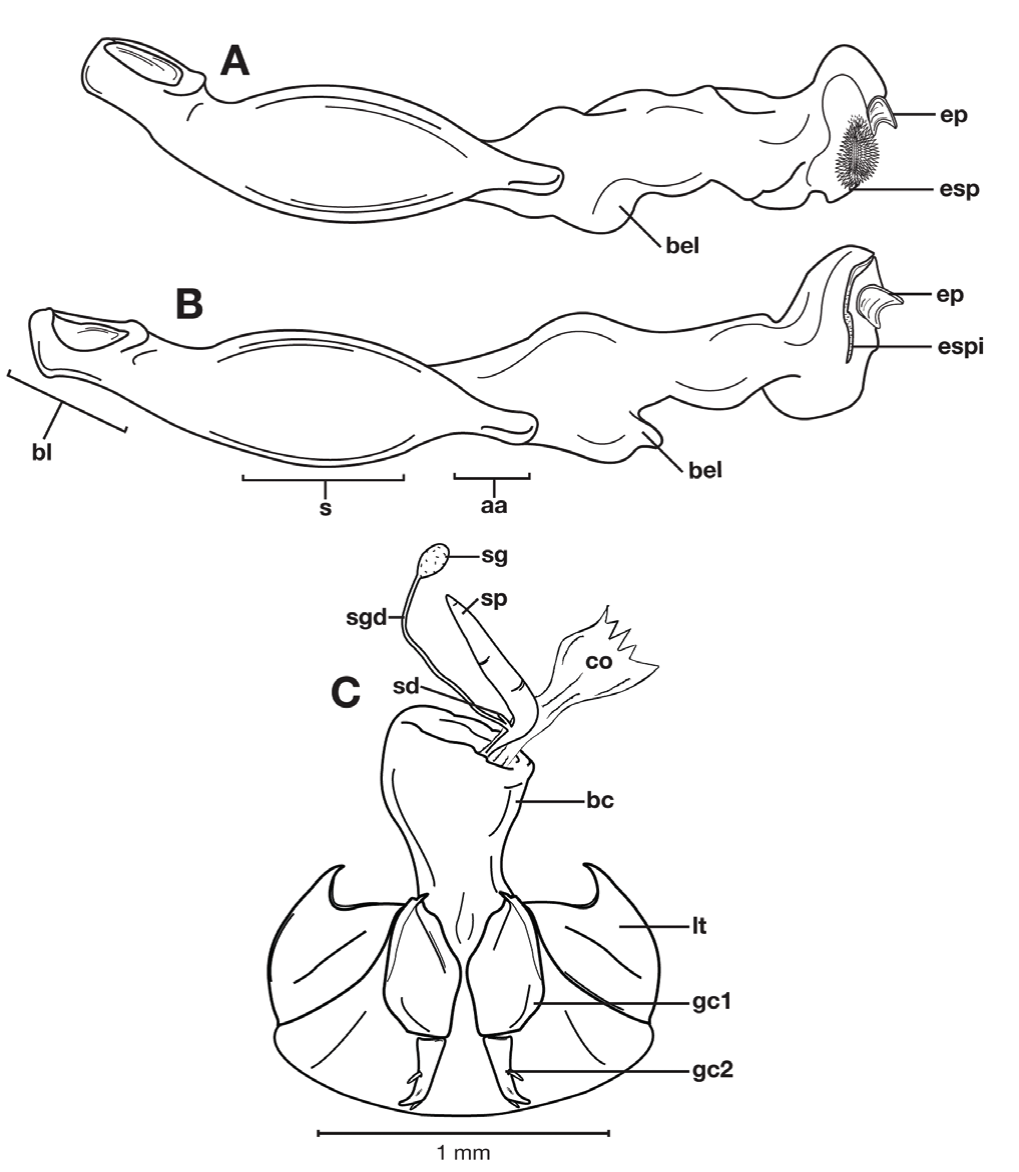
Structural features of male genitalia of (**A**) *Cymindis platicollis atripennis* (Casey), showing appearance of endophallus apex when spine patch is everted, (**B**) *Cymindis rufostigma*, new species, with spine patch not everted, and (**C**) female genitalia of *Cymindis rufostigma*, new species. Legend: **aa**, apical area; **bc**, bursacopulatrix; **bel**, basal endophallic lobe; **bl**, basal lobe; **co**, common oviduct; **ep**, endophallic plate; **esp**, endophallic spine patch; **espi**, endophallic spine patch invagination; **gc1**, gonocoxite 1; **gc2**, gonocoxite 2; **lt**, lateral tergite; **s**, shaft; **sd**, spermathecal diverticulum; **sg**, spermathecal gland; **sgd**, spermathecal gland duct; **sp**, spermatheca.

**Figure 21.**
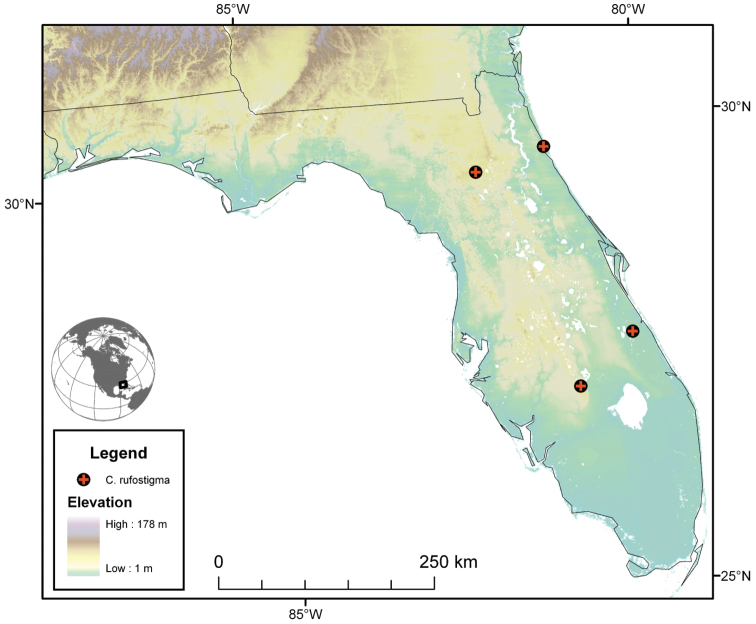
Map of southeastern U.S.A. (primarily Florida), showing position of localities for *Cymindis rufostigma*, new species.

Figs 18 , 19A , 20B-C , 21

#### Type material.

Eleven specimens, labeled as follows. HOLOTYPE male, “FLORIDA: HIGHLANDS CO./ ARCHBOLD BIOL. STA./ 22.IX.1976/ L.L. LAMPERT, JR.”; “U. V. LIGHT” [handwritten]; “UASM#/141621,” [FSCA]. Ten additional PARATYPES, sex and label data as follows. Male, same as holotype except “UASM#/ 202040” [ABSC]. Male, same as holotype except “UASM#/ 141609” [FCSA]. Female, same as holotype except “…19.IV.1976; “UASM#/ 202044 ” [ABSC]. Female, same as holotype except “…17.IX.1977; “UASM#/ 202042” [ABSC]. Female, same as holotype except “…23.IX.1977; “UASM#/ 202041” [ABSC]. Female, same as holotype except “…3.IX.1981/ UVL [handwritten]”; “UASM#/ 202043” [ABSC]. Male, “FLORIDA ALACHUA CO.,/ AUSTIN CARY FOREST/ 28-VI-1976/ G. B. FAIRCHILD”; “INSECT LIGHT TRAP/ #2, CO2-BAITED”; “UASM/ 141619” [FSCA]. Male, “Austin Cary Forest [ handwritten]/ Alachua Co./ Fla. 21.VI.1961 [handwritten]/ ...Hetrick/ ...light trap” [handwritten, partially illegible]”; “UASM#/ 141620” [FSCA]. Female, “FLA. Indian River Co./ SR 512 .5 mi. W. I. 95/ 16-30-III-1977/ Fla. Med. Ent. Lab.”; “sorted from dusk;dawn/ suction trap sample in/ and near bayhead/ M. C. Thomas collection”; “ UASM#/ 141608” [FSCA]. Male, “St. Augustin/ Fla.”; “Liebeck/ Collection”; “UASM#/ 136905” (head missing) [MCZC].

#### Type locality.

Archbold Biological Station, Highlands County, Florida, U.S.A.

#### Specific epithet.

This is a two part feminine Latin noun in apposition, nominative case, based on the adjective *rufus* (red) and the noun *stigma* (mark), referring to the rufo-testaceous central mark on the elytra of adult members of this species.

#### Diagnosis.

This species (Fig. 18) differs from others in the following ways: testaceous head and pronotum, pronotum with posteriolateral margins right- angled (Fig. 19A, *cf.* 19B) and elytra with large oblong rufous central macula. Male genitalia with distinct basal endophallic lobe (**bel**) (Fig. 20B).

#### Description.

With character states of subgenus *Pinacodera* restricted as follows: OBL. 9.1 – 10.3 mm. Length (n= 7 males, 3 females): head 0.88 – 1.00 , pronotum 1.52 – 1.76, elytra 5.25 – 6.00, metepisternum 1.26 – 1.50 mm; width: head 1.72 – 2.00, pronotum 2.10 – 2.44, elytra 3.42 – 3.92, metepisternum 0.54 – 0.84 mm.

*Body proportions*. HW/HL 1.82 – 2.04; PWM/PL 1.29 – 1.45; EL/EW 1.48 – 1.58; ML/MW 1.92 – 2.41.

*Color*. Dorsum of head and pronotum rufous to rufo- testaceous; antennae rufo-testaceous to rufo-brunneous; palpi rufo-testaceous to brunneous; elytra brunneo-piceous to rufo-piceous with large oblong rufo-testaceous central macula and pale, somewhat translucent margins; elytral epipleura testaceous to rufo-testaceous; thoracic sclerites and abdominal sterna testaceous to rufo-piceous.

*Microsculpture*. Microlines not visible on dorsum of head capsule and pronotum at 50× magnification. Elytra with mesh pattern isodiametric, microlines clearly impressed throughout.

*Macrosculpture and pilosity*. Head capsule with shallow, randomly scattered setigerous punctures on dorsal surface from constriction of neck extended anteriorly toward clypeus. Elytra with striae moderately impressed and punctulate throughout length; intervals 2, 4, 6 and 8 typically with two to three rows of scattered punctures; all other intervals with one row of punctures; some specimens with intervals 1, 3 and 5 somewhat raised toward apex.

*Metepisternum*. Distinctly longer than wide.

*Hind wings*. Macropterous.

*Male genitalia*. Phallus (Fig. 20A) length 1.70 – 2.00 mm. Endophallus with basal endophallic lobe (**bel**) distinctly formed.

#### Habitat and seasonal occurrence.

The known elevational range of *Cymindis rufostigma* extends from sea level to 65 m. Some specimens were collected in stands of slash pine. Methods of collecting include: dusk-dawn suction traps, u.v. light traps, CO2-baited insect light traps.

#### Geographical distribution.

The range of this species extends from southern Florida north to northern Florida (Fig. 21).

#### Chorological affinities.

*Cymindis rufostigma* is sympatric in portions of its range with *Cymindis platicollis atripennis* and *Cymindis limbata*. It is allopatric with *Cymindis platicollis platicollis* and all other species in the *limbata* group.

#### Material examined.

The type material. See above for details.

### Cymindis (Pinacodera) punctigera complex

#### Remarks.

The *punctigera* complex includes only a single species; see *Cymindis punctigera* LeConte below for details.

### Cymindis punctigera

LeConte

http://species-id.net/wiki/Cymindis_punctigera

**Figure 22.**
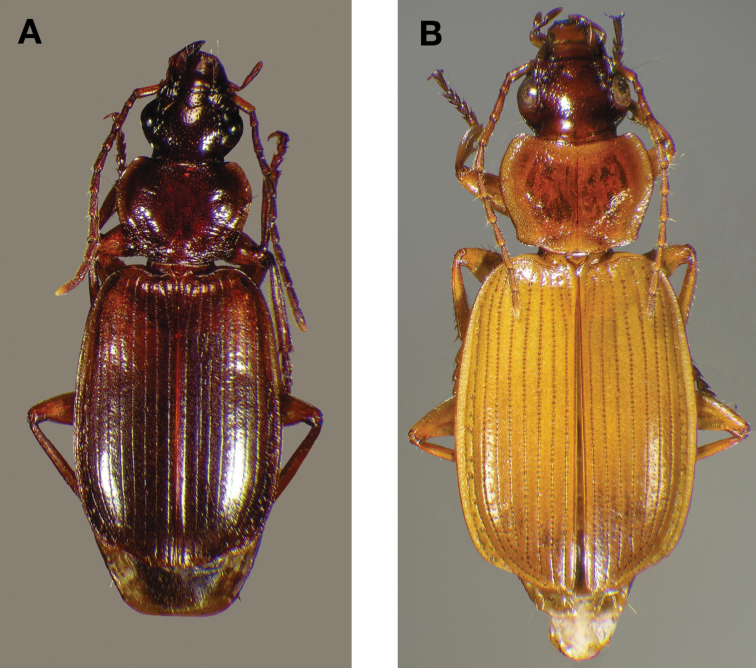
Dorsal habitus and color pattern of *Cymindis punctigera punctigera* LeConte: **A**, showing typical dorsal coloration (OBL 10.17 mm); **B**, showing coloration of some specimens from the Big Bend area of southwestern Texas, U.S.A. (OBL 11.83 mm).

Figs 22 [Fig F23] [Fig F24] [Fig F25] [Fig F26] [Fig F27] [Fig F28] –29

#### Remarks.

This is a polytypic species that includes two subspecies (*Cymindis punctigera punctigera* and *Cymindis punctigera sulcipennis*). The basis for including these forms in a single species is as follows: first, similarity in body proportions; second, males exhibit similarity in details of the apical area of the phallus (Fig. 27B-C) and everted endophallus form (Fig. 27A); and third, allopatric but proximate geographical distribution.

#### Diagnosis.

Adults of *Cymindis punctigera* are distinguishable from those of other species of the *limbata* species group through the unique combination of: pronotum with scattered and distinct punctures (Fig. 24), one row of punctures in each each elytral interval, and dorsal color that ranges from testaceous to dark brown, some specimens approaching piceous, but if so, the elytral margins are translucently bordered; in males the apical area of the phallus is dimpled on both the dorsal and ventral surfaces, longitudinal striations are visible from mid-way of apical area and extended past constriction (Fig. 27B-C), and the endophallus is distinctly bent to the right and dorsad when everted and viewed from the dorsal aspect (Fig. 27A).

**Description.** OBL 7.75-11.33 mm.

**Figure 23. F23:**
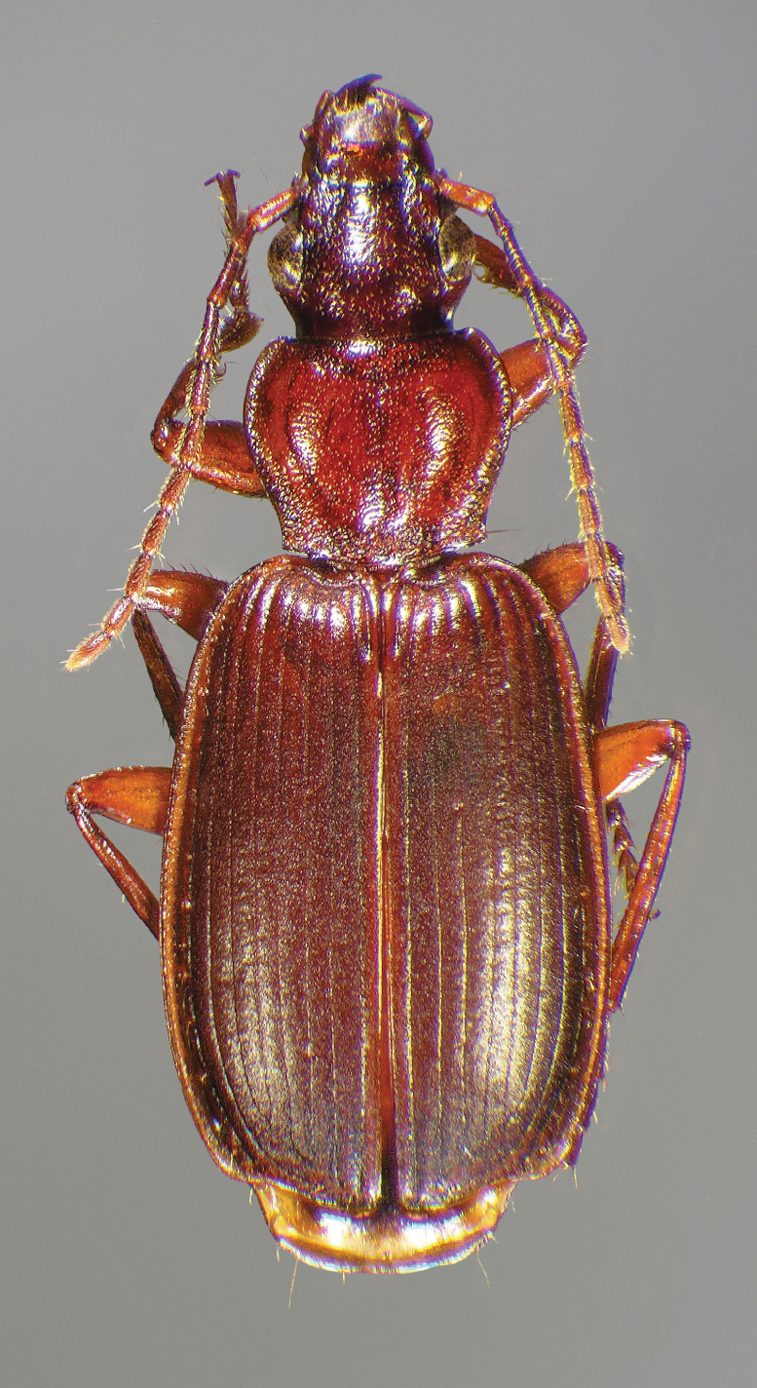
Dorsal habitus and color pattern of *Cymindis punctigera sulcipennis* (Horn) (OBL 10.83 mm).

*Color*. (Figs 22A-B, 23); Dorsum of head testaceous (Fig. 22B) to rufo-piceous (Fig. 22A), dorsum of pronotum and elytra testaceous (Fig. 22B) to rufo-piceous (Fig. 22A), rarely with lighter colored elytral margins; antennae testaceous to rufo-piceous, palpi testaceous to rufo-piceous; epipleura testaceous to rufo-piceous; Ventral thoracic sclerites and abdominal sterna testaceous to rufo-piceous.

*Microsculpture*. Most individuals with microlines not visible on dorsum of head capsule and pronotum at 50× magnification; few specimens with mesh pattern isodiametric to transverse between eyes and on disc of pronotum. Elytra with mesh pattern isodiametric, microlines clearly defined throughout dorsal surface.

*Macrosculpture and pilosity*. Head capsule with randomly scattered setigerous punctures on dorsal surface from constriction of neck extended anteriorly toward clypeus. Pronotum dorsally with shallow and randomly spaced setigerous punctures with setae length ranging from very short to moderate; ventrally with setigerous punctures extended from margin of proepipleuron to apex of prosternal intercoxal process. Elytra with striae moderately impressed and punctulate throughout length; intervals slightly convex to markedly raised towards center of interval; single regular to moderately irregular row of ~50–65 setigerous punctures within each interval, setae length ranging from very short to moderate at 50×. Abdominal sterna with fine pilose punctures throughout.

*Fixed setae*. Pronotum with two setae along each margin. Elytra with two setae in stria 3 and one posteriad of stria 3; one seta at apex of interval 2; 15–17 lateral (umbilical) setae**;** two setae on each of abdominal sterna III to VI; four setae along apical margin of sternum VII (Fig. 3).

*Luster*. Head capsule and pronotum distinctly to slightly glossy; elytra glossy to rather dull; ventral thoracic sclerites and abdominal sterna glossy.

*Pronotum*. Anterior and posterior transverse impression shallow; median longitudinal impression moderately shallow; posteriolateral angles from almost right angled to slightly obtuse; posterior margin slightly lobate.

*Head* (Figs 22A-B, 23). Eyes, labrum, labium and palpi typical for Cymindidina.

*Elytra* (Figs 22A-B, 23). Humeri broadly or narrowly rounded, striae moderately impressed; lateral margin smooth, rounded and widened preapically; elytral apices truncate.

*Hind wings* (Figs 28A-D). Macropterous (Figs 28A, C) or brachypterous (Figs 28B, D).

*Legs*. Males with adhesive vestiture ventrally, two rows of squamo- setae on tarsomeres 1–4 of foreleg and 1–3 of middle leg.

*Male genitalia* (Figs 26A, 27A-C). Phallus anopic, cylindrical, ventral surface slightly curved. Ventral and dorsal surface of apical area (**aa**) somewhat to markedly dimpled in appearance, few to several vertical striations extended from mid length of apical area to apex of phallic shaft (**s**) (Fig. 27B-C). Endophallus with a slightly curved endophallic plate (**ep**) (Lindroth 1969: 1080–1081) apically (Fig. 27A), when viewed ventrally in everted condition

*Female genitalia*. Gonocoxite 2 (**gc2**) (Fig. 26B) moderately long and narrow. Internal genitalia with long cylindrical spermatheca (**sp**), moderately long associated spermathecal gland (**sg**), and moderately long spermathecal diverticulum (**sd**) located at base of spermathecal gland duct (**sgd**).

#### Geographical distribution.

The range of this species extends (Fig. 29) in the southwestern United States from Lake Tahoe, California south through southern Utah and Nevada to western Texas; south in eastern Mexico to Nuevo Leon in the northern portion of the Sierra Madre Oriental; in western Mexico, on the slopes of the Sierra Madre Occidental to Jalisco, and in the Sierra Transvolcanica, to Michoacan. Further west this species ranges through most of the Baja California Peninsula.

#### Chorological affinities.

*Cymindis punctigera* is sympatric in the southern portion of its range with *Cymindis chevrolati* and *Cymindis platicollis platicollis*.

### Cymindis punctigera punctigera

LeConte subsp. n.

http://species-id.net/wiki/Cymindis_punctigera_punctigera

Figs 22 , 25A , 26 [Fig F27] [Fig F28] –29

Cymindis punctigera LeConte 1851: 178, Type material: two males, one female, LeConte Collection [MCZC]. LECTOTYPE (here selected), first male, labeled: disc [green-gold]; Type [white paper] “66”[red paper]; “P. punctigera LeC Col” [handwritten]. PARALECTOTYPE; second specimen female, labeled same as lectotype, and “punctigera 2”; third specimen male, labeled same as lectotype, and “punctigera 3”. (Two more specimens, each labeled “Ariz” and “punctigera 4” and “punctigera 5”, respectively, were probably assigned to this species at a later date and are not recognized as types).
Pinacodera punctigera (LeConte 1851); Chaudoir 1875: 4. – Horn 1882: 148. – Bates 1884: 296. – Fall and Cockerell 1907: 160. – Casey 1920: 284.
Apenes punctigera Blackwelder 1944: 62.
Cymindis blanda Casey 1913: 184. Type material three females, Casey collection [USNM]. LECTOTYPE (here selected), labeled: “Douglas Ariz. Aug. F. H. Snow; San Bernardino Ranch 3750 ft”; “TYPE USNM 47608” [red paper]; “blanda Csy” [handwritten]. PARALECTOTYPES each labeled similarly to the lectotype, and labeled “paralectotype 2” and “paralectotype 3” respectively. TYPE LOCALITY. – San Bernardino Ranch, 24 kilometers east of Douglas**,** Cochise County, Arizona, U.S.A. sp. n.
Pinacodera blanda (Casey); 1920: 281.
Pinacodera subcarinata Casey 1920: 281. HOLOTYPE female labeled: “Ariz; Casey 1925”; “TYPE USNM 47609”; “subcarinata Csy” [handwritten] [USNM]. syn. n.

#### Type area.

**“**Near the junction of the Colorado and Gila Rivers,” Yuma County, Arizona, U.S.A.

#### Notes about types, homonymy, and synonymy.

The reason for assigning *Cymindis punctigera* to *Apenes* (Blackwelder 1944: 62) is not apparent. The type specimens of the named forms, taken in isolation, are distinctive and differ from one another as previous authors indicate. Examination of material geographically adjacent to these forms reveals that this species is quite variable throughout its range, and characters from Casey’s descriptions are not diagnostic.

#### Diagnosis.

Specimens of this subspecies (Fig. 22A-B) exhibit little to no rugosity on dorsal surface of head from between eyes toward clypeus.

#### Description.

With character states of subgenus *Pinacodera* and species *Cymindis punctigera* restricted as follows: OBL 7.75 - 11.33 mm. Length (n= 10 males, 10 females): head 0.72 – 1.04, pronotum 1.40 – 2.34, elytra 4.41 – 6.91, metepisternum 0.94 – 1.54 mm; width: head 1.60 – 2.28 , pronotum 1.80 – 2.92, elytra 3.16 – 4.58, metepisternum 0.52 – 0.88 mm.

*Body proportions*. HW/HL 2.0 – 2.32; PWM/PL 1.18 – 1.31; EL/EW 1.28- 1.51; ML/MW 1.44 – 1.96.

*Color* (Fig. 22A-B). Dorsum of head testaceous to rufo-piceous, dorsum of pronotum and elytra testaceous to rufo-piceous, rarely with lighter colored elytral margins; antennae testaceous to rufo-piceous; palpi testaceous to rufo-piceous; epipleura testaceous to rufo-piceous. Ventral thoracic sclerites and abdominal sterna testaceous to rufo-piceous.

*Metepisternum*. Individuals are at least 1.44× as long as wide.

*Hind wings*. Macropterous or brachypterous.

*Male Genitalia*. Phallus (Fig. 26A) length 1.70 – 2.04 mm.

#### Variation.

Within this subspecies, dorsal pilosity, body coloration and wing state are, to some extent, variable geographically. The least understood is seta length on the dorsal surface of individuals. In some areas (mainly montane) populations of *Cymindis punctigera punctigera* have uniformly long setae covering their dorsal surface while others from surrounding localities have setae so short that they are hardly visible at 50× magnification.

Color of the dorsal surface (Fig. 22A-B) varies from dark rufous over most of the species range (Fig. 22A) to predominantly testaceous to rufo- testaceous (Fig. 22B) in the Big Bend area of southwestern Texas.

Wing state and wing length vary in this subspecies. Examination of a few hundred individuals, from all known localities showed that *Cymindis punctigera punctigera* exhibits a general trend toward brachyptery in the periphery of its range and macroptery in the central portion (Fig. 29). Population samples from two localities (Washington Co., Utah and Riverside Co., California) included both macropterous and brachypterous individuals. As well, average length of wing rudiments of brachypterous specimens differed between localities with the shortest being from Nuevo Leon, the same place where members of *Cymindis chevrolati* exhibit the shortest average wing rudiments.

#### Collection notes and habitat.

The known elevational ranges of *Cymindis punctigera punctigera* extends from 670 to 2500 m. Most specimens were found at elevations higher than 1000 m. Specimens were collected under stones and bark of trees in forests of oak, pine, mesquite (*Prosopis glandulosa* Torrey), in stands of acacia and desert willow (*Chilopsis linearis* Cav.). As well they occupy riparian temperate forests, meadow, desert and pond margin habitats. This subspecies has been collected from nests of packrats of the genus *Neotoma*.

#### Geographical distribution.

The range of this subspecies (Fig. 29) extends in the southwestern United States from Lake Tahoe, California south through southern Utah and Nevada to western Texas south to the interior of Mexico, both sides of the Sierra Madre Occidental, east as far east as Nuevo Leon, south to Michoacan and as far west as Nayarit.

#### Morphological affinities.

This subspecies is by definition the closest relative of *Cymindis punctigera sulcipennis*.

#### Chorological affinities.

*Cymindis punctigera punctigera* is allopatric with *Cymindis punctigera sulcipennis* and all other species of the *limbata* group except *Cymindis chevrolati* and *Cymindis platicollis platicollis*, which are sympatric with it toward the eastern limits of its range.

#### Material examined.

I examined 385 specimens: 72 males and 98 females were dissected. For details see University of Alberta Strickland Virtual Entomology Museum Database (University of Alberta 2009).

### Cymindis punctigera sulcipennis

(Horn) stat. n.

http://species-id.net/wiki/Cymindis_punctigera_sulcipennis

**Figure 24. F24:**
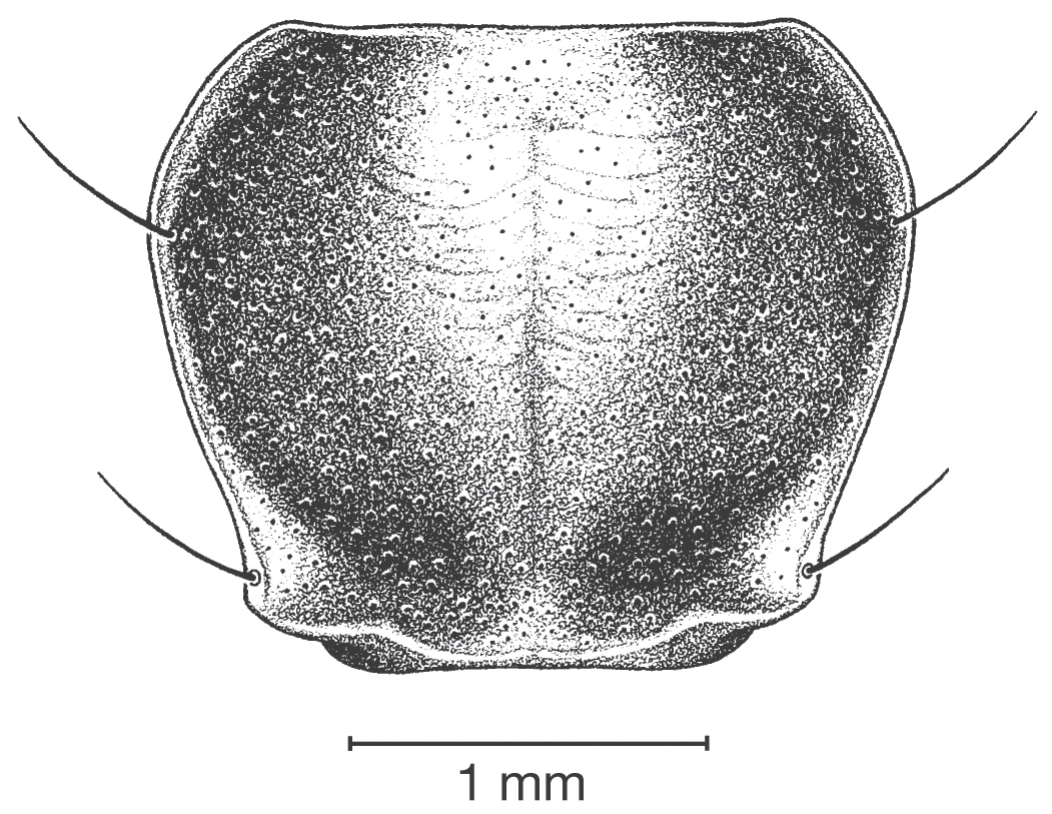
Pronotum, dorsal aspect, of *Cymindis punctigera sulcipennis* (Horn).

**Figure 25. F25:**
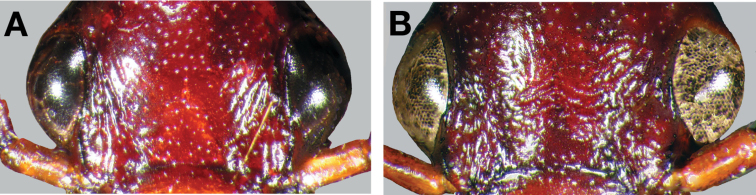
Head capsule, dorsal aspect, showing frontal macrosculpture: **A**, *Cymindis punctigera punctigera* LeConte; **B**, *Cymindis punctigera sulcipennis* (Horn), note transverse lines between eyes.

Figs 23 –24 , 25B , 29

Pinacodera sulcipennis Horn 1881: 40. HOLOTYPE male, labeled: “Cal”; [small yellow paper rectangle]; “HOLOTYPE 2932” [red paper]; “*Pinacodera sulcipennis*” Horn [handwritten]. [MCZC]. – Horn 1882: 147–148. – 1894: 310. TYPE AREA – Baja California, Mexico syn. n.
Pinacodera semisulcata Horn 1881: 40. HOLOTYPE, male labeled: “Cal”; [small yellow paper rectangle]; “*Pinacodera semisulcata*” Horn [handwritten]. [MCZC]. TYPE AREA - Baja California Sur – Horn 1882: 147–149. – 1894: 310. syn. n.

#### Type locality.

La Paz, Baja California Sur, Mexico, here designated.

#### Notes about synonymy.

Horn (1881:40) distinguished *Pinacodera sulcipennis* from *Pinacodera semisulcata* through the former having raised elytral intervals. Careful examination of more than 80 specimens revealed that some members of this subspecies (32%) have raised elytral intervals, especially in the basal third. This is the rationale for combining *Pinacodera semisulcata* with *Pinacodera sulcipennis*.

#### Diagnosis.

Most specimens of this subspecies (94.2%) have two to several rugulose transverse lines on dorsal surface of head between eyes with rugosity in some extended to clypeus (Fig. 25B).

#### Description.

With character states of subgenus *Pinacodera* and species *Cymindis punctigera* restricted as follows: OBL 8.26 – 10.67 mm. Length (n= 20 males, 20 females): head 0.80 – 1.02, pronotum 1.72 – 2.20 , elytra 4.50 – 6.12, metepisternum 0.94 – 1.48 mm; width: head 1.72 – 2.20 , pronotum 2.00 – 2.64, elytra 3.12– 4.33, metepisternum 0.45 – 0.86 mm.

*Body proportions*. HW/HL 1.91 – 2.30; PWM/PL 1.08 – 1.25; EL/EW 1.26 – 1.49; ML/MW 1.40 – 1.88.

*Color* (Fig. 23). Dorsum of head rufous to rufo-piceous; dorsum of pronotum and elytra rufous to rufo-piceous; antennae rufo- testaceous to rufo- piceous; palpi rufo-testaceous to rufo-piceous; elytral epipleura rufo-testaceous to rufo-piceous; abdominal sterna and other thoracic sclerites rufo-testaceous to rufo-piceous.

*Elytra* (Fig. 23). Humeri narrowed.

*Hind wings*. Brachypterous.

*Male Genitalia* Phallus (*cf*. Fig. 24A-C) length 1.82–2.00 mm.

**Figure 26. F26:**
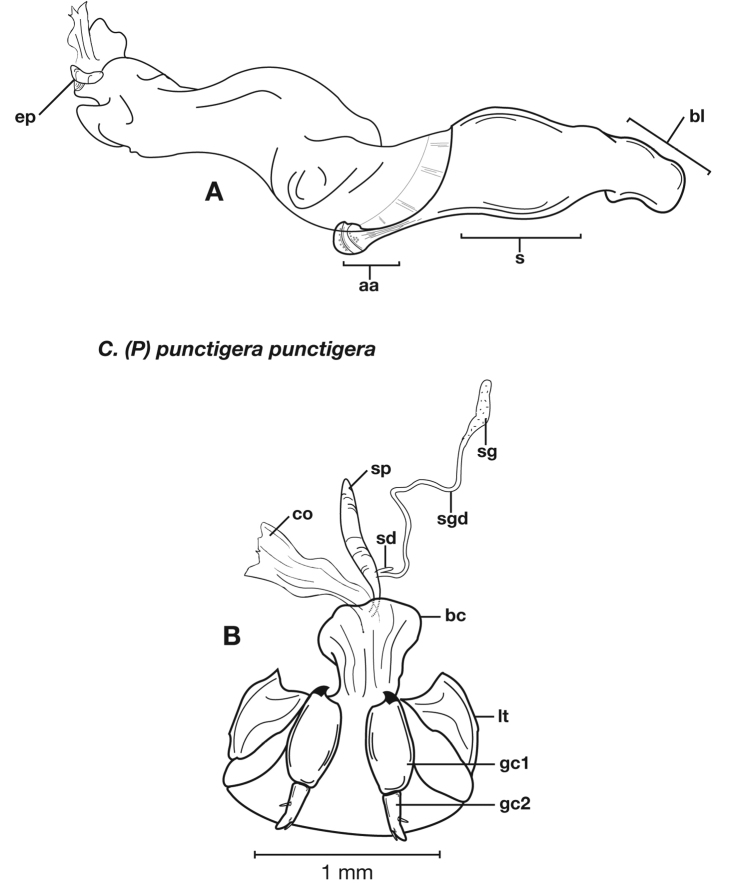
Structural features of *Cymindis punctigera punctigera* LeConte: **A**, phallus and everted endophallus, right lateral aspect; **B**, female reproductive tract and ovipositor, ventral aspect. Legend: **aa**, apical area; **bc**, bursa copulatrix; **bl**, basal lobe; **co**, common oviduct; **ep**, endophallic plate; **gc1**, gonocoxite 1; **gc2**, gonocoxite 2; **lt**, lateral tergite; **s**, shaft; **sd**, spermathecal diverticulum; **sg**, spermathecal gland; **sgd**, spermathecal gland duct; **sp**, spermatheca.

**Figure 27. F27:**
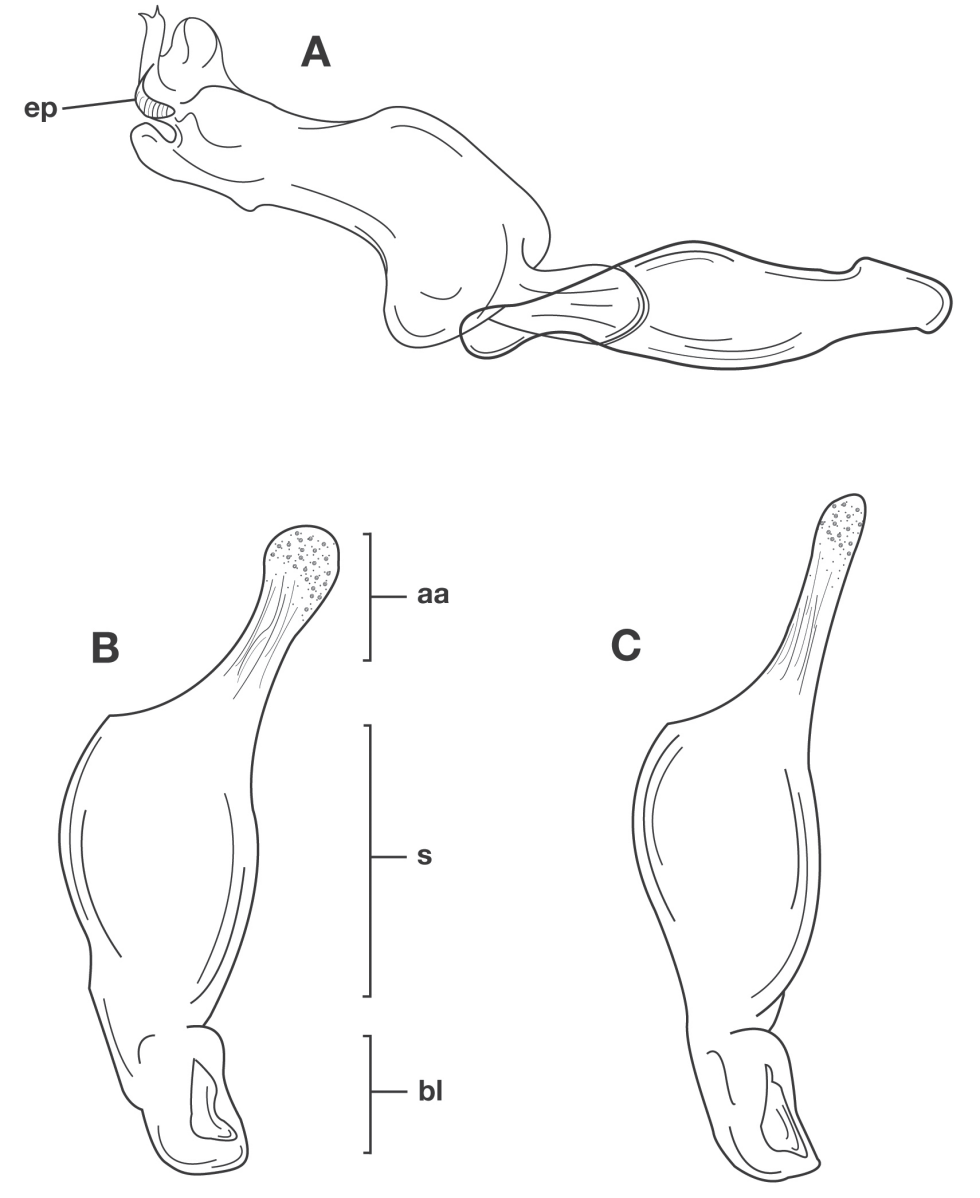
Male genitalia of *Cymindis punctigera punctigera* LeConte: **A**, phallus and everted endophallus, dorsal aspect, showing right-hand placement of endophallus when everted; **B**-**C**, left lateral aspect of phallus, endophallus inverted, showing intrapopulation variation (Jeff Davis Co., Texas, U.S.A.) in form of phallic apex; texture and distinctive striations extended from mid-apical area toward shaft. Legend: **aa**, apical area; **bl**, basal lobe; **ep**, endophallic plate; **s**, shaft.

**Figure 28. F28:**
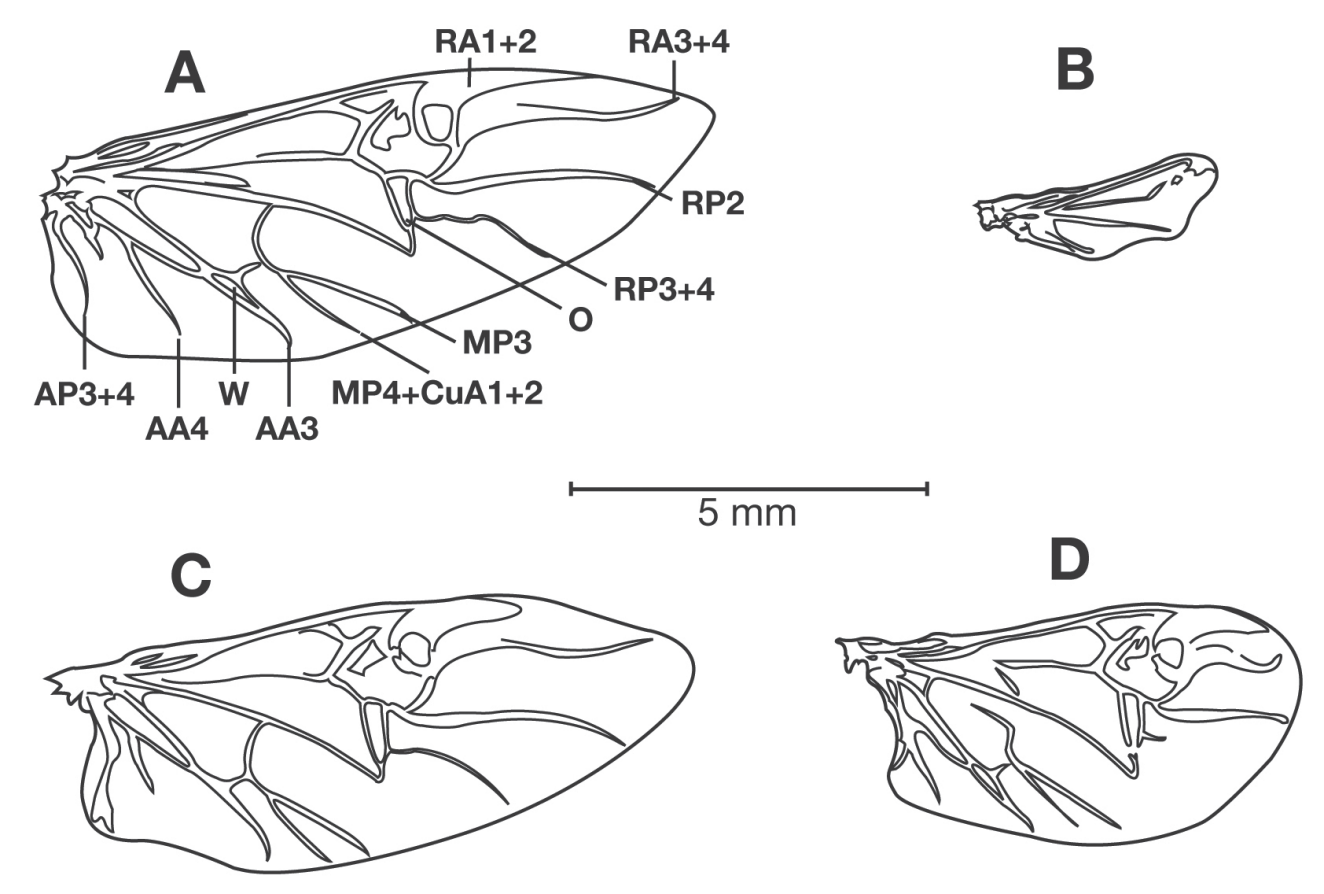
Right hindwings of *Cymindis punctigera punctigera* LeConte, dorsal aspect: A-B, extremes of intraspecific variation from functional (**A**) to markedly atrophied (**B**); **C**-**D**, showing intrapopulation variation (Cloudcroft, New Mexico, U.S.A.) from functional (**C**) to somewhat atrophied (**D**). Legend: (from Kukalova-Peck and Lawrence 2004) **AA3**, anterior anal 3; **AA4**, anterior anal 4; **AP3+4**, posterior anal 3+4; **MP3**, posterior median 3; **MP4+CuA1+2**, posterior median 4 + anterior cubitus 1+2; **O**, Oblongum cell; **RA1+2**, anterior radius 1+2; **RA3+4**, anterior radius 3+4; **RP2**, posterior radius 2; **RP3+4**, posterior radius 3+4; **W**, wedge cell.

#### Collection notes and habitat.

The known elevational range of *Cymindis punctigera sulcipennis* extends from 140 to 1850 m. Specimens have been collected from stands of yucca and on shrubs of the species *Euphorbia misera* Benth.

#### Geographical distribution.

**Figure 29.**
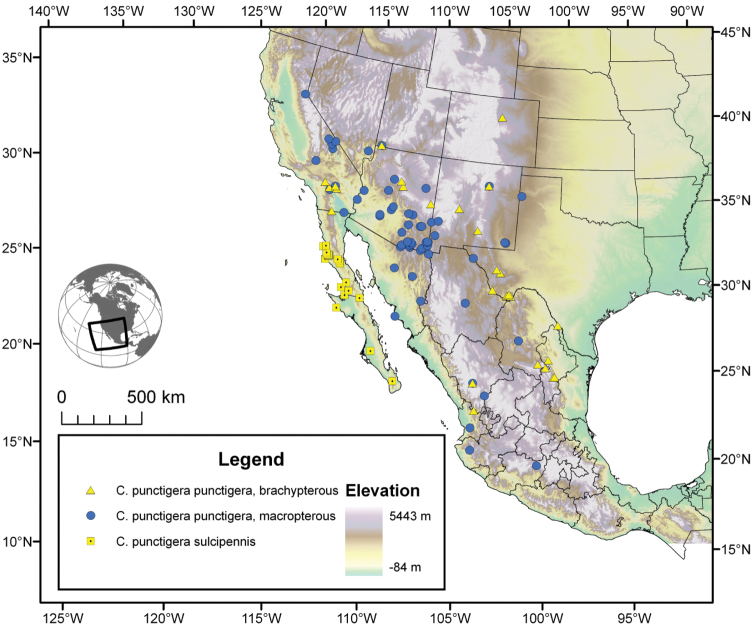
Map of southwestern U.S.A. and northern Mexico, showing position of localities of the subspecies of *Cymindis punctigera* LeConte, and distribution of macropterous and brachypterous individuals of *Cymindis punctigera punctigera*.

This subspecies is restricted to Baja California in Mexico (Fig. 29), ranging from the southern tip of the peninsula, to as far north as San Quentin, Baja California Norte.

#### Morphological affinities.

This subspecies is by definition the closest relative of *Cymindis punctigera punctigera*.

#### Chorological affinities.

*Cymindis punctigera sulcipennis* is allopatric with *Cymindis punctigera punctigera* and with all other taxa of the limbata group.

#### Material examined.

I have examined 84 specimens; 10 males and 10 females were dissected. For details see University of Alberta Strickland Virtual Entomology Museum Database (University of Alberta 2009).

### Cymindis (Pinacodera) chevrolati complex

Figs 30 [Fig F31] [Fig F32] [Fig F33] [Fig F34] [Fig F35] [Fig F36] [Fig F37] [Fig F38] [Fig F39] [Fig F40] –41

#### Diagnosis.

**Diagnosis.** Dorsal surface of the pronotum and elytra entirely black in combination with the notably deeper, more irregular single row of punctures in each elytral interval, distinguish members of the *chevrolati* complex from all other species in the *limbata* species group.

#### Description.

**Figure 30.**
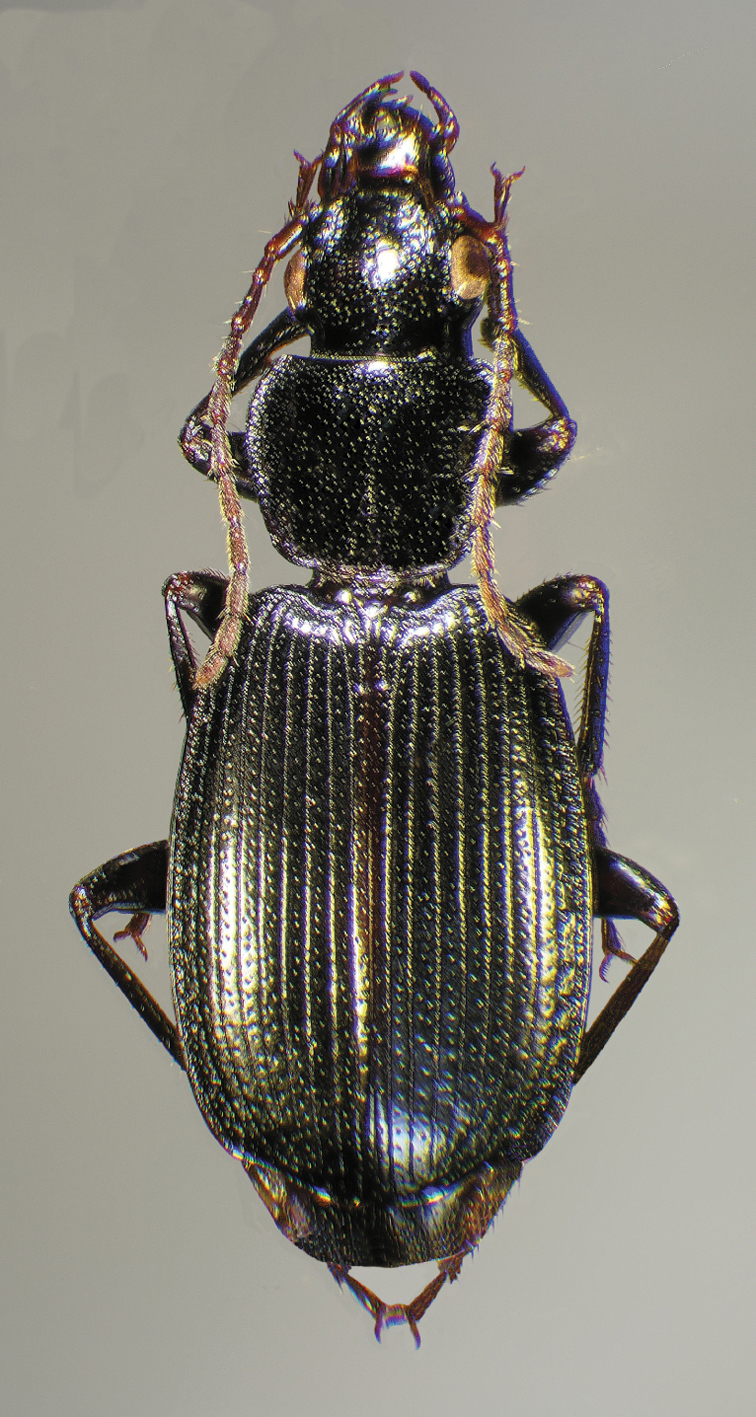
Dorsal habitus and color pattern of *Cymindis chevrolati* Dejean (OBL 12.00 mm).

*Color* (Fig. 30, 32–33). Dorsum of head black to rufo-piceous; dorsum of pronotum and elytra black; antennae rufo-piceous to rufo-testaceous; palpi rufo-testaceous; elytral epipleura, ventral thoracic sclerites, and abdominal sterna rufo-piceous to piceous; legs piceous.

*Microsculpture*. Head capsule and pronotum smooth, microlines not evident at 50×. Elytra with mesh pattern isodiametric, microlines shallow to not apparent at 50×.

*Macrosculpture and pilosity*. Head capsule with evenly scattered setigerous punctures on dorsal surface from constriction of neck extended anteriorly toward clypeus. Prothorax ventrally with fine setigerous punctures extended from lateral margin of coxal cavity to apex of intercoxal process. Elytra with striae moderately impressed and punctulate throughout length; intervals slightly convex, single irregular row of ~30–45 punctures within each interval. Abdominal sterna with pilose punctures throughout, setae increased slightly in length toward baso-lateral margins.

*Fixed setae*. Two pairs of supraorbital setae; clypeus with two lateral setae. Labrum with six setae along apical margin. Pronotum with two to five setae along each margin. Elytra with two seta in stria 3 and one beyond apex of stria 3; one setae at apex of interval two; 15–17 umbilical setae;two setae on each of abdominal sterna III to VI; 4–8 setae along apical margin of sternum VII (Fig. 3).

*Luster*. Head capsule and pronotum glossy; elytra glossy to slightly glossy, ventral thoracic sterna and abdominal sterna glossy.

**Figure 31. F31:**
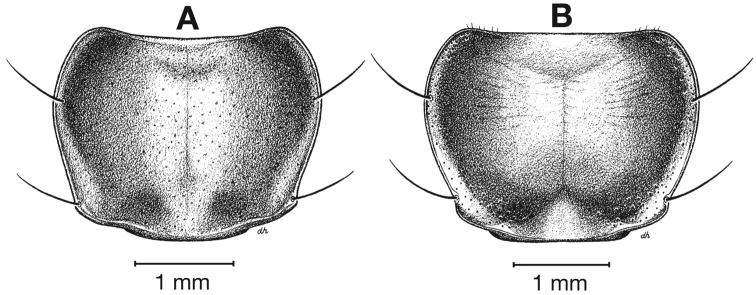
Pronota, dorsal aspect, of *Cymindis chevrolati* Dejean, showing intrapopulation variation in pronotal punctulation: **A**, typical, scattered and irregular punctation; **B**, less common, pronotal disc almost smooth with punctation more visible toward margins.

**Figure 32. F32:**
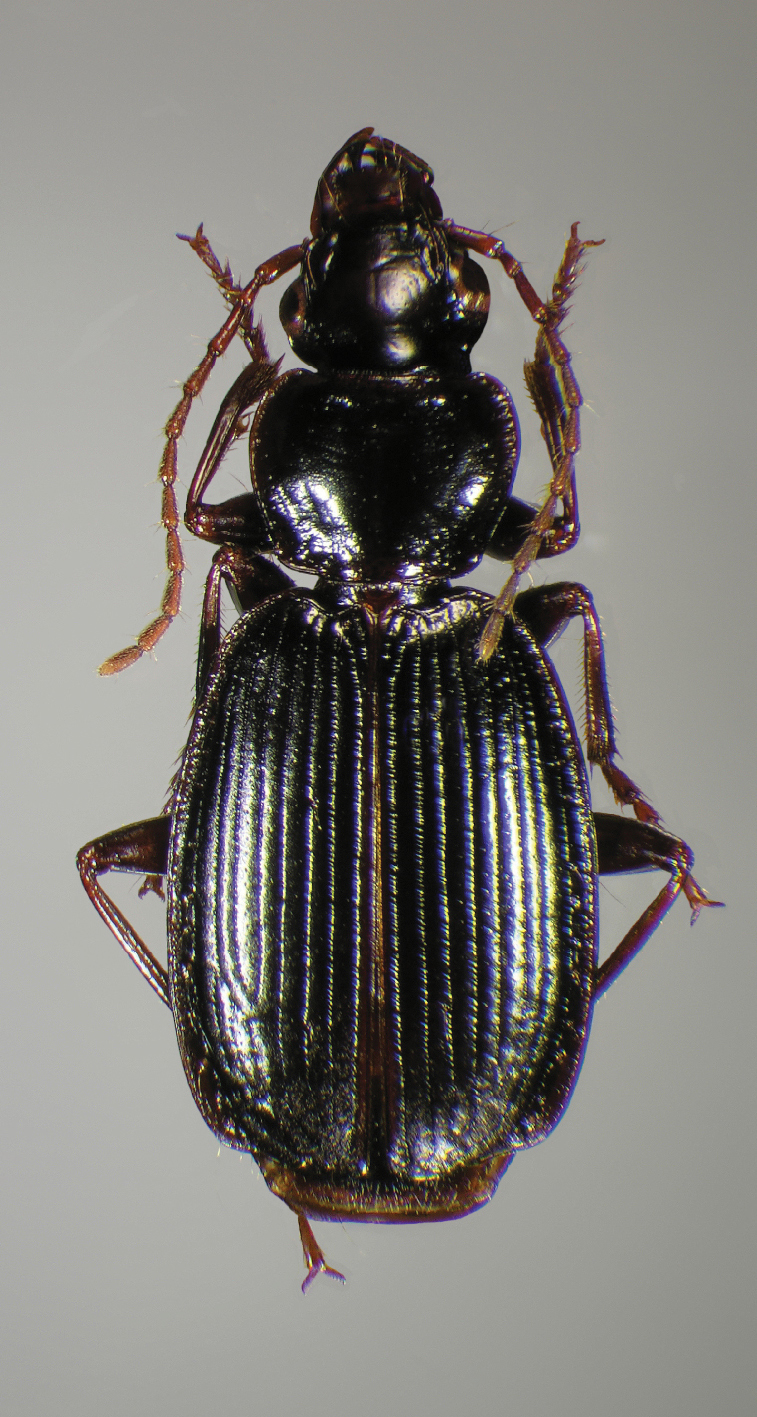
Dorsal habitus and color pattern of *Cymindis laevior* (Bates) (OBL 11.67 mm).

**Figure 33. F33:**
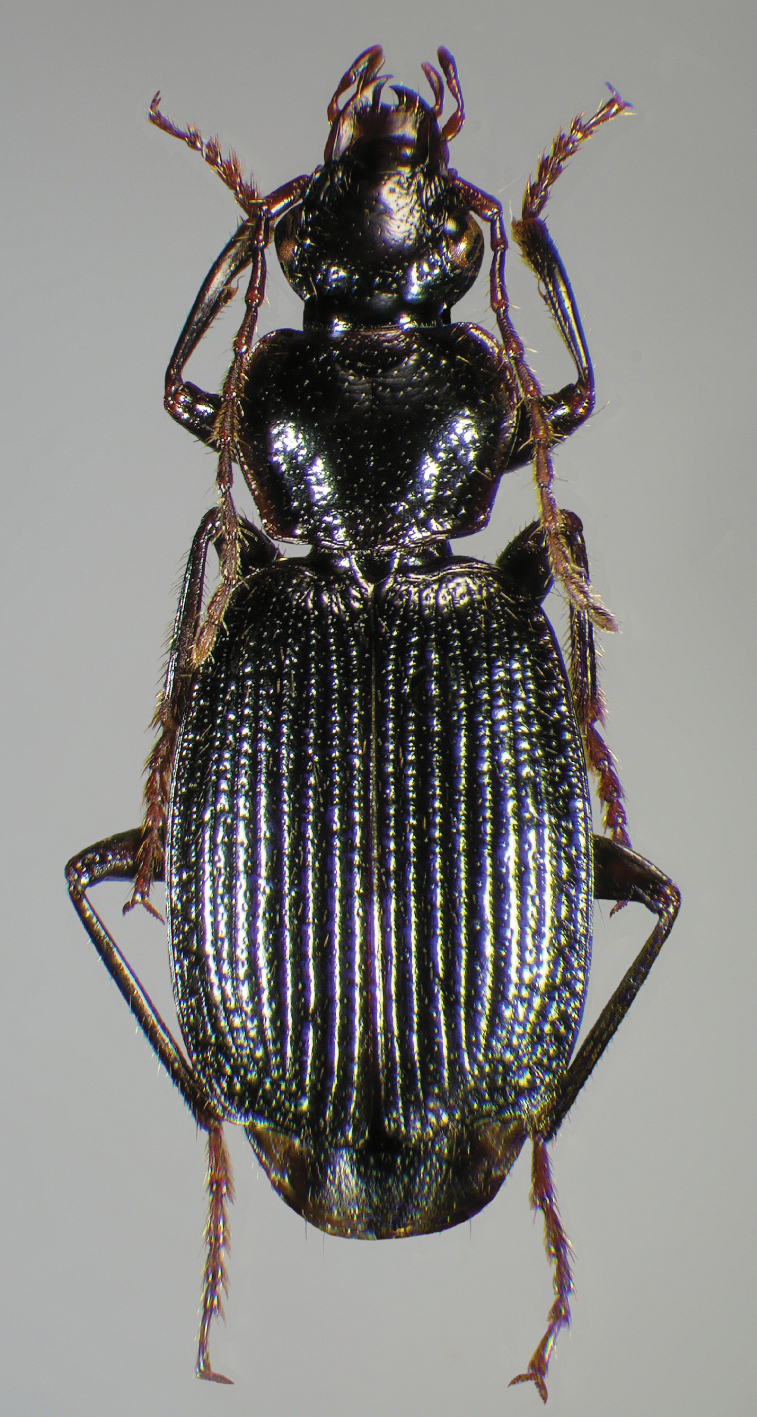
Dorsal habitus and color pattern of *Cymindis ruficornis* (Bates) (OBL 11.50 mm).

*Pronotum* (Figs 30, 31A-B, 32–33). Anterior and posterior transverse impressions shallow; median longitudinal impression shallow; posteriolateral angles from almost right angled to almost rounded; posterior margin slightly lobate.

*Head* (Figs 30, 32–33). Eyes and mouthparts typical for Cymindidina.

*Elytra* (Figs 30, 32–33). Humeri narrowly rounded, typical for subgenus *Pinacodera*; striae moderately impressed; lateral margin smooth, rounded and widened preapically; elytral apices truncate.

*Hind wings* (Fig. 39). Brachypterous, somewhat shortened to markedly short.

*Legs*. Males with adhesive vestiture ventrally, two rows of squamo- setae on tarsomeres 1–4 of foreleg and 1–3 of middle leg.

**Figure 34. F34:**
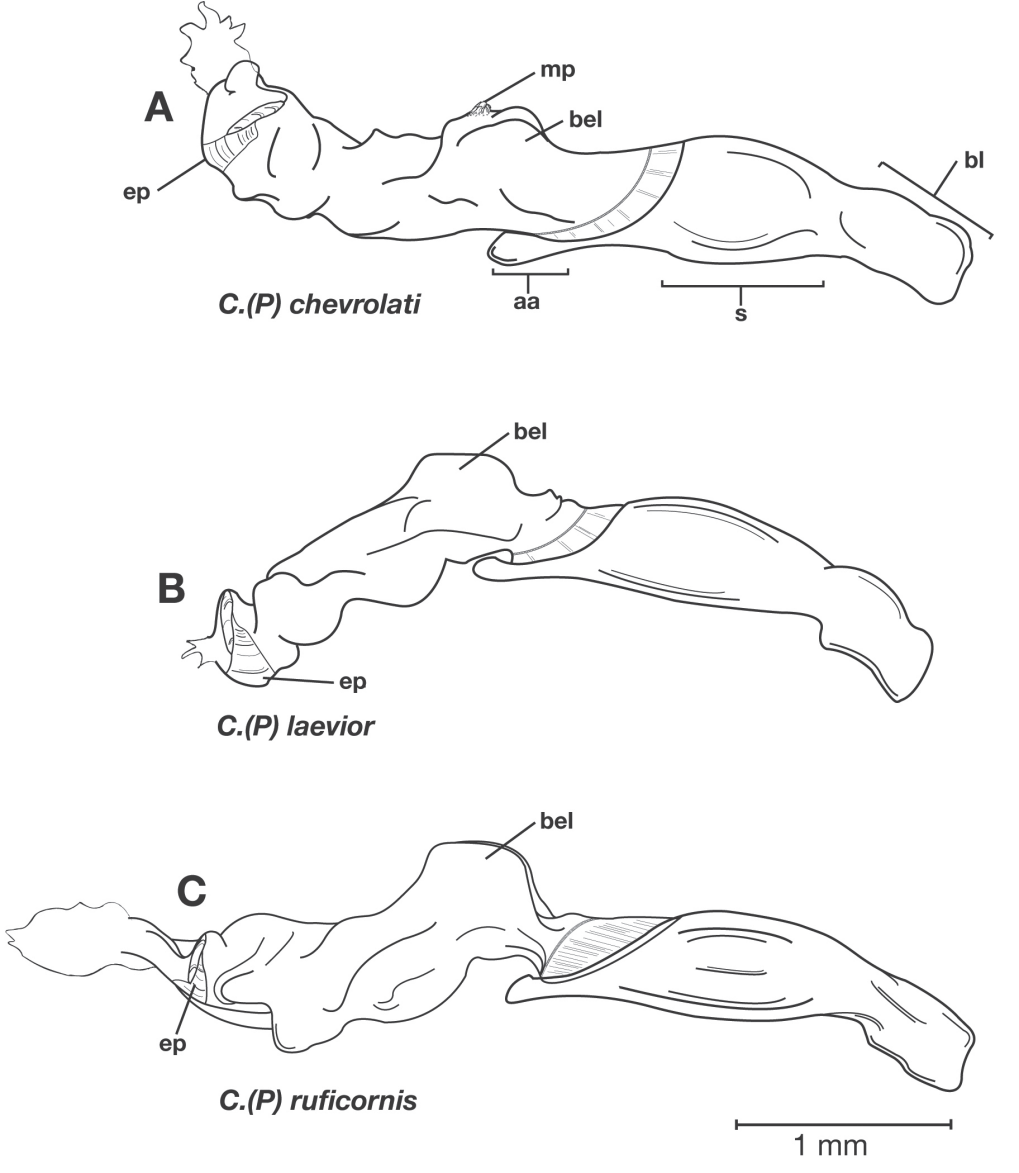
Male genitalia of species of the *chevrolati* complex, right lateral aspect, with endophallus everted: **A**, *Cymindis chevrolati* Dejean; **B**, *Cymindis laevior* (Bates); **C**, *Cymindis ruficornis* (Bates). Legend: **aa**, apical area; **bel**, basal endophallic lobe; **bl**, basal lobe; **ep**, endophallic plate; **mp**, microtrichial patch; **s**, shaft.

*Male genitalia*. Phallus anopic, cylindrical (Fig. 34A-C) ventral surface slightly curved. Endophallus with a slightly curved endophallic plate (**ep**) (Lindroth 1969: 1080–1081) apically. Endophallus with or without microtrichial patch (mp) (Fig. 34, 36) on basal lobe of everted sac.

**Figure 35. F35:**
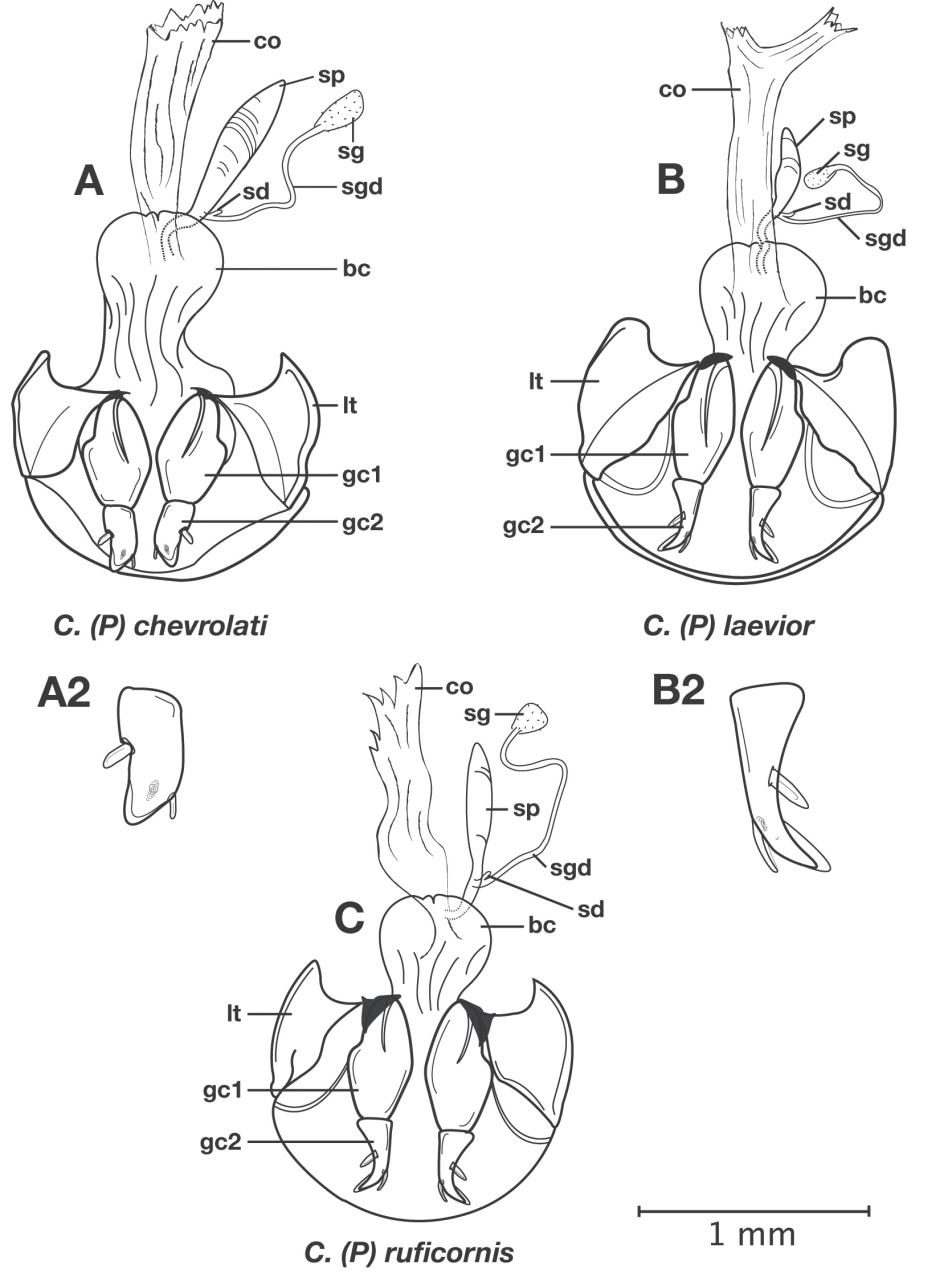
Female reproductive tracts and ovipositors of species of the *chevrolati* complex, ventral aspect: **A**, *Cymindis chevrolati* Dejean; **A2**, gonocoxite 2 enlarged; **B**, *Cymindis laevior* (Bates); **B2**, gonocoxite 2 enlarged; **C**, *Cymindis ruficornis* (Bates). Legend: **bc**, bursa copulatrix; **co**, common oviduct; **gc1**, gonocoxite 1; **gc2**, gonocoxite 2; **lt**, lateral tergite; **sd**, spermathecal diverticulum; **sg**, spermathecal gland; **sgd**, spermathecal gland duct; **sp**, spermatheca.

*Female genitalia*. Gonocoxite 2 (gc2) (Fig. 35) short and stout (Fig. 35A2) to long and narrow (Fig. 35B2). Internal genitalia with long cylindrical spermatheca (sp), associated spermathecal gland (sg), and spermathecal diverticulum (sd) located at base of spermathecal gland duct (sgd).

#### Geographical distribution.

The *chevrolati* complex is known from all the major mountain systems of Mexico north of the Isthmus of Tehuantepec (Fig. 41). It is also known from the Pacific Tres Marias islands.

#### Chorological affinities.

The geographical range of the *chevrolati* complex overlaps the range of one other member of the *limbata* group (*Cymindis punctigera punctigera* LeConte) and the ranges of several species of the *latiuscula* group (Hilchie and Ball in preparation).

#### Taxonomic composition.

Three species are included in this complex: *Cymindis chevrolati* Dejean; *Cymindis laevior* (Bates); and *Cymindis ruficornis* (Bates).

*Overall body length*. Twenty individuals (f=10, m=10) from state population samples of each species in the *chevrolati* complex were compared (Fig. 38). In each species males are shorter on average then females. Overall body length between *Cymindis chevrolati* from Durango and *Cymindis laevior* from Oaxaca, while statistically significant, was not useful taxonomically because of extensive overlap in both males and females.

*Hind wing length*. The three species in the *chevrolati* complex are brachypterous, showing metathoracic wing reduction, from slight (Fig. 39B) to extensive (Fig. 39A). Population samples (Fig. 40) were measured to determine possible trends in wing length. Overall, wing length (Fig. 40) of *Cymindis chevrolati* is the most variable of the three species both between and within populations. Of individuals examined, *Cymindis laevior* had the lowest variation throughout its range, though a slight decrease in length is observed from north to south. Specimens of *Cymindis ruficornis* had the shortest average wing length.

In the range of distributional overlap between *Cymindis chevrolati* and *Cymindis laevior*, wing length serves as a diagnostic character between the two species in Hidalgo (Fig. 40). In populations from Puebla, however, some overlap does occur.

Metepisternum reduction is associated with wing reduction in Carabidae (Darlington 1943). *Cymindis chevrolati* has a metepisternum that is 1.66 to 2.00× longer than wide, *Cymindis laevior* is 1.40 to 1.67× longer than wide and *Cymindis ruficornis* is 1.35 to 1.45× longer than wide. This is strongly correlated with the reduction of wing length observed in *chevrolati* complex members.

Several hypotheses have been proposed for the evolution of brachyptery in insects (Darlington 1943, Kavanaugh 1985, Liebherr and Hajek 1986). *Cymindis laevior* and *Cymindis ruficornis* (the postulated sister group and closest relative to *Cymindis chevrolati*) have reduced wings that can be explained as a result of high altitude occurrence and relatively stable montane habitat. *Cymindis chevrolati* is also found almost exclusively at similar elevations in montane habitats but has a much greater range in wing length. It is unclear what selective pressures may be affecting wing length in *Cymindis chevrolati*. No geographical trends are apparent.

### Cymindis (Pinacodera) chevrolati

Dejean

http://species-id.net/wiki/Cymindis_chevrolati

Figs 30 –31 , 34A , 35A , 36 , 37B-D , 39 [Fig F40] –41

Cymindis atrata Chevrolat 1835, fasc. 7, No. 152 (not Dejean 1831: 327).— TYPE MATERIAL in OXUM: four specimens (2 males, 2 females) three of which are in front of the following green box label: “Cymindis/ Chevrolati/ Dj Cat-3, p.9/ atrata Chv Col Mus 1835/ fasc 7- 152/ Mexico/ D. Sallé “ [handwritten]. LECTOTYPE (here designated) male, labeled: [small green square]; “Chevrolat/ Carabidae/ Fr. V. d. Poll/ Pres. 1909, E./ B. Poulton.” PARALECTOTYPES, (three, here designated), two of which in front of above box label: male, [two lines of illegible handwriting]; “Chevrolat/ Carabidae/ Fr. V. d. Poll/ Pres. 1909, E./ B. Poulton”; female, “Cymindis nigrita Ch”/ “Mexico” [? handwritten]; “Chevrolat/ Carabidae/ Fr. V. d. Poll/ Pres. 1909, E./ B. Poulton”. Paralectotype 3, female, in front of green box label: Cymindis/ nigrita Chd Bull. Mosc./ 1837-VII p. 6/ Mexico D. Sallé” [handwritten]. Specimen labeled: “Cymindis/nigrita Chaud./ Mexique”; “Chevrolat/ Carabidae/ Fr. V. d. Poll/ Pres. 1909, E./ B. Poulton”. – Dejean 1836: 9. –Bates 1883: 187, and 1891: 270.
Cymindis chevrolatii Dejean 1836: 9 [replacement name for *Cymindis atrata*Chevrolat 1835]. – Chaudoir 1873: 54.
Cymindis nigrita Chaudoir 1837: 6–8 [unnecessary replacement name for *Cymindis atrata*Chevrolat 1835]. – Chaudoir 1873: 54, and 1875: 4.
Cymindis amblygona Bates 1878: 606. TYPE MATERIAL: not seen. TYPE AREA: Mexico.
Cymindis angulifera Bates 1878: 606. TYPE MATERIAL: not seen. TYPE AREA: Mexico.
Pinacodera atrata var. *amblygona*Bates 1883: 187.
Pinacodera atrata var. *angulifera*Bates 1883: 187.
Pinacodera chevrolati Csiki 1932: 1487. – Blackwelder 1944: 62.

#### Type locality.

Cruz Blanca, Veracruz, Mexico.

#### Specific epithet.

The original spelling is “*chevrolatii*”, but relatively recent catalogues (Csiki 1932, Blackwelder 1944, Lorenz 2005), have omitted the terminal “i”, thus “*chevrolati*”. Such a modification produces an incorrect subsequent spelling, but I accept the catalogue entries as “prevailing usage”, and the latter name as a correct original spelling (International Code of Zoological Nomenclature 1999: 43, art. 33.3.1).

#### Notes about types, homonymy, and synonymy.

The description of *Cymindis atrata* by Chevrolat (1835: fasc. 7, No. 152) was based on a few specimens collected near Cruz Blanca, Veracruz, by “Sallé”.

As implicitly indicated by Dejean (1836: 9), the name *Cymindis atrata* Chevrolat was a junior homonym of *Cymindis atrata* Dejean (1831, a species that was subsequently transferred to the genus *Inna* Putzeys (Chaudoir 1872) and then to *Eucheila* Dejean (Shpeley and Ball 2001). As a replacement name, Dejean proposed *Cymindis chevrolatii*, using as a voucher specimen a male received from Louis Reiche, labeled “chevrolatii mihi/ atrata Chevrolat/ Mexico, d. Reiche “ [green paper, handwritten]. This specimen is in the Chaudoir/ Oberthür collection, in front of the box label “nigrita Chaudoir” (MNHP). That specimen might have come originally from the Chevrolat collection to Reiche, and then to Dejean (ultimately to Chaudoir). Chaudoir (1837: 8) recorded that he had received specimens from Chevrolat of *Cymindis atrata*, which he renamed *Cymindis nigrita*, evidently being unaware that Dejean had provided a new name in 1836. These four specimens (3 males, 1 female, each labeled “Ex Musaeo/ Chaudoir” [red print] ), are in front of the box label “nigrita / Chaud./ Mexique/ Cruz Blanca/ Sallé” (MNHP). As demonstrated by similarity in the male genitalia, the male lectotype is conspecific with the Dejean voucher for *Cymindis chevrolatii*, and for *Cymindis atrata* Chevrolat.

Authentic specimens of *Cymindis amblygona* Bates and *Cymindis angulifera* Bates were not located in neither The Natural History Museum (London) nor the Museum National d’Histoire Naturelle (Paris), though they were sought in both of those collections that are known to house the Bates material on which his New World work was based. Bates (1883: 187) noted that the features he had originally used to distinguish these two forms were exceedingly variable, even within single populations, citing as an example the series collected at Tehuacan, Mexico. Regarding *Cymindis amblygona*, he stated that “even the definition of the form as a variety is impossible”. Possibly, then, he removed the type labels from what were the type specimens of these forms. Bates described two additional varieties of “*Cymindis atrata*”: *Cymindis atrata ruficornis* and *Cymindis atrata laevior*. These are treated below, as separate species.

#### Diagnosis.

**Figure 36. F36:**
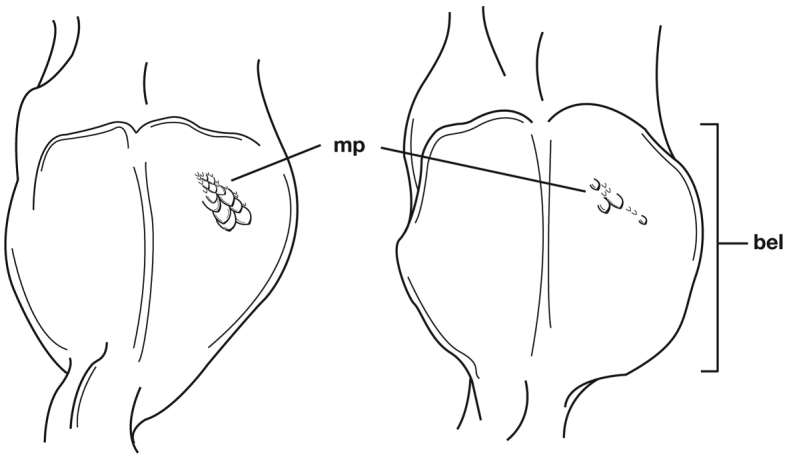
Dorsal bulb of endophallus of *Cymindis chevrolati* Dejean, showing extremes of intraspecific variation in the dorsal microtrichial patch. Legend: **bel**, basal endophallic lobe; **mp**, microtrichial patch.

Adults of *Cymindis chevrolati* (Fig. 30) are distinguishable from those of other species of the *limbata* species group through genitalic characters: in males a distinct microtrichial patch (**mp**) on the basal endophallic lobe (**bel**) of the endophallus (Figs 34A, 36). This patch can be seen in many males through the cleared phallus with endophallus inverted, located near the apex of the phallus. From the right lateral aspect, an everted sac has the microtrichial patch located on the dorsal surface of the basal lobe of the endophallus. Female genitalia differ from other species in the short, stout form of gonocoxite 2 (**gc2**) (Fig. 35, A2).

#### Description.

With character states of subgenus *Pinacodera* and *chevrolati* complex restricted as follows: OBL 10.3 – 13.5 mm. Length (n= 10 males, 10 females): head 1.00 – 1.24, pronotum 1.96 – 2.56, elytra 5.41 – 7.08, metepisternum 1.10 – 1.70 mm; width: head 2.04 – 2.60, pronotum 2.48 – 3.28, elytra 3.83 – 5.16, metepisternum 0.66 – 1.02 mm.

*Body proportions*. HW/HL 1.88 – 2.31; PWM/PL 1.26 – 1.37; EL/EW 1.25- 1.43; ML/MW 1.66 – 2.00.

*Color* (Fig. 30). Dorsum of head black, rarely rufo-piceous in front of eyes; legs piceous to rufo-piceous.

*Microsculpture*. Elytra with mesh pattern isodiametric, microlines shallow in basal half of elytra and shallow to absent from apical half.

*Macrosculpture and pilosity*. Dorsal punctures with setae present though short and almost not visible at 50× magnification. Ventral surface of head with evenly scattered setigerous punctures (bearing somewhat long setae) from behind eye laterally toward mentum. Pronotum normally with relatively evenly scattered setigerous punctures throughout (Fig. 31A), more densely so toward margins; few specimens with setigerous punctures along margin and few to no punctures on disc (Fig. 31B). Elytral epipleuron glabrous.

*Fixed setae*. Pronotum with two fixed setae along each margin. Four to six setae (typically four) along apical margin of sternum VII (Fig 3).

*Luster*. Elytra glossy in basal two thirds, in some specimens slightly less so in apical third.

**Figure 37. F37:**
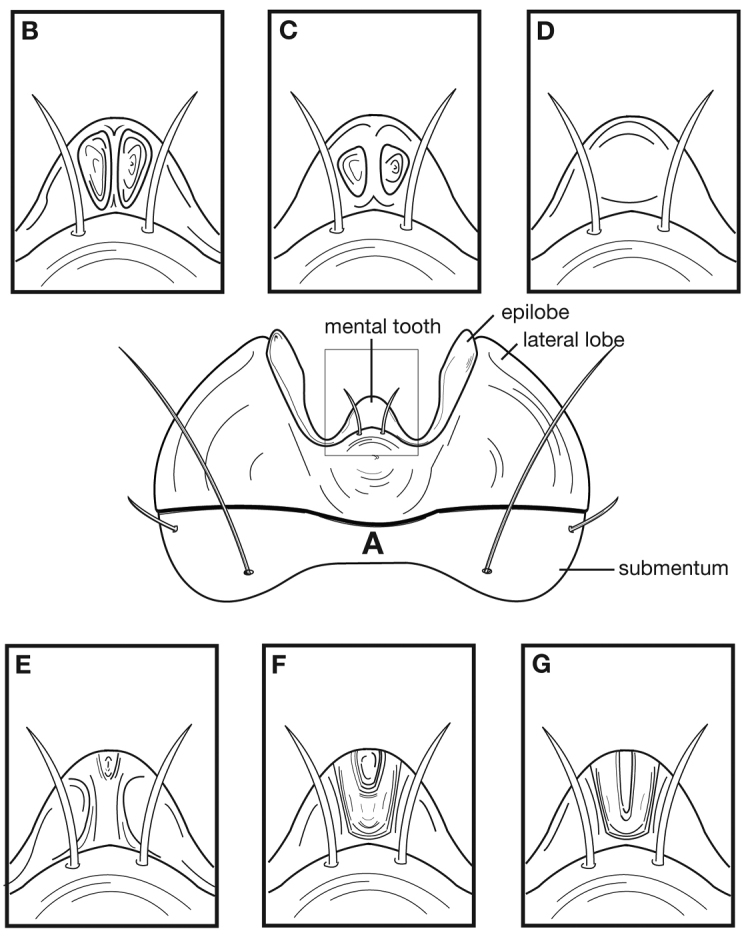
Basal sclerites of labium, ventral aspect, and mental tooth variation in *chevrolati* complex: **A**, submentum and mentum; **B**-**D**, mental tooth of *Cymindis chevrolati* Dejean; **E**-**G**, and *Cymindis laevior* (Bates).

*Head* (Fig. 37B-D). Mental tooth form varied.

*Elytra* (Fig. 30). Humeri narrowly rounded.

*Hind wings*. Somewhat to markedly reduced (Fig. 39). Length 1.34 – 3.29 mm, mean 2.28 mm.

*Male genitalia*. Phallus (Fig. 34A) length 2.20–2.48 mm. Endophallus with microtrichial patch on basal lobe of everted sac (Fig. 36).

*Female genitalia*. Gonocoxite 2 (gc2) (Fig. 35A) short and stout.

#### Collection notes and habitat.

The known elevational range of *Cymindis chevrolati* extends from sea level to 3400 m, though it is important to note that specimens found at or near sea level were collected from the Tres Marias Islands, the immediately adjacent mainland and one other additional record from El Ebano, San Luis Potosi, that may be mislabeled or misinterpreted by me as Ebano, San Luis Potosi. Typically this species is found further inland and at higher elevation, mostly between 2000–3000 m. Specimens were collected under and around woody debris and stones in forests of oak, pine, fir, juniper and alder, in thorn scrub, and in stands of yucca. As well, the species occupies meadow, desert and grassland habitats.

#### Geographical distribution

(Fig. 41) The range of this species is restricted to Mexico, extending in the Sierra Madre Occidental from Chihuahua south to Jalisco, in the Sierra Madre Oriental from central Nuevo Leon south to Hidalgo, and in the Transvolcanic Sierra of central Mexico as far south as central Puebla.

#### Morphological affinities.

Based on genitalic characteristics and wing length states (Fig. 40), I postulate that *Cymindis chevrolati* is the closest relative of *Cymindis laevior* + *Cymindis ruficornis*.

#### Chorological affinities.

*Cymindis chevrolati* is sympatric in a portion of its range with *Cymindis laevior* in the southernmost portion of its range (Fig. 41). It is also sympatric with *Cymindis punctigera punctigera* in the La Michilia area of southern Durango.

#### Material examined.

I examined 662 specimens: 38 males and 26 females were dissected. For details see University of Alberta Strickland Virtual Entomology Museum Database (University of Alberta 2009).

### Cymindis (Pinacodera) laevior

(Bates) stat. n.

http://species-id.net/wiki/Cymindis_laevior

**Figure 38. F38:**
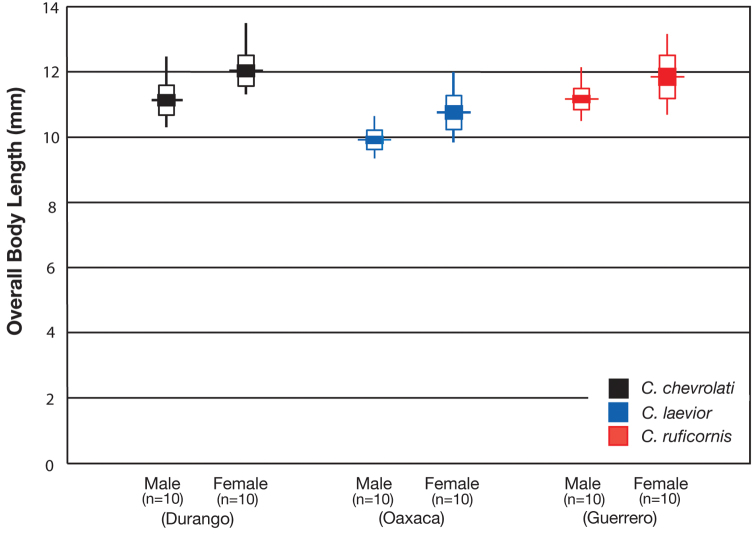
Hubbs-Perlmutter diagram illustrating overall body length (mm) variation in population samples of *Cymindis chevrolati* Dejean, *Cymindis laevior* (Bates), and *Cymindis ruficornis* (Bates). Horizontal lines show mean; vertical lines indicate sample range; white + colored boxes indicate 1.5 standard deviations each side of the mean; and colored boxes indicate 2 standard errors each side of the mean.

Figs 32 , 34B , 35B , 37E-G , 38 , 40 –41

Pinacodera atrata var. *laevior*Bates 1891:270. TYPE MATERIAL: 7 specimens. LECTOTYPE: male, here selected, labeled: “Type/ HT” [circular, ringed with red]”; “Huitzo/ Oaxaca/ Höge; “Tr. Ent. S. L., 1891/ Pinacodera/atrata, Chevr./v. laevior/Bates [handwritten]”; “1891-64”; Pinacodera/ atrata var./ laevior/ Bates” [handwritten]; [BMNH]. TYPE LOCALITY: Huitzo, Oaxaca, Mexico.

#### Diagnosis.

Adults of *Cymindis laevior* (Fig. 32) are distinguishable from those of other species of the *chevrolati* complex through a combination of a glabrous dorsal surface and genitalic characters: males without a microtrichial patch on the basal endophallic lobe (bel) of the aedeagus (Fig. 34B) and female gonocoxite 2 (**gc2**) long and narrow (Fig. 35B).

#### Description.

With character states of subgenus *Pinacodera* and *chevrolati* complex restricted as follows: OBL 9.33 – 12.00 mm. Length (n= 10 males, 10 females): head 0.92 – 1.04, pronotum 1.80 – 2.28, elytra 4.83 – 6.25, metepisternum 0.86 – 1.1 mm; width: head 1.80 – 2.24, pronotum 2.20 – 3.04, elytra 3.67 – 4.75, metepisternum 0.60 – 0.66 mm.

*Body proportions*. HW/HL 1.92 – 2.33; PWM/PL 1.18 – 1.35; EL/EW 1.27 – 1.45; ML/MW 1.40 – 1.67.

*Color* (Fig. 32). Dorsum of head black to rufo-piceous; legs rufo- piceous.

*Microsculpture*. Elytra with mesh pattern isodiametric, microlines shallow throughout length.

*Macrosculpture and pilosity*. Dorsal punctures with setae present, very short, hardly visible at 50×. Head ventrally with sparse, scattered setigerous punctures from behind eye laterally toward mentum. Pronotum normally with relatively evenly scattered setigerous punctures throughout, more densely so toward margins; few specimens with setigerous punctures along margin and few to no punctures on disc. Elytral epipleuron glabrous.

*Fixed setae*. Pronotum with two setae along each margin. Four to six setae (typically four) along apical margin of sternum VII (Fig. 3).

*Luster*. Elytra glossy throughout.

*Head* (Fig. 37E-G). Mental tooth form varied.

*Hind wings*. Markedly reduced, 1.06–1.57 mm in length, mean 1.32 mm.

*Male genitalia*. Phallus (Fig. 34B) length 2.40–2.60 mm.

*Female genitalia*. Gonocoxite 2 (gc2) (Fig. 35B) long and narrow.

#### Collection notes and habitat.

The known elevational range of *Cymindis laevior* extends from 1524 to 3400 m. Specimens have been collected under bark, in woody debris and in leaf litter associated with forests of oak, pine, alder, juniper and stands of yucca. They have also been collected from bromeliads growing on standing trees. Because adults are incapable of flight, their presence above ground indicates the ability to climb trees.

#### Geographical distribution

(Fig. 41) The range of this species is restricted to Mexico, extending from northern Hidalgo in the eastern Transvolcanic Sierra east to western Veracruz and west to eastern Jalisco. The range extends in the Sierra Madre del Sur southward through the Sierra Madre de Oaxaca south to the Sierra de Miahuatlan from southern Oaxaca, and as far west as western Oaxaca.

**Evolutionary affinities.** Based on genitalic characteristics and wing length states, I postulate that *Cymindis laevior* is the closest relative of *Cymindis ruficornis*.

**Chorological affinities.** *Cymindis laevior* is sympatric in the northern most portion of its range with *Cymindis chevrolati* (Fig. 41).

**Material examined.** I have examined 202 specimens; 34 males and 20 females dissected. For details see University of Alberta Strickland Virtual Entomology Museum Database (University of Alberta 2009).

### Cymindis (Pinacodera) ruficornis

(Bates) stat. n.

http://species-id.net/wiki/Cymindis_ruficornis

Figs 33 , 34C , 35C , 38 , 40 –41

Pinacodera atrata var. *ruficornis*Bates 1891:270. TYPE MATERIAL: 30 specimens. LECTOTYPE: female, here selected, labeled: “Type/ HT” [circular, ringed with red]”; “Omilteme/ Guerrero/ July H.H. Smith”; “Tr. Ent. S. L., 1891/ Pinacodera atrata Chevr./v. ruficornis, Bates”; “1891-64”; Pinacodera/ atrata var./ ruficornis [handwritten] [BMNH]. TYPE LOCALITY: Omiteme, Guerrero, Mexico.

#### Diagnosis.

Adults of *Cymindis ruficornis* (Fig. 33) are distinguished from those of the other species of the *chevrolati* complex by erect pilose setae covering the entire dorsal surface, one to three long setae on the each lateral pronotal margin, in addition to the typical fixed setae, and their restricted geographical distribution (Fig. 41).

**Figure 39. F39:**
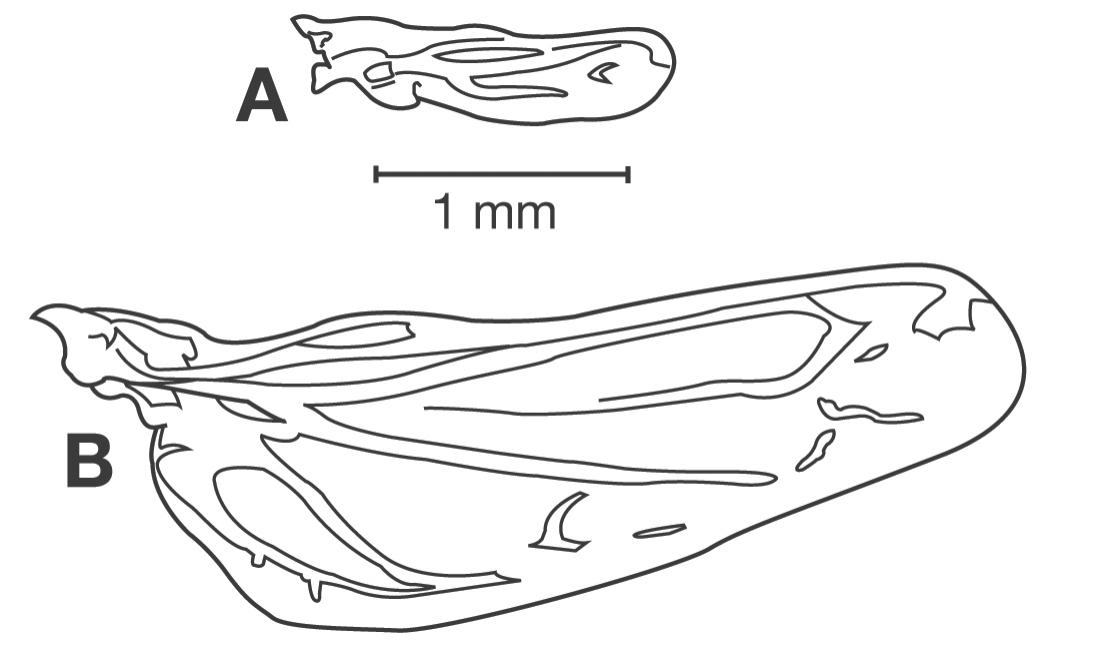
Right hindwing of *Cymindis chevrolati* Dejean, dorsal aspect, showing extremes of interpopulation variation: **A**, markedly atrophied; **B**, slightly atrophied.

#### Description.

With character states of subgenus *Pinacodera* restricted as follows: OBL 10.50 – 13.17 mm. Length (n= 10 males, 10 females): head 0.92 – 1.20, pronotum 2.04 – 2.60, elytra 5.42 – 7.17, metepisternum 1.08 – 1.20 mm; width: head 1.96 – 2.56, pronotum 2.76 – 3.56, elytra 4.17 – 5.42, metepisternum 0.70 – 0.84 mm.

*Body proportions*. HW/HL 2.03 – 2.33; PWM/PL 1.31 – 1.41; EL/EW 1.18 – 1.37; ML/MW 1.35 – 1.45.

**Figure 40. F40:**
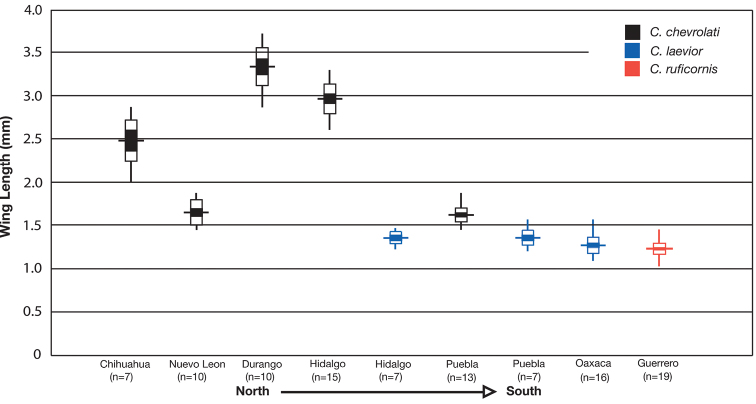
Hubbs-Perlmutter diagram illustrating wing length (mm) variation in Mexican state population samples of *Cymindis chevrolati* Dejean, *Cymindis laevior* (Bates), and *Cymindis ruficornis* (Bates). Horizontal lines show mean; vertical lines indicate sample range; white + colored boxes indicate 1.5 standard deviations each side of the mean; and colored boxes indicate 2 standard errors each side of the mean.

*Color* (Fig. 33). Dorsum of head and pronotum piceous; head typically rufo-piceous from hind margin of eyes forward; legs piceous to rufo- piceous; epipleuron and abdominal sterna rufo-piceous to piceous.

*Microsculpture*. Elytra with mesh pattern isodiametric, microlines shallow in most specimens, in few specimens more so in basal third.

*Macrosculpture and pilosity*. Dorsal punctures with setae present, erect and easily visible at low magnification. Ventral surface of head with evenly scattered setigerous punctures (bearing somewhat long pilose setae) from behind eye laterally toward mentum. Pronotum with evenly scattered punctures and pilose setae over entire dorsal surface. Elytral epipleuron with scattered punctures and pilose setae, setae longer and more regular in apical half.

*Fixed setae*. Pronotum with two to five fixed setae along lateral margin; elytra with two setae in stria 3 and 16 to 18 umbilical setae; two setae on each of abdominal sterna III to VI; four to eight setae along apical margin of sternum VII, typically six.

*Luster*. Elytra glossy.

*Hind wings*. Markedly reduced. Length 1.09–1.49 mm, mean 1.28 mm.

*Male genitalia*. Phallus (Fig. 34C) length 2.42 – 2.56 mm.

*Female genitalia*. Gonocoxite 2 (gc2) (Fig. 35C) long and narrow.

#### Collection notes and habitat.

The known elevational range of *Cymindis ruficornis* extends from 1980 to 2774 m. Specimens have been collected in leaf litter and under wood and stones in forests of oak, pine, fir, and alder.

#### Geographical distribution.

**Figure 41.**
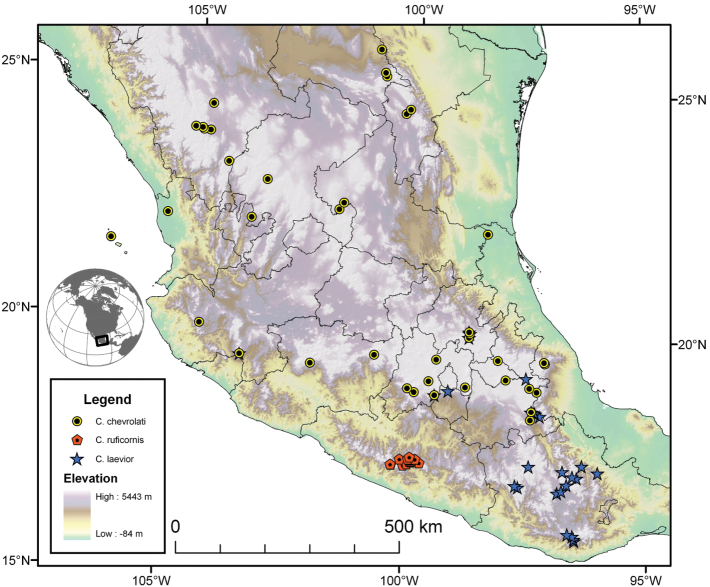
Map of extreme southeastern Texas, U.S.A. and Mexico north of the Isthmus of Tehuantepec, showing position of localities for species of the *chevrolati* complex.

The known range of this species is restricted to the easternmost portion of the Sierra de Atoyac, in eastern Guerrero (Fig. 41).

#### Morphological affinities.

Based on genitalic characteristics and wing length states (Fig. 40), I postulate that *Cymindis ruficornis* is the closest relative of *Cymindis laevior*.

#### Chorological affinities.

*Cymindis ruficornis* is allopatric in relation to the other members of the *chevrolati* complex and all other members of the *limbata* species group.

#### Material examined.

I have examined 116 specimens; 10 males and 10 females were dissected. For details see University of Alberta Strickland Virtual Entomology Museum Database (University of Alberta 2009).

## Concluding remarks

It it not often that as much material is available (over 4000 specimens) as was for this revision. The large number of both borrowed and collected specimens allowed for examination of most species of the *limbata* group. throughout their ranges. Beyond determining how many species are in the *limbata* group and their geographical distributions, other interesting findings were made in regard to habitat preferences, geographical variation, macroptery/bachyptery, and possible hybridization.

Field collecting and integration of available label data has allowed insight into the special niche requirements of *Cymindis complanata*. All specimens of *Cymindis complanata* collected for this revision (13) were taken from slash pine (*Pinus elliotii* Engelm.) in mixed forest. Some borrowed specimens were also recorded from loblolly pine (*Pinus taeda* L.), which is a very similar species of tree. Having evolved a flattened, reddish body that blends well into the flaky bark of these trees, it would appear that *Cymindis complanata* utilizes these trees specifically. This is very interesting as no other members of the *limbata* group seem to have habitat preferences that are so specific.

I was very fortunate to examine nearly 900 specimens of *Cymindis platicollis platicollis*, from localities throughout its range. This allowed for an examination of possible patterns of geographical variation. Consequently, a survey of the external morphology exposed a northeast to southwest cline of increasing dorsal seta length that coincided with body size increase and elytral puncture change. With this pattern understood, 2 new synonomies were uncovered that may have otherwise gone unnoticed.

The availablilty of large number of specimens of *Cymindis punctigera punctigera* also allowed for a thorough examination of the wing states of individuals throughout its range. An interesting pattern of macroptery/brachyptery was revealed, with brachypterous individuals typically being found near the outer limits of the range and macropterous individuals in the center. Most brachypterous individuals of *Cymindis punctigera punctigera* live in the Mojave, Sonoran and Chihuahuan deserts, so desert associated conditions almost certainly play a role in this observed pattern. Wing reduction or loss has been tied to hot, dry, desert conditions in members of several insect taxa (Brues 1903; Peck 2008). This is partly because wing surfaces provide a major outlet for water loss and because of this, are more readily selected against in areas with these condition (Tinaut 1992). More work has to be done however, to determine what other mitigating factors may be influencing the observed pattern.

Analysis of genitalic characters, wing length, mental tooth form, and body size variation affirmed the presence of three *chevrolati* complex. As well, dissections of many males of both *Cymindis chevrolati* and *Cymindis laevior* from their narrow range of overlap revealed that some individuals had a highly reduced (few small microtrichia) microtrichial patch on the basal endophallic lobe. This appears to be an intermediate genitalic feature between the two species as *Cymindis laevior* has no microtrichial patch and members of *Cymindis chevrolati* have a patch with several microtrichia. Interestingly, the majority individuals with a reduced microtrichial patch were also observed to have a mental tooth form that was also “intermediate”. This suggests the possibility that gene flow between *Cymindis chevrolati* and *Cymindis laevior* may be occurring in their area of geographic overlap.

Many of these observations would not have been possible without the substantial amount of specimens available for this revision. This illustrates the importance of having as much material as possible when doing revisionary work. In the future, the incorporation of molecular methods will likely help to further refine phylogenetic relationships within the *limbata* group. It may also aid in an examination of gene flow in the areas of sympatry between species.

## Supplementary Material

XML Treatment for Cymindis (Pinacodera)

XML Treatment for Cymindis (Pinacodera) limbata

XML Treatment for Cymindis (Pinacodera) limbata

XML Treatment for Cymindis (Pinacodera) complanata

XML Treatment for Cymindis (Pinacodera) limbata

XML Treatment for Cymindis (Pinacodera) platicollis

XML Treatment for Cymindis platicollis platicollis

XML Treatment for Cymindis platicollis atripennis

XML Treatment for Cymindis (Pinacodera) rufostigma

XML Treatment for Cymindis (Pinacodera) punctigera

XML Treatment for Cymindis punctigera

XML Treatment for Cymindis punctigera punctigera

XML Treatment for Cymindis punctigera sulcipennis

XML Treatment for Cymindis (Pinacodera) chevrolati

XML Treatment for Cymindis (Pinacodera) chevrolati

XML Treatment for Cymindis (Pinacodera) laevior

XML Treatment for Cymindis (Pinacodera) ruficornis
